# Chemical Protein Engineering: Backbone Cyclization Rescues Folding of a 183‐Residue Truncated Domain of Malaria Parasite Protein *Pf*AMA1

**DOI:** 10.1002/chem.202500894

**Published:** 2025-04-21

**Authors:** Jamsad Mannuthodikayil, Vishal Malik, Abhisek Kar, Sameer Singh, Kalyaneswar Mandal

**Affiliations:** ^1^ Tata Institute of Fundamental Research Hyderabad 36/p Gopanpally Hyderabad Telangana 500046 India

**Keywords:** chemical protein synthesis, malaria, native chemical ligation, protein engineering, protein‐protein interactions

## Abstract

The interaction between apical membrane antigen 1 (*Pf*AMA1) and rhoptry neck protein 2 (*Pf*RON2) is crucial for *Plasmodium falciparum* red blood cell invasion, making it a key target for anti‐malarial drug development strategies. Here, we report the chemical synthesis of *Pf*AMA1 domain I (*Pf*AMA1‐DI) in both linear and backbone‐circularized forms, employing a six‐segment convergent synthesis approach exploiting one‐pot chemistries and solubilizing tags. The chemically synthesized linear *Pf*AMA1‐DI construct exhibited incomplete disulfide bond formation during folding, likely due to increased terminal flexibility in the absence of domain II. To address this, we employed backbone cyclization of the large 180‐residue polypeptide chain, with 3‐residue linker sequence, as a unique strategy to conformationally restrict its termini and facilitate correct disulfide bond formation. Introducing a multipurpose affinity and solubility tag to the cyclic*Pf*AMA1‐DI construct further improved the folding yield by mitigating aggregation. The predicted structure using ColabFold‐Alphafold2 indicated that *Pf*RON2 ligand binds within the hydrophobic groove of the cyclic*Pf*AMA1‐DI construct similar to the native interactions. These findings underscore the potential of large protein backbone cyclization to stabilize protein structure, offering a compelling strategy for the chemical synthesis of otherwise unstable protein domains with broad applications in miniature protein engineering.

## Graphical abstract

We report the first chemical synthesis of a 183‐residue functional truncated cyclic *Pf*AMA1 domain I protein using multi‐segment native chemical ligation and head‐to‐tail cyclization to introduce conformational constraints, thereby facilitating protein folding and providing novel strategies for miniature protein engineering. Institute and/or researcher Twitter usernames: TIFRH_buzz

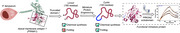

## 1 Introduction

Malaria, caused by protozoan parasites of the *Plasmodium* genus, remains a significant global health challenge, with *Plasmodium falciparum* responsible for the majority of severe malaria cases, exceeding half a million deaths annually.^[^ ^1^ ^]^ Two parasite proteins, apical membrane antigen 1 (*Pf*AMA1) and rhoptry neck protein 2 (*Pf*RON2), are critical for the invasion of red blood cells by *P. falciparum*. *Pf*AMA1 is positioned on the merozoite surface, while *Pf*RON2 integrates into the red blood cell membrane during invasion. These two proteins form a complex known as the moving junction, vital for the parasite's entry into and survival within the host cells.^[^ ^2^ ^]^

*Pf*AMA1, consisting of 598 residues and three distinct domains, primarily interacts with *Pf*RON2 through its domain I (DI) and domain II (DII). During moving junction formation, *Pf*RON2 ectodomain binds to a hydrophobic cleft in *Pf*AMA1, a region highly conserved across *P. falciparum* isolates, underscoring its importance for parasite viability.^[^ ^3^ ^]^ Given the lack of alternative invasion pathways, disrupting this protein‐protein interaction presents an attractive strategy for anti‐malarial therapies aimed at preventing red blood cell invasion without eliciting drug resistance.

Several inhibitors targeting the *Pf*AMA1‐*Pf*RON2 interaction have been explored, including small molecules,^[^ ^4^ ^]^ phage display‐derived peptides,^[^ ^5^ ^]^ and inhibitors based on the beta‐sheet loop^[^ ^6^ ^]^ of the native *Pf*RON2 ectodomain. Structural studies of the *Pf*AMA1‐*Pf*RON2_2021‐2059_ complex reveal that *Pf*RON2 ectodomain binds within a hydrophobic cleft in *Pf*AMA1 domain I, flanked by six loops from domain I and an extended loop from domain II (DII loop).^[^ ^7^ ^]^ The domain II, loop spanning Ala346 to Lys395 in particular, is crucial for maintaining *Pf*AMA1 domain I's structural stability, as it occupies the hydrophobic groove in domain I when *Pf*RON2 is absent.^[^ ^8^ ^]^ Furthermore, replacing the DII loop with a Gly‐Ser linker (ΔDII(loop)‐*Pf*AMA1) modestly reduces the binding affinity of the *Pf*RON2 ectodomain peptide (*Pf*RON2_2021‐2059_), suggesting that domain I alone may be sufficient for binding with *Pf*RON2_2021‐2059_.^[^ ^8^ ^]^

While recombinant expression systems have been employed to express functional *Pf*AMA1‐(DI+DII),^[^ ^8^, ^9^ ^]^ the chemical synthesis of *Pf*AMA1 offers unique advantages. These include the ability to incorporate non‐natural amino acids, homogeneous chemical modifications, and tailored customizations as and when required. Our aim here is to establish a synthetic method for producing functional *Pf*AMA1 domain I only, which can later be used to generate its enantiomer (D‐enantiomer) for mirror‐image biological display – a technique widely employed to identify D‐peptide and protein inhibitors.^[^ ^10^ ^]^ D‐target proteins can only be realized via chemical protein synthesis,^[^ ^11^ ^]^ making this approach particularly challenging for exploring D‐protein therapeutics.

Chemical synthesis of a large protein, which involves synthesizing multiple peptide segments via solid phase peptide synthesis (SPPS) ^[^ ^12^ ^]^ and assembling them in solution through native chemical ligation (NCL),^[^ ^13^ ^]^ presents several challenges. Strategies such as orthogonal cysteine protection,^[^ ^14^ ^]^ desulfurization,^[^ ^15^ ^]^ peptide thioesters,^[^ ^16^ ^]^ solubilizing tags^[^ ^16^, ^17^ ^]^ and fast‐flow synthesis^[^ ^18^ ^]^ have been developed to facilitate the chemical synthesis of proteins. However, synthesizing large, functional proteins like the 336‐residue extracellular domains (DI+DII) of *Pf*AMA1 remains challenging due to their size and the complexity associated with the formation of the right combination of disulfide bonds.

**Figure 1:**
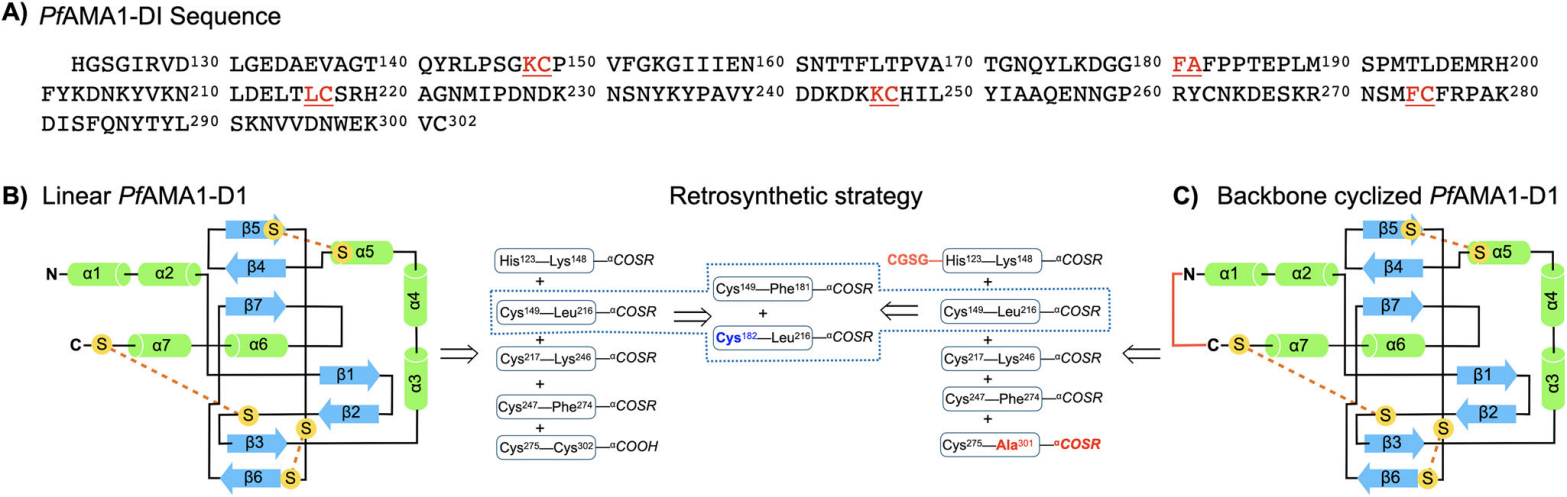
Retrosynthetic strategy. A) Amino acid sequence of the truncated *Pf*AMA1‐DI [His123‐Cys302, UniProtKB– Q7KQK5], with NCL sites underlined (−X−C−, red). B,C) Schematic representation of the retrosynthetic strategy for the synthesis of both linear (B) and cyclic (C) PfAMA1‐DI. The graph representations of the structures are color coded by secondary structure: beta sheets (sky blue), helices (green), and other regions (black); sulfur atoms are highlighted in yellow, and disulfide connections are represented in red dotted lines.

To overcome these challenges, we focused on synthesizing a functional miniature version of *Pf*AMA1, specifically domain I, which contains the hydrophobic groove essential for *Pf*RON2 binding. Previous studies using recombinant protein had shown that ΔDII(loop)‐*Pf*AMA1 (*Pf*AMA1‐DI&II, des domain II loop) retained nanomolar affinity for *Pf*RON2_2021‐2059_,^[^ ^8^ ^]^ suggesting domain I alone is a potential target for chemical synthesis. Based on the *Pf*AMA1 crystal structure (PDB: 1Z40),^[^ ^19^ ^]^ we selected a truncated 180‐residue segment from domain I (His123 to Cys302) as a target for the chemical synthesis (Figure 1A). This segment includes the hydrophobic binding groove and all three native disulfide bonds, offering the potential for correct folding to a functional *Pf*AMA1‐DI.

However, synthesizing domain I alone risks misfolding due to the absence of the stabilizing domain II, particularly the DII loop. The disulfide bond between Cys149 and Cys302 is of particular concern, as increased flexibility at the N‐ and C‐terminal regions in the absence of domain II could lead to incorrect disulfide bond formation and protein aggregation. To address this, we explored backbone (head‐to‐tail) cyclization as a strategy to enhance structural rigidity and promote correct disulfide bond formation (Figure 1). Backbone cyclization, achieved by introducing an eupeptide bond between the N‐ and C‐termini, conformationally locks the protein and avoids non‐native interactions of terminal residues that can lead to misfolding.

Notably, the largest functional protein synthesized via backbone cyclization to date is the 70‐residue bacteriocin As‐48.^[^ ^21^ ^]^ Although synthesizing a cyclic 180‐residue protein is challenging, we hypothesized that cyclization could enhance the conformational stability of *Pf*AMA1‐DI, promoting correct disulfide bond formation and ensuring proper folding. By synthesizing both linear and circularized forms of *Pf*AMA1‐DI, we aim to directly compare the impact of cyclization on protein folding efficiency and binding affinity. This approach also offers insights into the potential of head‐to‐tail cyclization for stabilizing miniature proteins, paving the way for efficient D‐target synthesis for mirror‐image biological display.

## 2 Results and Discussion

### 2.1 Synthetic Strategy

To synthesize both the linear and cyclic 180‐residue *Pf*AMA1‐DI, we employed a convergent^[^ ^22^ ^]^ synthesis strategy using NCL. The polypeptide was divided into five segments based on the availability of native cysteines required for NCL (Figure 1A). However, the second segment, spanning 66 residues, lacked a cysteine for further segmentation, and our attempts to synthesize this long segment through SPPS resulted in a very low yield and poor purity of the crude peptide. To overcome this limitation, we replaced Ala182 with Cys182, allowing the segment (Seg) to be divided into two shorter fragments, Seg‐2 and Seg‐3 (Figure 1B,C). After synthesis and ligation, Cys182 was reverted back to the native Ala182 using desulfurization chemistry. For the synthesis of Seg‐4+5+6, an N‐to‐C sequential ligation approach was initially employed. Seg‐4 and Seg‐5 were synthesized as peptide hydrazides^[^ ^16b,c^ ^]^ with Cys217 in Seg‐4 orthogonally protected using acetamidomethyl (Acm) group for orthogonal ligation, while Seg‐6 required no termini modification.

**Figure 2:**
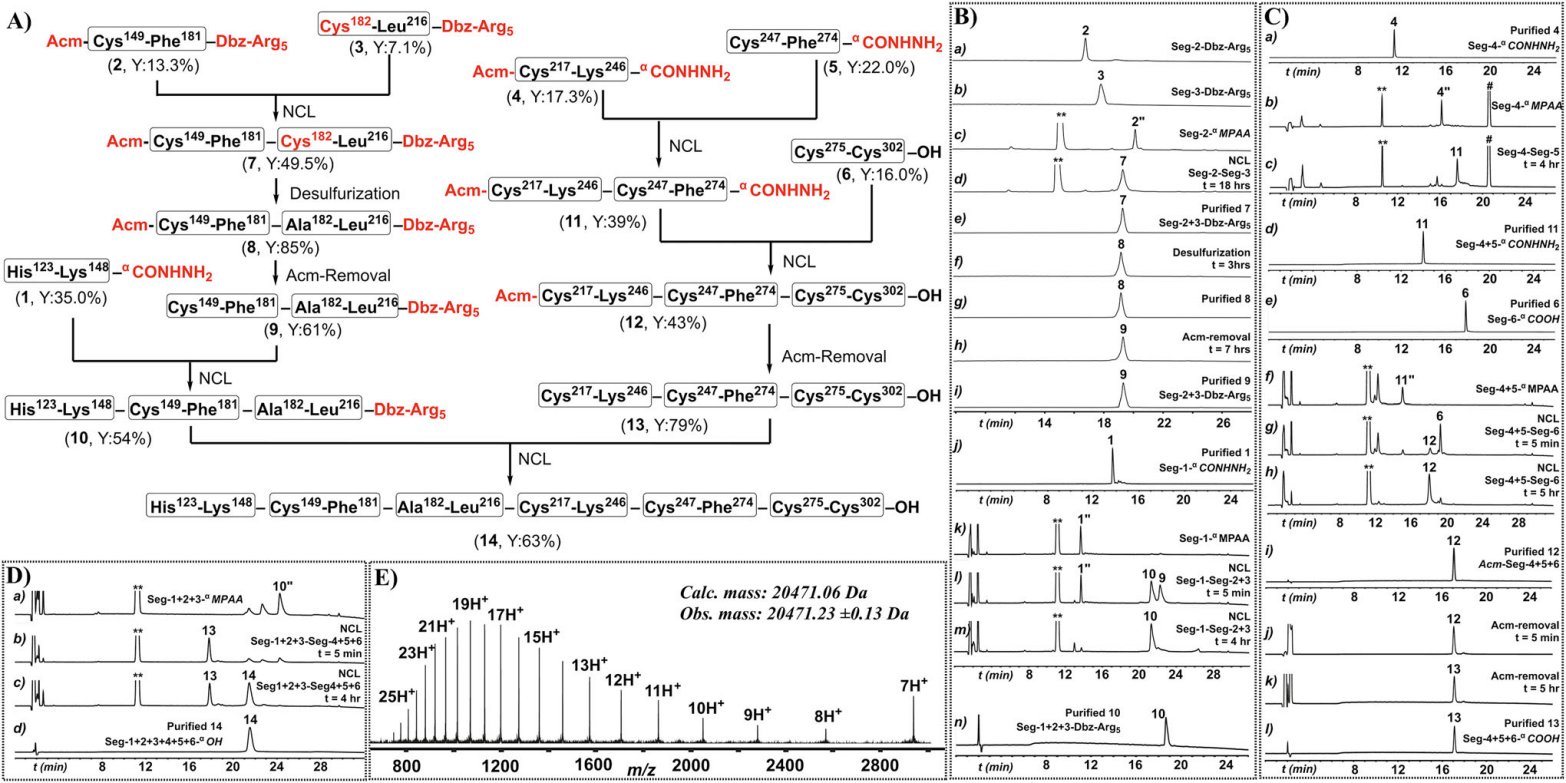
Chemical synthesis of *Pf*AMA1‐DI polypeptide. A) Convergent synthetic strategy for linear *Pf*AMA1‐DI. B) Analytical HPLC chromatogram (λ = 214 nm) of: a) purified Seg‐2 **2**; b) purified Seg‐3 **3**; c) reaction mixture after MPAA α‐thioester formation **2″** from peptide **2** (200 mm phosphate, 6 m Gu.HCl, 20 mm NaNO_2_, 100 mm MPAA); d) reaction mixture after 18‐h ligation between peptides **2″** and **3**, yielding peptide **7**; e) purified peptide **7**; f) desulfurization of peptide **7** (at 3‐h time point in buffer containing 75 mm glutathione, 150 mm TCEP, and 100 mM VA‐044 at 42 °C forming product **8**); g) purified peptide **8**; h) Acm removal from 0.5 mm peptide **8** in 7 h using 25 mm AgOAc in 1:1 AcOH:H_2_O buffer to yield peptide **9**; i) purified peptide **9**; j) purified Seg‐1 **1**; k) MPAA α‐thioester formation **1″** from peptide **1**; l) ligation mixture of peptides **1″** and **9** forming the ligated product **10**; m) product after 4‐h ligation, yielding peptide **10**; n) purified peptide **10**. C) HPLC chromatogram (λ = 214 nm) of: a) purified Seg‐4 **4**; b) MPAA α‐thioester formation from peptide **4**; c) ligation product **11** after 4 h; d) purified peptide **11**; e) purified Seg‐6 **6**; f) MPAA α‐thioester formation from peptide **11**; g) ligation mixture of peptides **6** and **11″**; h) 5‐h ligation yielding peptide **12** (Linear gradient 20–40% of B over 20‐min followed by 40–60% of B in 5‐min including 5 min initial equilibration was used for the chromatographic separation); i) purified peptide **12 (**Linear gradient 10–54% of B over 22 min including 4 min equilibration was used for the chromatographic separation); j) Acm removal from peptide **12**; k) peptide **13** after Acm removal in 5 h; l) purified peptide **13**. D) HPLC chromatogram of: a) MPAA α‐thioester formation from peptide **10**; b) 5 min after addition of peptide **13** to peptide **10″**; c) ligation completed in 4 h, yielding peptide **14**; d) purified peptide **14**. E) ESI‐mass spectrum of purified unfolded *Pf*AMA1‐DI **14** (calculated mass: 20471.06 Da, observed: 20471.23 ± 0.13 Da). MPAA = HS‐C_6_H_4_CH_2_COOH; “**” denotes MPAA; “*” indicates TCEP adduct with dibenzofulvene; “**#**” indicates MPAA disulfide dimer.

**Figure 3:**
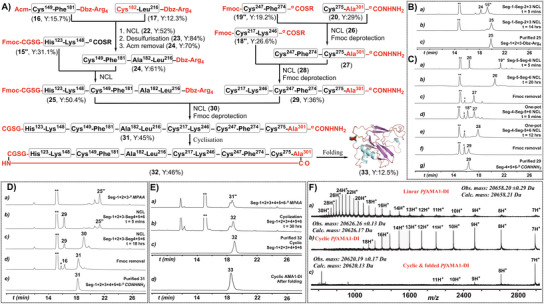
Chemical synthesis of cyclic*Pf*AMA1‐DI. A) Convergent synthetic strategy for cyclic*Pf*AMA1‐DI, with its AlphaFold2‐predicted folded structure shown as a cartoon. R = −CH_2_CH_2_SO_3_Na. B) HPLC monitoring (λ = 214 nm) of the synthesis of the N‐term half: a) 5‐min reaction of peptides **15″** and **24** in ligation buffer (200 mm phosphate, 6 m Gu.HCl, 20 mm MPAA, pH 6.8); b) ligation after 14 h yielding polypeptide **25**; c) purified polypeptide **25**. C) HPLC monitoring (λ = 214 nm) of the synthesis of the C‐term half: a) reaction of peptides **19″** and **20** in ligation buffer (200 mm phosphate, 6 m Gu.HCl, 50 mm MPAA, pH 6.85) after 5‐min; b) ligation completed in 20 h yielding polypeptide **26**; c) Fmoc deprotection of peptide **26** yielding peptide **27**; d) reaction of peptide **27** with peptide **18″** after 5‐min; e) ligation completed in 12 h yielding peptide **28**; f) Fmoc deprotection of peptide **28** yielding peptide **29**; g) purified polypeptide **29**. D) HPLC monitoring (λ = 214 nm) of the ligation of N‐term half and the C‐term half: a) formation of MPAA thioester of peptide **25** yielding peptide **25″** in 200 mm phosphate, 6 m Gu.HCl, 25 mm NaNO_2_, 100 mm MPAA; b) reaction mixture at 5‐min after addition of peptide **29** to peptide **25″** in ligation buffer; c) reaction mixture after 18‐h ligation yielding peptide **30**; d) Fmoc deprotection of peptide **30** yielding peptide **31**; e) purified peptide **31**. E) HPLC monitoring (λ = 214 nm) of the cyclization step: a) MPAA thioester formation of peptide **31** yielding peptide **31″**; b) cyclization completed in 30 h yielding polypeptide **32**; c) purified cyclic polypeptide **32**; d) folding of polypeptide **32** in redox buffer (20 mm phosphate, 1 mm GSH, 0.1 mm GSSG, 0.5 m Gu.HCl, 100 mm NaCl, pH 8.2, 4 °C), yielding folded cyclic protein **33**. F) ESI‐MS spectrum of: a) purified linear *Pf*AMA1‐DI **31** (calculated mass: 20658.21 Da, observed: 20658.20 ± 0.29 Da); b) purified unfolded cyclic*Pf*AMA1‐DI **32** (calculated mass: 20626.17 Da, observed: 20626.26 ± 0.13 Da); c) purified folded cyclic*Pf*AMA1‐DI **33** (calculated mass: 20620.13 Da, observed: 20620.19 ± 0.17 Da). MPAA = HS‐C_6_H_4_CH_2_COOH; “**” indicates MPAA; “*” denotes the TCEP adduct with dibenzofulvene.

Our retrosynthetic strategy for both the linear and cyclic *Pf*AMA1‐DI synthesis required modification only in Seg‐1 and Seg‐6, with the remaining four peptide segments being identical for both versions (Figure 1). The convergent approach using these strategies required separate synthesis of Seg‐1+2+3 and Seg‐4+5+6, followed by ligating them to yield the full‐length polypeptide (Figures 2A and 3A).

An evaluation of the Grand Average of Hydropathicity (GRAVY)^[^ ^23^ ^]^ of peptide segments revealed that Seg‐2 exhibited higher hydrophobicity, which increased the risk of aggregation during its handling in aqueous conditions. To counter this, we incorporated a polyarginine (PolyArg)^[^ ^17c,d^ ^]^ solubility tag at the C‐terminus of Seg‐2, and a similar tag was added to Seg‐3 to mitigate potential handling challenges during the synthesis of Seg‐2+3. Additionally, Cys149 in Seg‐2 was orthogonally protected with acetamidomethyl (Acm) to allow Seg‐2+3 ligation and subsequent desulfurization (Figures 2A and 3A).

### 2.2 Synthesis of Peptide Segments

All peptide segments were synthesized using Fmoc‐based SPPS^[^ ^12a^ ^]^ employing heat‐assisted *N,N'*‐diisopropylcarbodiimide (DIC)/ Ethyl cyanohydroxyiminoacetate (Oxyma) coupling chemistry^[^ ^24^ ^]^ at elevated temperatures (up to 65 °C) to improve coupling efficiency, followed by Fmoc deprotection at ≤50 °C (see Section S1.3, Supporting Information). To avoid aspartimide formation, which can occur in Asp‐Gly and Asn‐Gly sequences, we used Fmoc‐(Dmb)Gly‐OH during the synthesis of Seg‐2 and Seg‐5. PolyArg tags were attached to peptides via *o*‐aminoanilide chemistry with Boc‐protected Dbz,^[^ ^16d^ ^]^ which successfully prevented the formation of benzimidazolinone byproducts during synthesis at elevated temperatures, resulting in clean peptides, Seg‐2 and Seg‐3. We synthesized Seg‐1, Seg‐4, and Seg‐5 as peptide α‐hydrazides to serve as α‐thioester surrogates for their respective ligations.^[^ ^16b,c^ ^]^ During the synthesis of Seg‐5, significant methionine oxidation (Met(O)) was observed at Met273, which was reversed via a previously reported^[^ ^25^ ^]^ orthogonal chemical reduction protocol (see Section S8.0, Supporting Information). The chemical synthesis of *Pf*RON2_2021‐2059_ peptide used in these studies was reported elsewhere (see Section S9.1, Supporting Information).^[^ ^9a^ ^]^

### 2.3 Chemical Synthesis of Linear *Pf*AMA1‐DI

The chemical synthesis of the linear *Pf*AMA1‐D1 polypeptide is depicted in Figure 2. We began the synthesis by preparing six corresponding peptide segments via Fmoc‐SPPS: Seg‐1 (**1**, His^123^–Lys^148^‐^α^CONHNH_2_), Seg‐2 (**2**, Acm‐Cys^149^–Phe^181^‐^α^Dbz‐Arg_5_‐^α^CONH_2_),^[^ ^16d^ ^]^ Seg‐3 (**3** (A182C), Cys^182^–Leu^216^‐^α^Dbz‐Arg_5_‐^α^CONH_2_), Seg‐4 (**4**, Acm‐Cys^217^–Lys^246^‐^α^CONHNH_2_), Seg‐5 (**5**, Cys^247^–Phe^274^‐^α^CONHNH_2_), and Seg‐6 (**6**, Cys^275^–Cys^302^‐^α^COOH),^[^ ^14d^ ^]^ with purified yields of 35%, 13.3%, 7.1%, 17.3%, 22%, and 16%, respectively.

The synthesis of Seg‐1+2+3 polypeptide was initiated with the ligation of Seg‐2 and Seg‐3. Peptide **2** was activated with NaNO_2_ (20 mm) at −16.5 °C, followed by the addition of MPAA (100 mm) to generate a peptide‐Cα‐MPAA‐thioester **2″**. Ligation with peptide **3** at pH 6.8 for 18 h produced the ligated product, polypeptide **7**, with an isolated yield of 49.5%. Radical‐mediated desulfurization^[^ ^15^ ^]^ was then performed on polypeptide **7** to revert Cys182 to Ala182 using VA044 (100 mm), TCEP (150 mm), and glutathione (75 mm) in phosphate buffer (200 mm PB, 6 m Gu.HCl, pH 7) at 42 °C for 3 h, yielding native (Ala182) polypeptide **8** in 85% yield. Acm deprotection of Cys‐149 was achieved using AgOAc (25 mm)^[^ ^26^ ^]^ in a 1:1 AcOH/H_2_O solution for 7 h at 30 °C, followed by quenching with DTT, resulting in polypeptide **8** (Seg‐2+3) with an isolated yield of 61%. Subsequently, Seg‐1 (**1**) was ligated with Seg‐2+3 (**8)**, after converting peptide **1** to a C^α^‐MPAA‐thioester **1″** using NaNO_2_ and MPAA. The ligation was carried out at pH 6.8 for 4 h, yielding the fully ligated product Seg‐1+2+3 (**10**, His^123^–Leu^216^‐^α^Dbz‐Arg_5_‐^α^CONH_2_) with 54% yield after purification.

For the synthesis of the latter half of the polypeptide, Seg‐4 (**4**) was converted to a C^α^‐MPAA‐thioester **4″** via NaNO_2_ activation and thiolysis with MPAA, and ligated with Seg‐5 (**5**) at pH 6.9 for 4 h, yielding Seg‐4+5 (**11**) with a 39% isolated yield. This product was similarly converted to a C^α^‐MPAA‐thioester **11″** and ligated with Seg‐6 **6** at pH 6.8 for 5 h, giving polypeptide **12** in a 43% isolated yield. Acm deprotection of Cys‐217 was then performed using AgOAc, followed by DTT quenching, resulting in Seg‐4+5+6 (**13**) with a 79% isolated yield. However, the overall yield of the synthesis by N‐to‐C ligation approach was only 13%.

To improve the efficiency of Seg‐4+5+6 synthesis, a one‐pot C‐to‐N sequential ligation approach was employed using Fmoc‐masked N‐terminal cysteines.^[^ ^14d^ ^]^ In brief, Fmoc‐Seg‐4‐MESNa **18″** and Fmoc‐Seg‐5‐MESNa **19″** were prepared by converting the respective peptide hydrazides to thioesters. Peptide **19″** was ligated with Seg‐6 (**6**) in the presence of MPAA, followed by Fmoc deprotection. The second ligation was performed with peptide **18″**, and final Fmoc deprotection yielded Seg‐4+5+6 (**13**) with a 37% overall yield,^[^ ^14d^ ^]^ exhibiting a threefold improvement over the N‐to‐C approach.

With both polypeptides (Seg‐1+2+3 and Seg‐4+5+6) in hand, we proceeded with the final convergent ligation step. We first activated the polypeptide **10** with NaNO_2_ and exchanged it with MPAA, followed by ligation with polypeptide **13** at pH 6.8 for 4 h, resulting in the full‐length polypeptide **14** (*Pf*AMA1‐DI, His^123^–Cys^302^‐^α^COOH) with a 63% isolated yield. The homogeneity and mass of the synthesized 180‐residue *Pf*AMA1‐DI polypeptide **14** was confirmed by LC‐MS (Figure 2F, observed mass: 20471.23 ± 0.13 Da; calculated mass: 20471.06 Da (average isotope)). Despite the successful synthesis, attempts to fold the full‐length polypeptide using various redox buffers and folding conditions were unsuccessful. HPLC‐MS analysis showed a +2 Da mass deviation from the expected fully folded product, indicating incomplete disulfide bond formation, likely due to the absence of domain II, leading to increased flexibility at its termini. At this juncture, we anticipated that enhancing structural rigidity via cyclization of the N‐ and C‐termini could facilitate the proper folding of this 180‐residue polypeptide.

### 2.4 Chemical Synthesis of Cyclic‐*Pf*AMA1‐DI

To achieve backbone cyclization of the *Pf*AMA1‐DI and maintain its native tertiary structure by avoiding strain from the cyclization, a Gly‐Ser‐Gly (GSG) linker was introduced between His123 (N‐terminus) and Cys302 (C‐terminus). This approach followed the same six‐segment synthesis strategy used for the linear form, with cyclization performed in the final step to promote correct folding. To facilitate the cyclization via NCL, Cys302 in Seg‐6 was relocated to the N‐terminus of Seg‐1, and Val301 was replaced with Ala (V301A) to avoid NCL‐based cyclization challenges caused by the β‐branched residue. Suitably, a Cys‐Gly‐Ser‐Gly sequence was added to the N‐terminus of His123 in Seg‐1 to accommodate both the GSG linker and the N‐terminal cysteine required for cyclization via NCL. Importantly, this retrosynthetic approach required modifications only in Seg‐1 and Seg‐6, while the other segments remained identical to those used in the synthesis of the linear construct (Figures 1 and 3A).

Segments designed for backbone cyclization, peptide 15″ (*modified*‐Seg‐1, Fmoc‐Cys‐Gly‐Ser‐Gly‐His^123^–Lys^148^‐^α^COSCH_2_CH_2_SO_3_Na) and peptide **20** (*modified*‐Seg‐6, V301A/Cys^275^–Ala^301^‐^α^CONHNH_2_) were synthesized and purified with isolated yields of 31.1% and 29%, respectively. The ligation of peptide **15″** (*modified‐*Seg‐1) with Seg‐2+3 (**24**, Cys^149^–Leu^216^‐^α^Dbz‐Arg_4_‐^α^CONH_2_; see Section S5.2, Supporting Information) was carried out in a pH 6.9 ligation buffer containing 200 mm PB, 6 m Gu.HCl, 20 mm MPAA, and 20 mm TCEP. After 14 h of reaction, the ligated product *modified‐*Seg‐1+2+3 polypeptide **25** (Fmoc‐Cys‐Gly‐Ser‐Gly‐His^123^‐Leu^216^‐^α^Dbz‐Arg_4_‐^α^CONH_2_) was obtained with a 50.4% yield after purification.

The synthesis of Seg‐4+5+6 for cyclic*Pf*AMA1‐DI (*modified*‐Seg‐4+5+6, **29**) followed the same one‐pot^[^ ^14d^ ^]^ C‐to‐N ligation strategy previously used in the linear construct synthesis. In brief, peptide **19″** (Seg‐5) and peptide **20** (*modified*‐Seg‐6) were ligated at pH 6.8 to yield **26**, followed by adjustment of the pH to 11 for Fmoc deprotection resulting in polypeptide **27** (*modified‐*Seg‐5+6). The pH was then brought back to 6.9 to facilitate the second ligation with peptide **19″** (Seg‐4) to obtain polypeptide **28** in one pot. After a final Fmoc deprotection step at pH 11, the full‐length *modified‐*Seg‐4+5+6 polypeptide **29** (Cys^217^–Ala^301^‐^α^CONHNH_2_) was obtained with an overall isolated yield of 36% (*see* Figure 3C; Section S5.3, Supporting Information).

Next, *modified‐*Seg‐1+2+3 (**25**) was converted to a C^α^‐MPAA thioester **25″** using NaNO_2_ and MPAA, followed by ligation with *modified‐*Seg‐4+5+6 (**29**) at pH 6.8. The reaction was allowed to proceed for 14 h to get ligated product **30** before adjusting the pH to 11 for Fmoc deprotection. This yielded the full‐length polypeptide **31** (*modified‐*Seg‐1+2+3+4+5+6, Cys‐Gly‐Ser‐Gly‐His^123^–Ala^301^‐^α^CONHNH_2_) with a 45% yield after purification (Figure 3D). The mass of the synthesized polypeptide **31** was confirmed by LC‐MS, with an observed mass of 20658.20 ± 0.29 Da (calc. mass = 20658.21 Da (average isotope)).

For the final head‐to‐tail cyclization step, *modified‐*Seg‐1+2+3+4+5+6 (**31**) was converted to the corresponding Cα‐MPAA thioester **31″** via NaNO_2_ (20 mm) activation, followed by the addition of 50 mm MPAA under dilute conditions to favour intra‐molecular cyclization over polymerization. The reaction mixture was adjusted to pH 6.5 and incubated for 30 h, yielding the backbone circularised *Pf*AMA1‐DI polypeptide **32** with a 46% yield (Figure 3E), confirmed by LC‐MS with an observed mass of 20626.26 ± 0.13 Da (calc. mass = 20626.17 Da (average isotope)). The cyclic polypeptide was then folded to cyclic*Pf*AMA1‐DI (**33**) in a redox buffer (20 mm phosphate, 1 mm GSH, 0.1 mm GSSG, 0.5 m Gu.HCl, 100 mm NaCl, pH 8.22) at 4 °C. The charge distribution pattern and a mass shift of −6 Da from the unfolded polypeptide (Figure 3F) indicated the formation of a folded protein with three disulfide bonds. However, the overall folding yield was limited to 12.5% due to significant aggregation, consistent with previous reports of similarly low yields (∼18%)^[^ ^9a^ ^]^ for the folding of recombinantly expressed 336‐residue *Pf*AMA1‐(DI+DII) under similar aggregation challenges.

The observed low yield is likely due to the formation of kinetically trapped partially folded intermediates with incorrect disulfide bonds, leading to the misfolded and insoluble aggregates. These insoluble aggregates were removed from the folding buffer by centrifugation, followed by dialysis to remove the remaining Gu.HCl and redox reagents. Despite these efforts, attempts to determine the binding affinity (K_D_) of *Pf*RON2_2021‐2059_ to immobilized cyclic*Pf*AMA1‐DI **33** using surface plasmon resonance (SPR) were unsuccessful, likely due to disruptions in the protein's functional fold caused by the surface immobilization (amine coupling) chemistries (see Section S9.2, Supporting Information). To address this, we sought to incorporate an affinity tag to the cyclic*Pf*AMA1‐DI that would enable gentler surface immobilization, ensuring functional *Pf*AMA1‐DI for SPR analysis.

### 2.5 Chemical Synthesis of cyclic*Pf*AMA1‐DI with Affinity Tag

**Figure 4:**
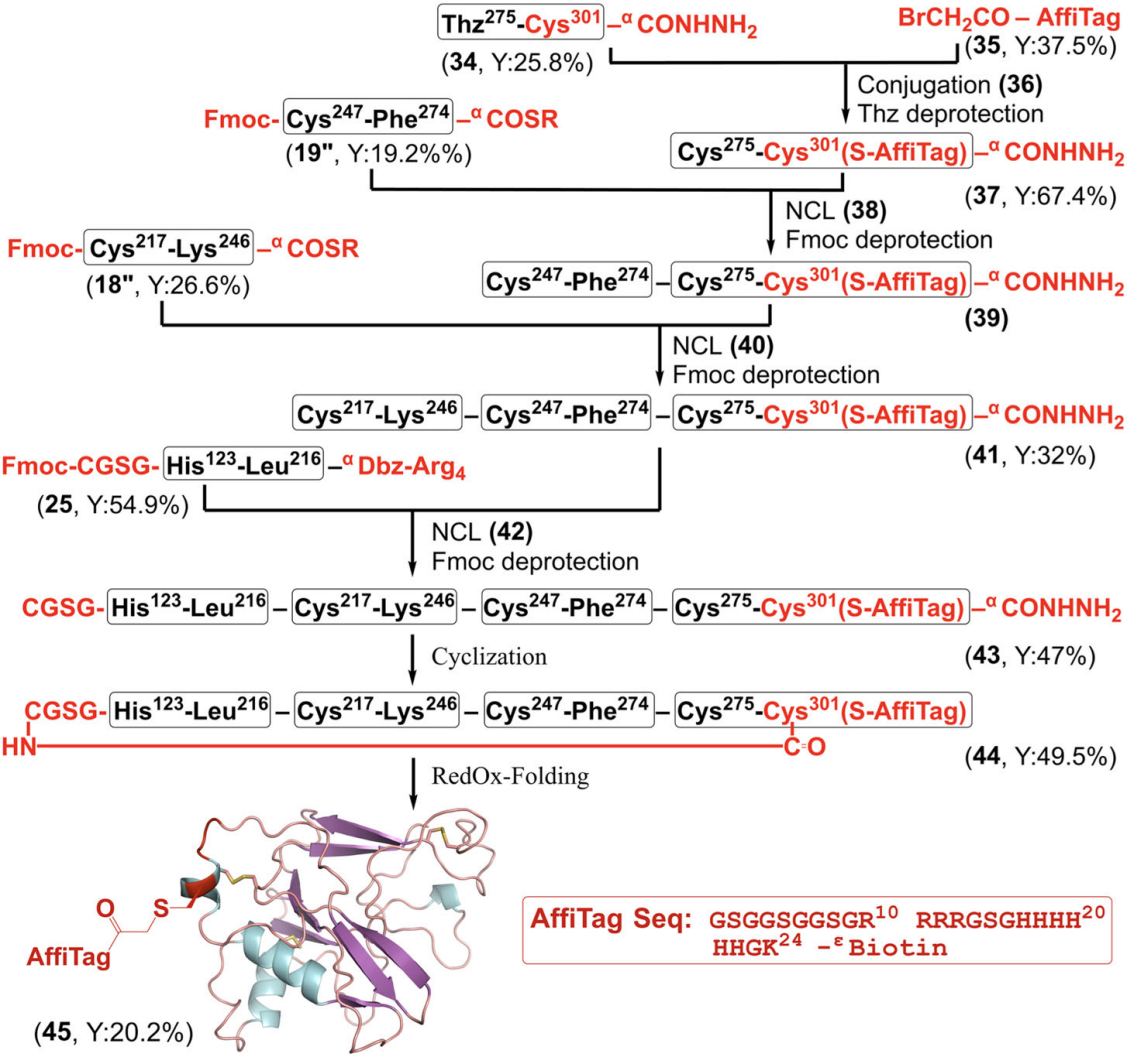
Chemical synthesis of cyclic*Pf*AMA1‐DI with Affinity Tag. Convergent synthetic strategy for the cyclic*Pf*AMA1‐DI (AffiTag) with AffiTag sequence (inset). AlphaFold2‐predicted folded structure with modifications is shown as a cartoon. R = −CH_2_CH_2_SO_3_Na.

To facilitate gentle surface immobilization for SPR binding assays or biological screening purposes, we incorporated a polyhistidine (polyHis) and biotin affinity tag (denoted as AffiTag here) into the Seg‐6 of cyclic*Pf*AMA1‐DI construct, positioned it away from the hydrophobic cleft of *Pf*AMA1. This addition was achieved by conjugating the tag to Cys301 (incorporated by replacing native Val301), introduced in Seg‐6, via a thioether bond. We added a flexible 16‐residue spacer (GSGGSGGSGRRRRGSG) before the polyHis and biotin to ensure *Pf*AMA1‐DI accessibility for solution phase interactions, with a polyArg sequence within the spacer enhancing solubility and reducing aggregation during folding. Biotin was conjugated to the ε‐amino group of a lysine residue located at the C‐terminal end of the His6 tag, separated by a glycine residue (see Figure 4 inset for sequence).

For the tagged Seg‐6 synthesis (*AffiTag*‐Seg‐6), Cys275 was protected as thiazolidine (Thz), allowing selective conjugation of the bromoacetylated (Gly‐Ser‐Gly)₃‐Arg₄‐Gly‐Ser‐Gly‐His_6_‐Gly‐Lys‐^ε^Biotin tag (**35**, bromoacetyl‐AffiTag) to the side chain thiol of Cys301. In this approach, we reused *modified‐*Seg‐1+2+3 **25** from prior cyclic*Pf*AMA1‐DI synthesis, reducing extra synthetic steps. *V301C‐*Seg‐6 (**34**, V301C/Thz^275^–Cys^301^‐^α^CONHNH_2_) and the bromoacetyl‐AffiTag **35** were synthesized with 25.8% and 37.5% isolated yields, respectively. Conjugation of AffiTag peptide **35** to *V301C‐*Seg‐6 **34** was completed within 1 h in pH 8.4 buffer (200 mm phosphate, 6 m Gu.HCl) yielding conjugated polypeptide **36**, followed by Thz deprotection in the same buffer with 100 mm methoxylamine hydrochloride at pH 4.5 for 2 h, yielding tagged Seg‐6 (*AffiTag*‐Seg‐6, **37**, V301C/Cys^275^–Cys^301^(S‐AffiTag)‐^α^CONHNH₂) in 67.4% yield after HPLC purification (see Section S7.1, Supporting Information).

For synthesizing AffiTagged Seg‐4+5+6 (*AffiTag*‐Seg‐4+5+6), we employed the same one‐pot^[^ ^14d^ ^]^ strategy previously used in both the linear and cyclic construct synthesis. Briefly, Seg‐5 **19″** was ligated with *AffiTag*‐Seg‐6 **37** in a ligation buffer, followed by raising the pH to 11 for Fmoc deprotection. The pH was then adjusted back to 6.9 for the second ligation with Seg‐4 **18″** and final deprotection at pH 11 yielded the full‐length *AffiTag*‐Seg‐4+5+6 polypeptide (**41**, Cys^217^–Cys^301^(S‐AffiTag)‐^α^CONHNH_2_) with a 32% overall yield after purification (see Section S7.2, Supporting Information). Next, *modified*‐Seg‐1+2+3 polypeptide **25** was activated using NaNO_2_ and converted to corresponding C^α^‐MPAA thioester **25″**. This was followed by ligation with *AffiTag*‐Seg‐4+5+6 **41** in a pH 6.8 buffer for 24 h. After the Fmoc deprotection at pH 11, the full‐length polypeptide **43** (*AffiTag*‐Seg‐1+2+3+4+5+6, Cys‐Gly‐Ser‐Gly‐His^123^–Cys^301^(S‐AffiTag)‐^α^CONHNH_2_) was obtained with a 47% isolated yield (see Section S7.3, Supporting Information). The mass of the final product was confirmed by LC‐MS, with an observed mass of 23412.47 ± 0.29 Da (calculated mass 23412.22 Da (average isotope)).

For the final backbone cyclization step, *AffiTag*‐Seg‐1+2+3+4+5+6 **43** was converted to the MPAA α‐thioester **43″** under dilute conditions, followed by cyclization at pH 6.5 for 30 h. The tagged cyclic Seg‐1+2+3+4+5+6 (**44**, *AffiTag*‐*cyclic‐*Seg‐1+2+3+4+5+6) was obtained in 49.5% yield after HPLC purification, as confirmed by an observed mass of 23380.33 ± 0.25 Da (calculated mass 23380.18 Da (average isotope)). The *AffiTag*‐*cyclic‐*Seg‐1+2+3+4+5+6 **44** was then folded in a redox glutathione buffer, yielding a fully folded tagged cyclic*Pf*AMA1‐DI **45** with the expected −6 Da mass shift (observed mass: 23374.31 ± 0.19 Da), indicating successful disulfide bond formation (see Section S7.4, Supporting Information). The folding yield was 20.2%, significantly higher than the yield observed for the un‐tagged cyclic*Pf*AMA1‐DI, although significant aggregation still occurred. Protein aggregates were removed by centrifugation, followed by dialysis, to ensure the purity of the final folded product.

To evaluate the functional activity of the folded protein, we examined the binding of the chemically synthesized AffiTagged cyclic*Pf*AMA1‐DI and *Pf*RON2_2021‐2059_ by surface plasmon resonance on a nickel‐NTA chip (BI‐4500, Biosensing Instrument Inc., USA). The binding kinetics yielded an association rate constant (Ka) of (1.42 ± 0.14) × 10^5^ m ^−1^ s^−1^, dissociation rate constant (Kd) of (7.08 ± 0.81) × 10⁻^2^ s⁻¹, and an overall dissociation constant (K_D_) of 500 ± 14 nM (see Section S9.2, Supporting Information). This K_D_ indicates a weaker affinity compared to the recombinant full‐length *Pf*AMA1‐(DI+DII) and ΔDII(loop)‐*Pf*AMA1 (des‐domain II loop), suggesting domain I alone lacks the stability for robust *Pf*RON2_2021‐2059_ binding. Previous studies^[^ ^9a^ ^]^ with recombinant full‐length *Pf*AMA1‐(DI+DII), in contrast, exhibited a K_D_ of ≈22 nM (Ka = 1.49 × 10^5^ m⁻¹s⁻¹; Kd = 3.21 × 10⁻^3^ s⁻¹), underscoring domain II's essential role in enhancing stability of the binary complex by decreasing the dissociation rate.

Prior studies have demonstrated that the DII loop enhances the functional complex's half‐life by 18‐fold by kinetically locking the *Pf*AMA1‐*Pf*RON2 complex.^[^ ^8^ ^]^ Their kinetic traces showed that *Pf*RON2_2021‐2059_ binds to ΔDII(loop)‐*Pf*AMA1 with an association constant (Ka) comparable to its binding with the full‐length *Pf*AMA1‐(DI+DII), however, *Pf*RON2_2021‐2059_ dissociates (Kd) from ΔDII(loop)‐*Pf*AMA1 significantly faster than from the full‐length *Pf*AMA1‐(DI+DII).^[^ ^8^ ^]^ Consistent with these findings, our data show that the association rate of *Pf*RON2_2021‐2059_ with our chemically synthesized engineered cyclic*Pf*AMA1‐DI (Ka = 1.42 × 10⁵ m⁻¹s⁻¹) is similar to that observed with recombinant full‐length *Pf*AMA1‐(DI+DII) (Ka = 1.49 × 10⁵ m⁻¹s⁻¹). However, the dissociation rate (Kd = 7.08 × 10⁻^2^ s⁻¹) of the *Pf*RON2_2021‐2059_‐cyclic*Pf*AMA1‐DI complex is significantly increased, reflecting a weakened overall binding affinity relative to the *Pf*RON2‐*Pf*AMA1‐(DI+DII) complex (Kd = 3.21 × 10⁻^3^ s⁻¹). This rapid dissociation indicates the absence of stabilizing interactions contributed by the entire domain II on *Pf*AMA1‐DI, beyond just the DII loop as seen in ΔDII(loop)‐*Pf*AMA1, resulting in a considerably weaker K_D_ for the *Pf*RON2‐cyclic*Pf*AMA1‐DI complex.

To gain structural insights into cyclic*Pf*AMA1‐DI binding with *Pf*RON2_2021‐2059_, we employed the ColabFold‐AlphaFold2‐CycPepDock, a deep‐learning tool integrated into the Google Colaboratory platform, with a cyclic peptide complex offset model as described by Kosugi et al.^[^ ^20^ ^]^ This protocol applies a cyclic positional encoding for the cyclic peptide domain while preserving the default encoding for the protein region, and achieves high predictive accuracy for head‐to‐tail circularized peptides.^[^ ^27^ ^]^ Although optimized for cyclic peptides, we adapted this tool for cyclic protein‐peptide interactions by applying the cyclic offset to the cyclic*Pf*AMA1‐DI domain while leaving *Pf*RON2_2021‐2059_ with default encoding. From 36 generated models with varied circularly permuted sites (*see* Section S10 and Table S1, Supporting Information), five high‐confidence structures (pLDDT > 90%) were identified, with the top two models achieving pLDDT scores of 91.8%. These five structures of cyclic*Pf*AMA1‐DI/ *Pf*RON2_2021‐2059_ complexes are shown in Figure S34 (Supporting Information).

**Figure 5:**
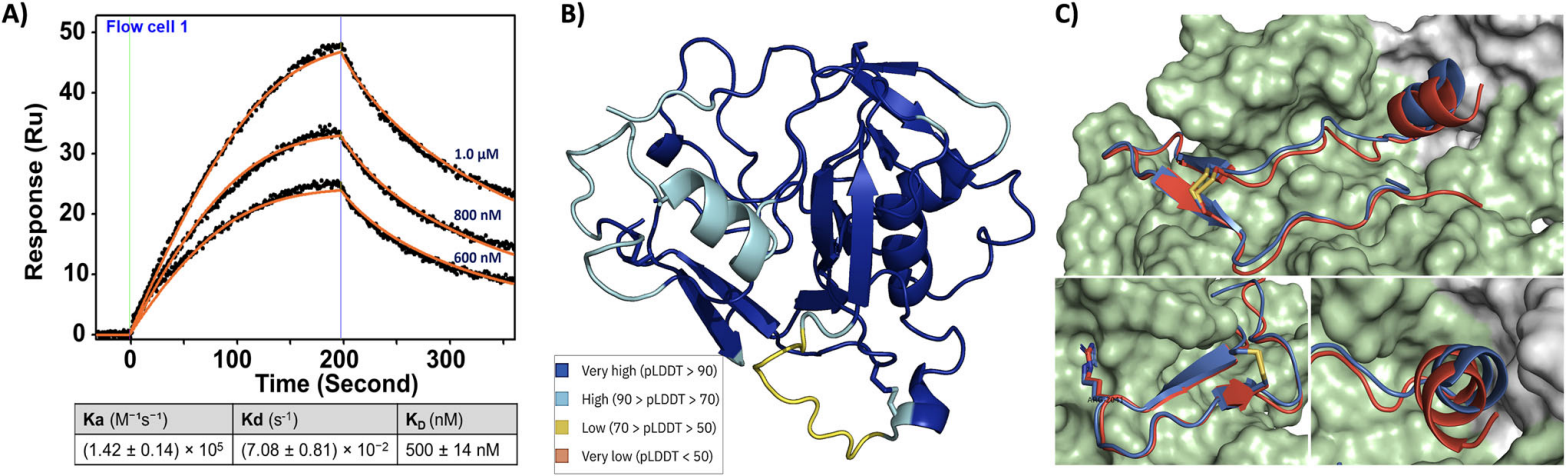
Functional and structural insights into the cyclic*Pf*AMA1‐DI/*Pf*RON2_2021‐2059_ complex. A) Representative sensorgram of AffiTag‐cyclic*Pf*AMA1‐DI (**45**) binding to *Pf*RON2_2021‐2059_ measured by SPR (one of three repeats); the solid orange line represents fitted data. The observed K_D_ (average of three repeats) is 500 ± 14 nM. B) A cartoon representation illustrates the highest‐confidence model of the cyclic*Pf*AMA1‐DI/*Pf*RON2_2021‐2059_ complex, predicted using the ColabFold‐AlphaFold2‐CycPepDock‐Multimer‐v3 protocol without template information (pLDDT = 91.8, pTM = 0.91, and ipTM = 0.892). The model is colored by pLDDT score, reflecting residue‐level confidence. C) Top: Overlay of *Pf*RON2_2021‐2059_ (cartoon representation) from the crystal structure (PDB: 3zwz, blue) with the highest‐confidence predicted model (red) occupying the *Pf*AMA1 hydrophobic groove. The *Pf*AMA1‐(DI+DII) structure with the DII loop (shown as surface representation), was predicted using Alphafold2‐Multimer‐v3 (pLDDT = 87.4, pTM = 0.867, ipTM = 0.914). Bottom left: The predicted cyclic*Pf*AMA1‐DI model retains key interactions between *Pf*RON2 loop residues, especially Arg2041, similar to those observed in the native crystal structure. Bottom right: N‐terminal helix of *Pf*RON2_2021‐2059_ tilted away from the DII loop region in the predicted structure compared to the *Pf*RON2_2021‐2059_ in crystal structure. Domain I residues are shown in green surface and Domain II in gray.

In the highest‐confidence model, *Pf*RON2_2021‐2059_ occupies the hydrophobic groove of cyclic*Pf*AMA1‐DI similar to its binding orientation in *Pf*AMA1‐(DI+DII). Notably, the N‐terminal α‐helix of *Pf*RON2_2021‐2059_ is slightly shifted away from the DII‐loop region, which is absent in the cyclic*Pf*AMA1‐DI construct (Figure 5C). Our prior reported molecular dynamics simulations^[^ ^28^ ^]^ revealed transient interactions between *Pf*RON2_2021‐2059_ N‐terminal residues and the DII loop in full‐length *Pf*AMA1‐(DI+DII), indicating a dynamic “breathing motion” in the DII loop that transiently interacts with α‐helical regions of *Pf*RON2_2021‐2059_—interactions that static structures typically do not capture. Furthermore, previous studies on a truncated *Pf*RON2_2021‐2059_ variant (*Pf*RON2sp2; *Pf*RON2_2027‐2055_), lacking the N‐terminal α‐helix, demonstrated a binding affinity around 520 nM^[^ ^6a^ ^]^ (SPR) against full‐length *Pf*AMA1‐(DI+DII). This aligns closely with the K_D_ of 500 nM observed in our SPR experiments for engineered cyclic*Pf*AMA1‐DI binding to *Pf*RON2_2021‐2059_.

These results underscore that while domain I alone is capable of initiating *Pf*RON2_2021‐2059_ binding, domain II is essential for maintaining stability of the binary complex. The K_D_ of 500 nM for cyclic*Pf*AMA1‐DI binding to *Pf*RON2_2021‐2059_ suggests that a D‐enantiomeric form of the engineered cyclic*Pf*AMA1‐DI, preserving the conserved hydrophobic *Pf*RON2 binding groove as predicted by ColabFold‐AlphaFold2‐CycPepDock, could act as a miniature D‐protein mimic of *Pf*AMA1. Such a D‐isomer would be ideal for selecting inhibitors from L‐protein libraries via mirror‐image biological display, presenting a promising approach for anti‐malarial therapeutic development.

## 3 Conclusion

This study presents the first successful chemical synthesis of both linear and cyclic forms of the truncated 180‐residue *Pf*AMA1 domain I, a crucial target in antimalarial drug discovery. Through a convergent, multi‐segment native chemical ligation strategy, the synthesis was optimized utilizing one‐pot chemistries and solubility‐enhancing tags, significantly improving the yield and purity. The linear *Pf*AMA1‐DI construct displayed issues with terminal flexibility and incomplete disulfide bond formation, leading to misfolding. To address this, a Gly‐Ser‐Gly (GSG) linker was introduced, enabling backbone cyclization between the N‐ and C‐termini. This cyclization enhanced structural rigidity and facilitated folding forming three disulfide bonds. Despite these improvements, the folding yield of cyclic*Pf*AMA1‐DI was limited by protein aggregation—a known challenge in the refolding of *Pf*AMA1 proteins. Notably, the incorporation of an AffiTag (multipurpose affinity and solubility tag) further boosted folding efficiency and allowed gentle surface immobilization for successful kinetic studies of binding with *Pf*RON2_2021‐2059_.

Kinetic experiments confirmed that the folded cyclic*Pf*AMA1‐DI had the correct disulfide combinations and retained the crucial hydrophobic groove necessary for *Pf*RON2 binding, although, as expected, the binding affinity (K_D_ = 500 nM) was weaker than that of the full‐length *Pf*AMA1‐(DI+DII). This result aligns with prior studies, which indicated that truncated *Pf*AMA1‐DI constructs lacking domain II exhibit faster dissociation rates and reduced complex stability. The absence of domain II in cyclic*Pf*AMA1‐DI thus correlates with a significantly higher dissociation rate, as domain II, particularly DII‐loop, plays a pivotal role in stabilizing the *Pf*AMA1‐*Pf*RON2_2021‐2059_ binary complex by reducing dissociation, thereby prolonging the complex's half‐life.

In summary, this study underscores the effectiveness of backbone cyclization in stabilizing inherently unstable protein domains. The chemically engineered cyclic*Pf*AMA1‐DI construct offers a promising platform for subsequent biophysical characterization and drug discovery efforts. Furthermore, the chemical synthesis route described here opens up new avenues for exploring the chemical synthesis of the D‐enantiomer of the *Pf*AMA1‐DI, potentially leading to the development of mirror‐image therapeutics using various biological display technique. These findings suggest that integrating backbone cyclization with strategic tag incorporation could provide a robust framework for engineering functional miniature proteins.

## Supporting Information

The authors have cited additional references within the Supporting Information.

## Author Contributions

K.M. conceptualized the project. J.M., V.M., A.K., and S.S. performed experiments. J.M., V.M., A.K., and S.S. curated the data. J.M., V.M., A.K., S.S., and K.M. analyzed the data. K.M. acquired funds. K.M. supervised. J.M. and V.M. wrote the original draft. J.M., V.M., and K.M. wrote reviewed and edited the draft with input from all co‐authors.

## Conflict of Interest

The authors declare no conflict of interest.

## Supporting information



Supporting Information

## Data Availability

The data that support the findings of this study are available in the supplementary material of this article.
