# Supplementary material for: Chemical Protein Engineering: Backbone Cyclization Rescues Folding of a 183‐Residue Truncated Domain of Malaria Parasite Protein PfAMA1
Source: Chemistry. 2025 Apr 21;31(28):e202500894. doi: 10.1002/chem.202500894 (PMC12089923; doi:10.1002/chem.202500894)
Supplement: Supplementary file 1 — Supporting Information [file CHEM-31-e202500894-s001.pdf]

Chemical Protein Engineering: Backbone Cyclization Rescues Folding of a 183-Residue Truncated Domain of Malaria Parasite Protein *Pf*AMA1

Jamsad Mannuthodikayil,[†a] Vishal Malik,[†a] Abhisek Kar,[‡a] Sameer Singh[‡a] and Kalyaneswar Mandal[*a]*

a Tata Institute of Fundamental Research Hyderabad
36/p Gopanpally, Hyderabad, Telangana – 500046, India
*E-mail: kmandal@tifrh.res.in

1. General Methods 3
1.1. Reagents 3
1.2. Reverse-phase HPLC and LC-MS analysis 3
1.3. A general protocol for machine-assisted Fmoc-SPPS 3
2. Syntheses of linear *Pf*AMA1-DI segments 4
2.1. Synthesis of Seg-1 His^{123}–Lys^{148}-$^{\alpha}$*CONHNH$_2$* (1) 4
2.2. Synthesis of Seg-2 ^{Acm}Cys^{149}–Phe^{181}-Dbz-Arg$_5$-$^{\alpha}$*CONH$_2$* (2)[16d] 4
2.3. Synthesis of Seg-3 A182C/ Cys^{182}–Leu^{216}-Dbz-Arg$_5$-$^{\alpha}$*CONH$_2$* (3) 5
2.4. Synthesis of Seg-4 ^{Acm}Cys^{217}–Lys^{246}-$^{\alpha}$*CONHNH$_2$* (4) 5
2.5. Synthesis of Seg-5 Cys^{247}–Phe^{274}-$^{\alpha}$*CONHNH$_2$* (5) 6
2.6. Synthesis of Seg-6 Cys^{275}–Cys^{302}-$^{\alpha}$*COOH* (6)[14d] 6
3. Total chemical synthesis of linear *Pf*AMA1-DI 7
3.1. Native chemical ligation of ^{Acm}Cys^{149}-Phe^{181}-Dbz-Arg$_5$-$^{\alpha}$*CONH$_2$* (2) and Cys^{182}-Leu^{216}- Dbz-Arg$_5$-$^{\alpha}$*CONH$_2$* (3) 7
3.2. Native chemical ligation of His^{123}-Lys^{181}-$^{\alpha}$*CONHNH$_2$* (1) and Cys^{149}-Leu^{216}-Dbz-Arg$_5$-$^{\alpha}$*CONH$_2$* (9) 8
3.3. Native chemical ligation of ^{Acm}Cys^{217}-Lys^{246}-$^{\alpha}$*CONHNH$_2$* (4) and Cys^{247}-Phe^{274}-$^{\alpha}$*CONHNH$_2$* (5) 9
3.4. Native chemical ligation of ^{Acm}Cys^{217}-Phe^{274}- $^{\alpha}$*CONHNH$_2$* (11) and Cys^{275}-Cys^{302}- $^{\alpha}$*COOH* (6) 9
3.5. Acm-removal of ^{Acm}Cys^{217}-Cys^{302}-$^{\alpha}$*COOH* (12) 10
3.6. Native chemical ligation of His^{123}-Leu^{216}-Dbz-Arg$_5$-$^{\alpha}$*CONH$_2$* (10) and Cys^{217}-Cys^{302}-$^{\alpha}$*COOH* (13) 11
3.7. Folding of linear *Pf*AMA1-DI polypeptide His^{123}-Cys^{302}-$^{\alpha}$*COOH* (14) 11
4. Syntheses of cyclic *Pf*AMA1-DI segments 12
4.1. Synthesis of Seg-1 *Fmoc*-Cys-Gly-Ser-Gly-His^{123}–Lys^{148}-$^{\alpha}$*COSR* (15") 12
4.2. Synthesis of Seg-2 ^{Acm}Cys^{149}–Phe^{181}-Dbz-Arg$_4$-$^{\alpha}$*CONH$_2$* (16) 13
4.3. Synthesis of Seg-3 A182C/ Cys^{182}–Leu^{216}-Dbz-Arg$_4$-$^{\alpha}$*CONH$_2$* (17) 13
4.4. Synthesis of Seg-4 *Fmoc*-Cys^{217}–Lys^{246}-$^{\alpha}$*COSR* (18") 14
4.5. Synthesis of Seg-5 *Fmoc*-Cys^{247}–Phe^{274}-$^{\alpha}$*COSR* (19") 15
4.6. Synthesis of Seg-6 V301A/ Cys^{275}–Ala^{301}-$^{\alpha}$*CONHNH$_2$* (20) 15
5. Total chemical synthesis of cyclic *Pf*AMA1-DI 16
5.1. Native chemical ligation of ^{Acm}Cys^{149}-Phe^{181}-Dbz-Arg$_4$-$^{\alpha}$*CONH$_2$* (16) and Cys^{182}-Leu^{216}- Dbz-Arg$_4$-$^{\alpha}$*CONH$_2$* (17) 16
5.2. Native chemical ligation of *Fmoc*-Cys-Gly-Ser-Gly-His^{123}-Lys^{181}- $^{\alpha}$*COSR* (15") and Cys^{149}-Leu^{216}-Dbz-Arg$_4$-$^{\alpha}$*CONH$_2$* (24) 17
5.3. One-pot Native chemical ligation of *Fmoc*-Cys^{217}-Lys^{246}-$^{\alpha}$*COSR* (18"), *Fmoc*-Cys^{247}-Phe^{274}- $^{\alpha}$*COSR* (19") and Cys^{275}-Ala^{301}-$^{\alpha}$*CONHNH$_2$* (20) 18
5.4. Native chemical ligation of *Fmoc*-Cys-Gly-Ser-Gly-His^{123}-Leu^{216}-Dbz-Arg$_4$-$^{\alpha}$*CONH$_2$* (25) and Cys^{217}-Ala^{301}-$^{\alpha}$*CONHNH$_2$*(29) 19
5.5. Cyclization and folding of Cys-Gly-Ser-Gly-His^{123}-Ala^{301}-$^{\alpha}$*CONHNH$_2$* (31) 19
6. Cyclic*Pf*AMA1-DI with a multipurpose AffiTag segments synthesis 20
6.1. Synthesis of Seg-6 V301A/ Thz^{275}–Cys^{301}-$^{\alpha}$*CONHNH$_2$* (34) 20
6.2. Synthesis of multipurpose Affitag (35) 21
7. Total chemical synthesis of cyclic*Pf*AMA1-DI with a multipurpose AffiTag 22
7.1. Alkylation of Seg-6 V301A/ Thz^{275}–Cys^{301}-$^{\alpha}$*CONHNH$_2$* (34) with the multipurpose AffiTag (35) 22
7.2. One-pot native chemical ligation of *Fmoc*-Cys^{217}-Lys^{246}-$^{\alpha}$*COSR* (18"), *Fmoc*-Cys^{247}-Phe^{274}- $^{\alpha}$*COSR* (19"), and Cys^{275}-Cys^{301}(AffiTag)-$^{\alpha}$*CONHNH$_2$* (37) 22
7.3. Native chemical ligation of *Fmoc*-Cys-Gly-Ser-Gly-His^{123}-Leu^{216}-Dbz-Arg$_4$-$^{\alpha}$*CONH$_2$* (25) and Cys^{217}-Cys^{301}(AffiTag)-$^{\alpha}$*CONHNH$_2$* (41) 23
7.4. Cyclization and folding of Seg-1+2+3+4+5+6(AffiTag)- $^{\alpha}$*CONHNH$_2$* (43) 24
8. Methionine oxide reduction to methionine (*Pf*AMA1 Seg-5 *Fmoc*-Cys^{247}–Phe^{274}-$^{\alpha}$*CONHNH$_2$* (19)) 25
9. Evaluation of binding activity by surface plasmon resonance (SPR) 25
9.1. Solid phase synthesis of *Pf*RON2$_{2021\text{-}2059}$ peptide 25
9.2. SPR binding of AffiTagged cyclic *Pf*AMA1-DI and *Pf*RON2$_{2021\text{-}2059}$ 26
10. Web-based prediction of cyclic*Pf*AMA1-DI/ *Pf*RON2$_{2021\text{-}2059}$ complex 27

1. General Methods

1.1. Reagents

N,N-Diisopropylethylamine (DIEA), Tris(2-carboxyethyl)phosphine hydrochloride (TCEP), Guanidine hydrochloride (Gu.HCl), Ethyl cyanohydroxyiminoacetate (Oxyma), 4-mercaptophenylacetic acid (MPAA) and all the N$^{\alpha}$-Fmoc protected amino acids were obtained from Chem-Impex International, USA. The side-chain protecting groups used, Asp(OtBu), Glu(OtBu), Asn(trt), Arg(Pbf), Ser(tBu), Thr(tBu), Tyr(tBu) and Fmoc-(Dmb)Gly-OH. Fmoc-Cys(trt)-OH were purchased from Gyros Protein Technologies. *N,N*-Dimethylformamide (DMF), dichloromethane(DCM), peptide synthesis grade acetonitrile (CH_3CN), diethyl ether, HPLC grade *N,N′*-diisopropylcarbodiimide (DIC), and trifluoroacetic acid (TFA) were purchased from SRL chemicals India. The HPLC grade acetonitrile (CH_3CN) for peptide purification was purchased from Thermofisher Scientific, India. Piperidine was obtained from AVRA chemicals, India. 2-Chlorotrityl chloride (2-Cl-(Trt)-Cl) resin was purchased from Supra Sciences, India. Sodium 2-mercaptoethanesulfonate (MESNa) and all other common reagents were purchased from Sigma-Aldrich and were of the purest grade available.

1.2. Reverse-phase HPLC and LC-MS analysis

Analytical reverse-phase (RP) HPLC was performed on an Agilent HPLC instrument using an Agilent zorbax SB-C3 (5 µm), 4.6×150 mm reverse-phase silica column at a flow rate of 0.9 mL/min using a linear gradient of 10-54% solvent B in solvent A over 22 min or 10-64% solvent B in solvent A over 27 min at 40 °C (solvent A= 0.1% TFA in H_2O; solvent B = 0.08% TFA in acetonitrile). The UV absorbance of the column eluent was monitored at 214 nm wavelength. The peptide masses were measured across the peak by on-line LC-MS using an Agilent 1290 infinity II/6530 Q-TOF LC/MS instrument. The deconvolution of the charge states of the observed mass was carried out using Agilent MassHunter Qualitative Analysis software (version B.07.00), and the deconvoluted mass of the most abundant isotopologue has been reported with an uncertainty of ± 0.01 Da, unless stated otherwise. Calculated masses were based on average isotope composition or based on the most abundant isotopologue mass determined from the isotopic distribution provided by Agilent MassHunter Qualitative Analysis software.

Preparative reverse phase HPLC (RP-HPLC) of crude peptides was performed with a Waters 1525 preparative HPLC system using Waters C4 (5 µm, 300 Å, 10 x 250 mm) or Agilent ZORBAX-SB C3 (5 µm, 80 Å, 9.4 x 250 mm) columns at 40 °C using an appropriate shallow gradient of increasing concentration of solvent B (0.08% TFA in acetonitrile) in solvent A (0.1% TFA in water) at a flow rate of 5 mL/min. Fractions containing the purified target peptide were identified by ESI-MS. Selected pure fractions were then pooled and lyophilized.

1.3. A general protocol for machine-assisted Fmoc-SPPS

All peptides were synthesized using an automated peptide synthesizer (Tribute-UV/IR from Protein Technologies, USA). Fmoc-SPPS was carried out following reported[23] protocol with minor modifications, using amino acids (AA) (0.25 M), DIC (0.25 M) as a coupling reagent and Oxyma (0.25 M) with DIEA (0.025 M) as additives. Cysteine was coupled for 15 min at room temperature followed by 5 min at 50 °C and Arginine was coupled for 20 min at room temperature followed by 5 min at 50 °C. All other amino acid coupling on the 2-2-Cl-(Trt)-resin was performed for 10 min at 50 °C under N_2 atmosphere with vortex mixing. For the synthesis of peptide sequences containing Asp-Gly, which is prone to aspartimide formation during Fmoc-SPPS at elevated temperatures, Fmoc-(Dmb)Gly-OH was used instead of Fmoc-Gly-OH. For the synthesis of C-terminal hydrazide peptide, hydrazine was coupled by adding 10% (vol/vol) hydrazine in DMF and gently agitating for 30 min.[16b] The unreacted functional group on 2-Cl-(Trt)-resin was capped using 5% MeOH (vol/vol) in DMF. The coupling of amino acids on NH_2NH-2-Cl-(Trt)-resin was performed in a peptide synthesizer following the coupling protocol mentioned above. Fmoc deprotection after every coupling cycle was carried out by 20% piperidine treatment at 50 °C. After synthesis, the peptides were cleaved from the resin using TFA (85%), Phenol (5%), TIPS (2.5%), Water (2.5%), DODT (2.5%), and thioanisole (2.5%) as a cleavage cocktail. After cleavage, the TFA was evaporated under N_2 flow inside a well-ventilated fume hood. The cleaved peptide was precipitated and washed

with diethyl ether. Dry crude peptides were either dissolved in 6 M Gu.HCl and loaded directly on to a preparative HPLC column for purification or used for successive reactions without further purification.

2. Syntheses of linear *Pf*AMA1-DI segments

2.1. Synthesis of Seg-1 His^{123}–Lys^{148}-$^{\alpha}$*$CONHNH_2$* (1)

The peptide His-Gly-Ser-Gly-Ile-Arg-Val-Asp-Leu-Gly-Glu-Asp-Ala-Glu-Val-Ala-Gly-Thr-Gln-Tyr-Arg-Leu-Pro-Ser-Gly-Lys-$^{\alpha}$*$CONHNH_2$* (**1**) was synthesized using NH_2NH-2-Cl-(Trt)-resin (scale = 0.3 mmol; substitution = 0.4 mmol/g) by stepwise Fmoc chemistry SPPS in an automated peptide synthesizer at 50 °C (*see* **Section 1.3** for the peptide synthesis protocol). After global deprotection using the TFA cocktail, the crude peptide **1** was precipitated using diethyl ether. Purification of the peptide by preparative HPLC furnished 287 mg (105 µmol, 35%) of the pure peptide segment His^{123}–Lys^{148}-$^{\alpha}$*$CONHNH_2$* (**1**). Observed mass (**ESI-MS**): 2726.40 Da (deconvoluted most abundant isotopologue); calculated mass: 2726.39 Da (most abundant isotopologue) (**Figure S1**).

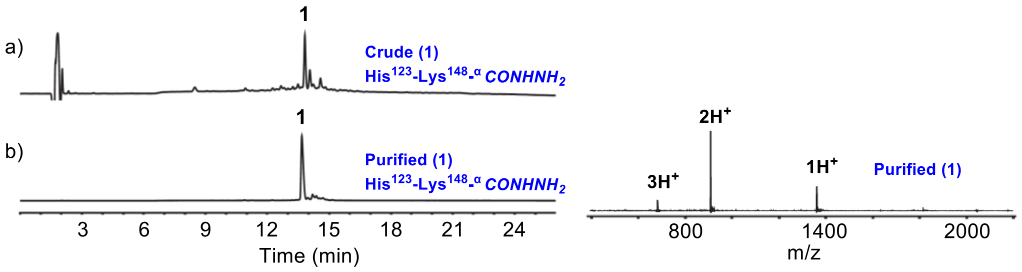

Figure S1. Analytical HPLC profile (λ= 214 nm) together with ESI-MS data (right) of (a) crude peptide His^{123}-Lys^{148}- $^{\alpha}$*$CONHNH_2$* (**1**) (b) purified peptide His^{123}-Lys^{148}- $^{\alpha}$*$CONHNH_2$* (**1**) synthesized using NH_2NH-2-Cl-(Trt)-resin. Linear gradient 10%-54% of B over 22 min including 4 min equilibration using Agilent Zorbax SB-C3 5 µm 4.6 x 150 mm LC column with 0.9 mL/min flow rate was used for the chromatographic separation. Purification was performed using a linear gradient 15%-45% of buffer B in buffer A over 60 min with a flow rate of 5 mL/min at 40 °C (buffer A = 0.1% TFA in water; buffer B = 0.08% TFA in acetonitrile) using a C3, 9.4 x 250 mm column (Agilent ZORBAX-SB C3, 80 Å, 5 µm).

2.2. Synthesis of Seg-2 ^{Acm}Cys^{149}–Phe^{181}-Dbz-Arg$_5$-$^{\alpha}$*$CONH_2$* (2)[16d]

The stepwise synthesis of peptide ^{Acm}Cys-Pro-Val-Phe-Gly-Lys-Gly-Ile-Ile-Ile-Glu-Asn-Ser-Asn-Thr-Thr-Phe-Leu-Thr-Pro-Val-Ala-Thr-Gly-Asn-Gln-Tyr-Leu-Lys-Asp-Gly-Gly-Phe-Dbz-Arg-Arg-Arg-Arg-Arg-$^{\alpha}$*$CONH_2$* (**2**) was carried out on Fmoc-[o-Boc]Dbz-Arg$_5$-Rink-Amide aminomethyl resin in a 0.3 mmol scale by machine-assisted SPPS at elevated temperature (*see* **Section 1.3** for the peptide synthesis protocol). After global deprotection using the TFA cocktail, the crude peptide **2** was precipitated using diethyl ether. Purification of the peptide by preparative HPLC furnished 179.1 mg (39.9 µmol, 13.3%) of the pure peptide segment ^{Acm}Cys^{149}–Phe^{181}-Dbz-Arg$_5$-$^{\alpha}$*$CONH_2$* (**2**). Observed mass (**ESI-MS**): 4487.91 Da (deconvoluted most abundant isotopologue); calculated mass: 4488.87 Da (most abundant isotopologue) (**Figure S2**).

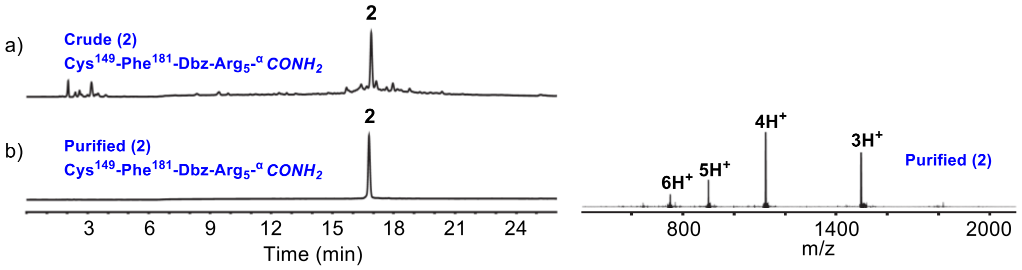

Figure S2. Analytical HPLC profile (λ= 214 nm) together with ESI-MS data (right) of (a) crude peptide ^{Acm}Cys^{149}-Phe^{181}-Dbz-Arg$_5$- $^{\alpha}$*$CONH_2$* (**2**) (b) purified peptide ^{Acm}Cys^{149}-Phe^{181}-Dbz-Arg$_5$- $^{\alpha}$*$CONH_2$* (**2**). Linear gradient 10%-54% of B over 22 min including 4 min equilibration using Agilent Zorbax SB-C3 5 µm 4.6 x 150 mm LC column with 0.9 mL/min flow rate was used for the chromatographic separation. Purification was performed using a linear gradient 15%-

50% of buffer B in buffer A over 70 min with a flow rate of 5 mL/min at 40 °C (buffer A = 0.1% TFA in water; buffer B = 0.08% TFA in acetonitrile) using a C3, 9.4 x 250 mm column (Agilent ZORBAX-SB C3, 80 Å, 5 µm).

2.3. Synthesis of Seg-3 A182C/ Cys^{182}–Leu^{216}-Dbz-Arg$_5$-$^{\alpha}$*CONH$_2$* (3)

The peptide Cys-Phe-Pro-Pro-Thr-Glu-Pro-Leu-Met-Ser-Pro-Met-Thr-Leu-Asp-Glu-Met-Arg-His-Phe-Tyr-Lys-Asp-Asn-Lys-Tyr-Val-Lys-Asn-Leu-Asp-Glu-Leu-Thr-Leu-Dbz-Arg-Arg-Arg-Arg-Arg-$^{\alpha}$*CONH$_2$* (**3**) was synthesized using Fmoc-[o-Boc]Dbz-Arg$_5$-Rink-Amide aminomethyl resin (scale = 0.3 mmol; substitution = 0.5 mmol/g) by stepwise Fmoc chemistry SPPS in an automated peptide synthesizer at 50 °C (*see* **Section 1.3** for the peptide synthesis protocol). After global deprotection using the TFA cocktail, the crude peptide **3** was precipitated using diethyl ether. Purification of the peptide by preparative HPLC furnished 109.3 mg (21.3 µmol, 7.1%) of the pure peptide segment Cys^{182}-Leu^{216}-Dbz-Arg$_5$-$^{\alpha}$*CONH$_2$* (**3**). Observed mass (**ESI-MS**): 5131.64 Da (deconvoluted most abundant isotopologue); calculated mass: 5132.57 Da (most abundant isotopologue) (**Figure S3**).

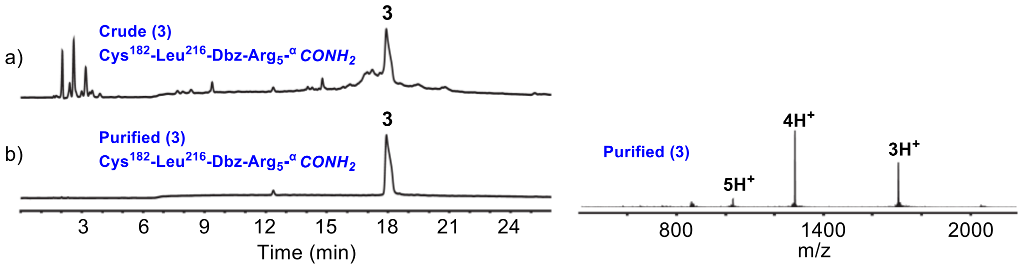

Figure S3. Analytical HPLC profile (λ= 214 nm) together with ESI-MS data (right) of (a) crude peptide Cys^{182}-Leu^{216}-Dbz-Arg$_5$-$^{\alpha}$*CONH$_2$* (**3**) (b) purified peptide Cys^{182}-Leu^{216}-Dbz-Arg$_5$-$^{\alpha}$*CONH$_2$* (**3**). Linear gradient 10%-54% of B over 22 min including 4 min equilibration using Agilent Zorbax SB-C3 5 µm 4.6 x 150 mm LC column with 0.9 mL/min flow rate was used for the chromatographic separation. Purification was performed using a linear gradient 20%-50% of buffer B in buffer A over 60 min with a flow rate of 5 mL/min at 40 °C (buffer A = 0.1% TFA in water; buffer B = 0.08% TFA in acetonitrile) using a C3, 9.4 x 250 mm column (Agilent ZORBAX-SB C3, 80 Å, 5 µm).

2.4. Synthesis of Seg-4 ^{Acm}Cys^{217}–Lys^{246}-$^{\alpha}$*CONHNH$_2$* (4)

The peptide ^{Acm}Cys-Ser-Arg-His-Ala-Gly-Asn-Met-Ile-Pro-Asp-Asn-Asp-Lys-Asn-Ser-Asn-Tyr-Lys-Tyr-Pro-Ala-Val-Tyr-Asp-Asp-Lys-Asp-Lys-Lys-$^{\alpha}$*CONHNH$_2$* (**4**) was synthesized using NH_2NH-2-Cl-(Trt)-resin (scale = 0.3 mmol; substitution = 0.6 mmol/g) by stepwise Fmoc chemistry SPPS in an automated peptide synthesizer at 50 °C (*see* **Section 1.3** for the peptide synthesis protocol). After global deprotection using the TFA cocktail, the crude peptide **4** was precipitated using diethyl ether. Purification of the peptide by preparative HPLC furnished 185.9 mg (52 µmol, 17.3%) of the pure peptide segment ^{Acm}Cys^{217}-Lys^{246}-$^{\alpha}$*CONHNH$_2$* (**4**). Observed mass (**ESI-MS**): 3572.68 Da (deconvoluted most abundant isotopologue); calculated mass: 3572.68 Da (most abundant isotopologue) (**Figure S4**).

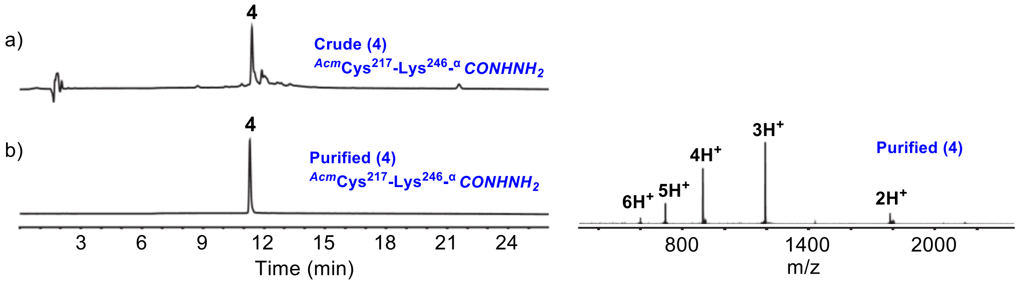

Figure S4. Analytical HPLC profile (λ= 214 nm) together with ESI-MS data (right) of (a) crude peptide ^{Acm}Cys^{217}-Lys^{246}-$^{\alpha}$*CONHNH$_2$* (**4**) (b) purified peptide ^{Acm}Cys^{217}-Lys^{246}-$^{\alpha}$*CONHNH$_2$* (**4**). Linear gradient 10%-54% of B over 22 min including 4 min equilibration using Agilent Zorbax SB-C3 5 µm, 4.6 x 150 mm, LC column with 0.9 mL/min flow rate was used for the chromatographic separation. Purification was performed using a linear gradient 10%-40% of buffer

B in buffer A over 60 min with a flow rate of 5 mL/min at 40 °C (buffer A = 0.1% TFA in water; buffer B = 0.08% TFA in acetonitrile) using a C3, 9.4 x 250 mm column (Agilent ZORBAX-SB C3, 80 Å, 5 µm).

2.5. Synthesis of Seg-5 Cys^{247}–Phe^{274}-$^{\alpha}$*CONHNH$_2$* (5)

The peptide Cys-His-Ile-Leu-Tyr-Ile-Ala-Ala-Gln-Glu-Asn-Asn-Gly-Pro-Arg-Tyr-Cys-Asn-Lys-Asp-Glu-Ser-Lys-Arg-Asn-Ser-Met-Phe-$^{\alpha}$*CONHNH$_2$* (**5**) was synthesized using NH_2NH-2-Cl-(Trt)-resin (scale = 0.3 mmol; substitution = 0.5 mmol/g) by stepwise Fmoc chemistry SPPS in an automated peptide synthesizer at 50°C (*see* **Section 1.3** for the peptide synthesis protocol). After global deprotection using the TFA cocktail, the crude peptide **5** was precipitated using diethyl ether. Purification of the crude peptide by preparative HPLC furnished 218.1 mg (65.8 µmol, 22%) of the pure peptide segment Cys^{247}-Phe^{274}-$^{\alpha}$*CONHNH$_2$* (**5**). Observed mass (**ESI-MS**): 3316.55 Da (deconvoluted most abundant isotopologue); calculated mass: 3316.55 Da (most abundant isotopologue) (**Figure S5**).

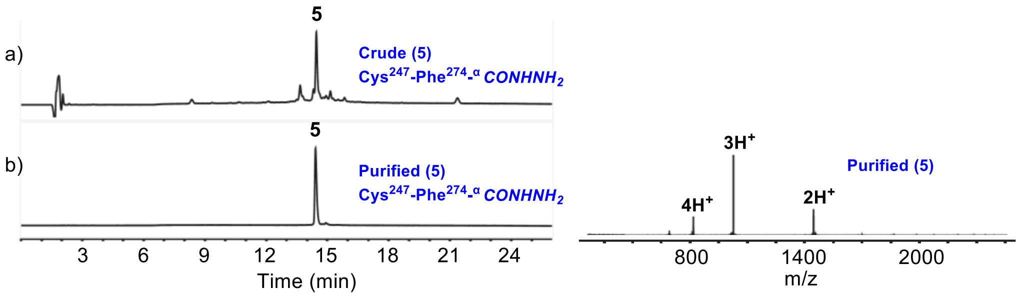

Figure S5. Analytical HPLC profile (λ= 214 nm) together with ESI-MS data (right) of (a) crude peptide Cys^{247}-Phe^{274}- $^{\alpha}$*CONHNH$_2$* (**5**) (b) purified peptide Cys^{247}-Phe^{274}- $^{\alpha}$*CONHNH$_2$* (**5**). Linear gradient 10%-54% of B over 22 min including 4 min equilibration using Agilent Zorbax SB-C3 5 µm 4.6 x 150 mm LC column with 0.9 mL/min flow rate was used for the chromatographic separation. Purification was performed using a linear gradient 10%-40% of buffer B in buffer A over 60 min with a flow rate of 5 mL/min at 40 °C (buffer A = 0.1% TFA in water; buffer B = 0.08% TFA in acetonitrile) using a C3, 9.4 x 250 mm column (Agilent ZORBAX-SB C3, 80 Å, 5 µm).

2.6. Synthesis of Seg-6 Cys^{275}–Cys^{302}-$^{\alpha}$*COOH* (6)[14d]

The peptide Cys-Phe-Arg-Pro-Ala-Lys-Asp-Ile-Ser-Phe-Gln-Asn-Tyr-Thr-Tyr-Leu-Ser-Lys-Asn-Val-Val-Asp-Asn-Trp-Glu-Lys-Val-Cys-$^{\alpha}$*COOH* (**6**) was synthesized on 2-Cl-(Trt)-resin (substitution= 0.4 mmol/g) by stepwise Fmoc chemistry SPPS on a 0.3 mmol scale in an automated peptide synthesizer at 50°C (*see* **Section 1.3** for the peptide synthesis protocol). After global deprotection using the TFA cocktail, the crude peptide **6** was precipitated using diethyl ether. Purification of the peptide by preparative HPLC furnished 160.7 mg (47.7 µmol, 16%) of the pure peptide segment Cys^{275}-Cys^{302}- $^{\alpha}$*COOH* (**6**). Observed mass (**ESI-MS**): 3368.64 Da (deconvoluted most abundant isotopologue); calculated mass: 3368.62 Da (most abundant isotopologue) (**Figure S6**).

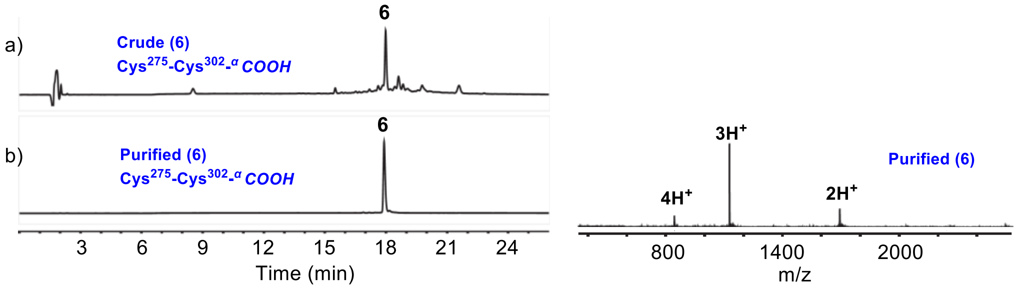

Figure S6. Analytical HPLC profile (λ= 214 nm) together with ESI-MS data (right) of (a) crude peptide Cys^{275}-Cys^{302}- $^{\alpha}$*COOH* (**6**) (b) purified peptide Cys^{275}-Cys^{302}- $^{\alpha}$*COOH* (**6**). Linear gradient 10%-54% of B over 22 min including 4 min equilibration using Agilent Zorbax SB-C3 5 µm 4.6 x 150 mm LC column with 0.9 mL/min flow rate was used for the chromatographic separation. Purification was performed using a linear gradient 15%-50% of buffer B in buffer A

over 70 min with a flow rate of 5 mL/min at 40 °C (buffer A = 0.1% TFA in water; buffer B = 0.08% TFA in acetonitrile) using a C3, 9.4 x 250 mm column (Agilent ZORBAX-SB C3, 80 Å, 5 µm).

3. Total chemical synthesis of linear *Pf*AMA1-DI

3.1. Native chemical ligation of ^{Acm}Cys^{149}-Phe^{181}-Dbz-Arg$_5$-$^{\alpha}$*CONH$_2$* (2) and Cys^{182}-Leu^{216}- Dbz-Arg$_5$-$^{\alpha}$*CONH$_2$* (3)

Peptide ^{Acm}Cys^{149}-Phe^{181}-Dbz-Arg$_5$-$^{\alpha}$*CONH$_2$* (**2,** 61.9 mg, 13.79 µmol) was dissolved in 4.5 mL of aqueous phosphate buffer (0.2 M) containing 6 M Gu.HCl at pH 3.0 and kept at -16.5 °C. After 15 min, 0.45 mL aqueous $NaNO_2$ (0.2 M) was added to the solution of peptide **2** and gently agitated at -16.5 °C for 15 min. Then, 4.5 mL of 0.2 M MPAA in a pH 6.3 buffer (200 mM phosphate buffer, 6 M Gu.HCl) was added to the oxidized solution of peptide **2** and the temperature was raised to room temperature. Afterward, 59 mg (11.49 µmol) of peptide segment Cys^{182}-Leu^{216}- Dbz-Arg$_5$-$^{\alpha}$*CONH$_2$* (**3**) was added to the reaction mixture as solid powder and the pH was adjusted to 6.87. The ligation was monitored by analytical HPLC and ESI-MS as shown in **Figure S7a-d**. After the completion of ligation (monitored by HPLC), 0.1 M TCEP was added to the ligation buffer and agitated at pH 7.0 for 60 min. Purification of the reaction mixture using preparative reverse-phase HPLC gave 49.5 mg (5.69 µmol, 49.5% yield) of the pure ligated polypeptide, ^{Acm}Cys^{149}-Leu^{216}-Dbz-Arg$_5$-$^{\alpha}$*CONH$_2$* (**7**). Observed mass (**ESI-MS**): 8687.38 Da (deconvoluted most abundant isotopologue), calculated mass: 8687.39 Da (most abundant isotopologue) (**Figure S7e**).

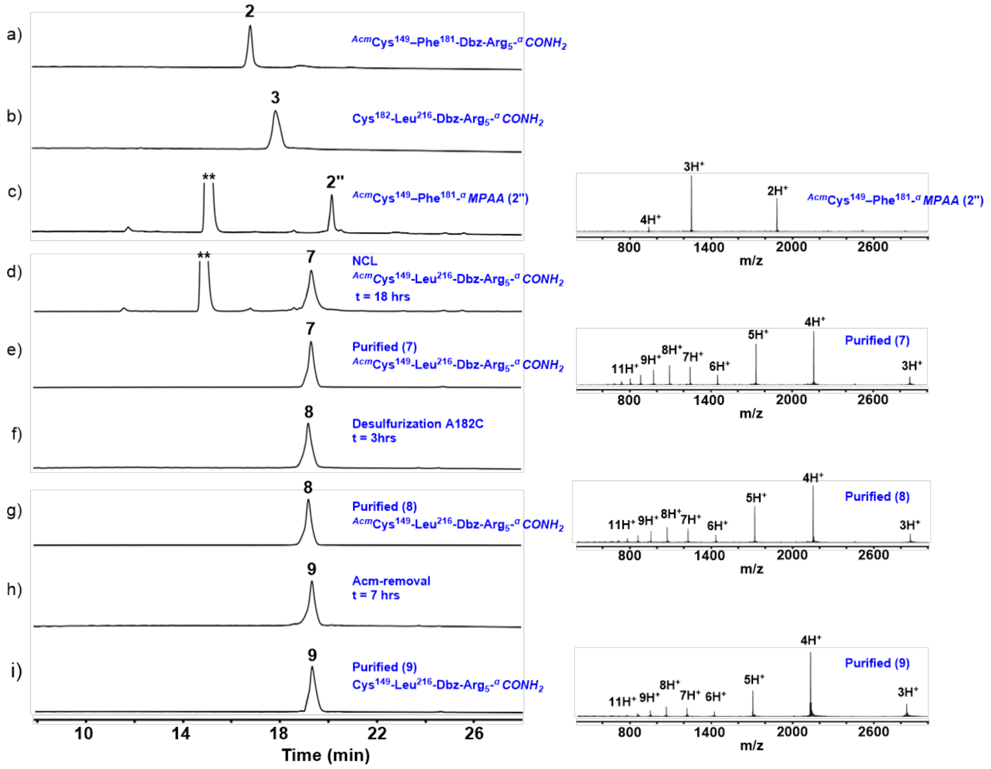

Figure S7. Analytical RP-HPLC profile (λ= 214 nm) together with ESI-MS data (right) of the ligation, desulfurization and Acm-removal reactions. Chromatogram of (a) purified ^{Acm}Cys^{149}-Phe^{181}-Dbz-Arg$_5$-$^{\alpha}$*CONH$_2$* (**2**) (b) purified Cys^{182}-Leu^{216}-Dbz-Arg$_5$-$^{\alpha}$*CONH$_2$* (**3**) (c) ^{Acm}Cys^{149}-Phe^{181}-MPAA (**2"**) after $NaNO_2$ mediated oxidation and MPAA exchange. Peak **2"** is the MPAA exchanged product of peptide **2** (d) the reaction mixture after the addition of peptide Cys^{182}-Leu^{216}- Dbz-Arg$_5$-$^{\alpha}$*CONH$_2$* (**3**) and the ligation was essentially completed within 18 h to give ligated product **7** (e) purified peptide **7**. (f) desulfurized peptide **8** which completed within 3 hours (g) purified desulfurized peptide **8** (h) the reaction mixture after 7 hours of the addition of AgOAc to remove the Acm-group to give peptide **9** (i) purified peptide **9**. Linear gradient 10%-54% of B over 22 min including 4 min equilibration using Agilent Zorbax SB-C3, 5 µm, 4.6 x 150 mm LC column with 0.9 mL/min flow rate was used for the chromatographic separation. Purification was carried out using Agilent zorbax SB-C3 5 µm, 9.4 x 250 mm, LC column using a linear gradient of 25%-55% of buffer B over 30 min for peptides **7**, **8**, and **9** with a flow rate of 5 mL/min at 40 °C. '**' indicates MPAA.

For desulfurization, 75 mM GSH, 100 mM VA-044 and 150 mM TCEP were dissolved in aqueous phosphate buffer (200 mM) containing 6 M Gu.HCl and the pH was adjusted to 7. The dissolved oxygen from the buffer was removed by N_2 gas bubbling. The peptide ^{Acm}Cys^{149}-Leu^{216}- Dbz-Arg$_5$-$^{\alpha}$*CONH$_2$* (**8**) (0.5 mM, 46.9 mg) was then dissolved in the above buffer and incubated at 42 °C. The progress of the reaction

was monitored by ESI-MS analysis, as there was no retention time shift observed between the starting peptide and the product molecule in the gradient used for the LC-MS (**Figure S7e-f**). HPLC purification of the reaction mixture gave 36.78 mg (4.25 µmol, 85% yield) of the desired peptide ^{Acm}Cys^{149}-Leu^{216}-Dbz-Arg$_5$-$^{\alpha}$*CONH$_2$* (**8**). Observed mass (**ESI-MS**): 8655.41 Da (average isotope); calculated mass: 8655.42 Da (**Figure S7e-g**).

Peptide **8** (4.04 µmol, 1.0 equiv, 35 mg) was dissolved in a 50% aq. acetic acid (8.1 ml) containing AgOAc (33.71 mg, 50 equiv, 202 µmol), and the mixture was stirred at 30°C for 7 h. Then 1M DTT (20 mL) in 6M Gu.HCl was added to the mixture, and the formed precipitate was separated by centrifugation. The precipitate was repeatedly washed with 6 M Gu·HCl solution, and the combined supernatant was filtered and purified by preparative HPLC at 25°C with a gradient of 25–55% CH_3CN (with 0.1% TFA) in 30 min to collect the desired fractions and immediately lyophilized, affording the desired protein **9** as a white amorphous powder 21.46 mg (2.5 µmol, 61.0%). The purity and exact mass of the peptide Cys^{149}-Leu^{216}-Dbz-Arg$_5$-$^{\alpha}$*CONH$_2$* (**9**) was confirmed using analytical HPLC and ESI-MS, respectively. Observed mass (**ESI-MS**): 8584.37 Da (deconvoluted most abundant isotopologue), calculated mass: 8584.38 Da (most abundant isotopologue) (**Figure S7g-i**).

3.2. Native chemical ligation of His^{123}-Lys^{181}-$^{\alpha}$*CONHNH$_2$* (1) and Cys^{149}-Leu^{216}-Dbz-Arg$_5$-$^{\alpha}$*CONH$_2$* (9)

Peptide His^{123}-Lys^{181}-$^{\alpha}$*CONHNH$_2$* (**1,** 9.56 mg, 3.51 µmol) was dissolved in 2 mL of aqueous phosphate buffer (0.2 M) containing 6 M Gu.HCl at pH 3.0 and kept at -16.5 °C. After 15 min, 0.2 mL aqueous $NaNO_2$ (0.5 M) was added to the solution of peptide **1** and gently agitated at -16.5 °C for 15 min. Then, 2 mL of 0.2 M MPAA in a pH 5.9 buffer (200 mM phosphate buffer, 6 M Gu.HCl) was added to the oxidized solution of peptide **1** and the temperature was raised to room temperature. Afterward, 24 mg (2.7 µmol) of the second peptide segment Cys^{149}-Leu^{216}-Dbz-Arg$_5$-$^{\alpha}$*CONH$_2$* **(9)** was added to the reaction mixture as solid powder and the pH was adjusted to 6.8. The ligation was monitored by analytical HPLC and ESI-MS as shown in **Figure S8a-d**. After the completion of ligation (monitored by HPLC), 0.1 M TCEP was added to the ligation buffer and agitated at pH 7.0 for 60 min. Purification of the reaction mixture gave 16.58 mg (1.47 µmol, 54% yield) of the pure ligated polypeptide, His^{123}-Leu^{216}-Dbz-Arg$_5$-$^{\alpha}$*CONH$_2$* (**10**). Observed mass **(ESI-MS)**: 11280.08 ± 0.09 Da (average of the six most abundant charge states), calculated mass: 11279.90 Da (average isotope composition) (**Figure S8e**).

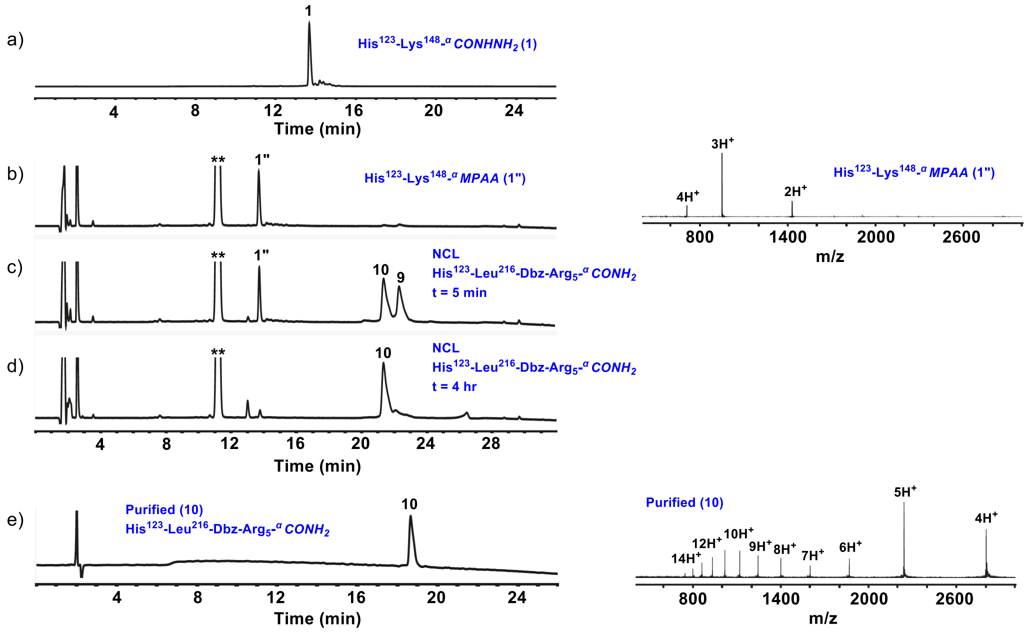

Figure S8. Analytical RP-HPLC profile (λ= 214 nm) together with ESI-MS data (right) of the ligation reaction. Chromatogram of a) purified peptide His^{123}-Lys^{148}-$^{\alpha}$*CONHNH$_2$* (**1**). Linear gradient 10%-54% of B over 22 min including 4 min equilibration using Agilent Zorbax SB-C3, 5 µm, 4.6 x 150 mm LC column with 0.9 mL/min flow rate was used for the chromatographic separation (b) His^{123}-Lys^{148}-$^{\alpha}$*MPAA* after $NaNO_2$ mediated oxidation and MPAA exchange. Peak **1"** is the MPAA exchanged product of peptide **1** (c) reaction at 5 min after the addition of peptide Cys^{149}-Leu^{216}- Dbz-Arg$_5$-$^{\alpha}$*CONH$_2$* (**9**) (d) the reaction mixture after ligation was completed within 4 hours to give ligated product **10**. Linear gradient 15%-75% of B over 30 min including 4 min equilibration using Agilent Zorbax SB-C3, 5 µm, 4.6 x 150 mm LC column with 0.9 mL/min flow rate was used for the chromatographic separation (e) purified ligated product His^{123}-Leu^{216}- Dbz-Arg$_5$-$^{\alpha}$*CONH$_2$* (**10**), eluted at 21.5 min with linear gradient 10%-54% of B over 22 min including 4 min equilibration using Agilent Zorbax SB-C3, 5 µm, 4.6 x 150 mm LC column with 0.9 mL/min flow rate. Purification was carried out using Agilent zorbax SB-

C3 5 µm, 9.4 x 250 mm, LC column using a linear gradient of 15%-45% of buffer B over 60 min with a flow rate of 5 mL/min at 40 °C. The '**' indicates MPAA.

3.3. Native chemical ligation of ^{Acm}Cys^{217}-Lys^{246}-$^{\alpha}$*CONHNH$_2$* (4) and Cys^{247}-Phe^{274}-$^{\alpha}$*CONHNH$_2$* (5)

Peptide ^{Acm}Cys^{217}-Lys^{246}-$^{\alpha}$*CONHNH$_2$* (**4,** 66.1 mg, 18.5 µmol) was dissolved in 5.3 mL of aqueous phosphate buffer (0.2 M) containing 6 M Gu.HCl at pH 3.0 and kept at -16.5 °C. After 15 min, 0.53 mL aqueous $NaNO_2$ (0.5 M) was added to the solution of peptide **4** and gently agitated at -16.5 °C for 15 min. Then, 5.3 mL of 0.2 M MPAA in a pH 6.0 buffer (200 mM phosphate buffer, 6 M Gu.HCl) was added to the oxidized solution of peptide **4** and the temperature was raised to room temperature. Afterward, 51.1 mg (15.4 µmol) of middle peptide segment Cys^{247}-Phe^{274}-$^{\alpha}$*CONHNH$_2$* (**5**) was added to the reaction mixture as solid powder and the pH was adjusted to 6.9. The ligation was monitored by analytical HPLC and ESI-MS as shown in **Figure S9a-c**. After the completion of ligation (monitored by HPLC), 0.1 M TCEP was added to the ligation buffer and agitated at pH 7.0 for 60 min. Purification of the reaction mixture gave 40.9 mg (5.9 µmol, 39% yield) of the pure ligated polypeptide, ^{Acm}Cys^{217}-Phe^{274}-$^{\alpha}$*CONHNH$_2$* (**11**). Observed mass (**ESI-MS**): 6857.20 Da (deconvoluted most abundant isotopologue), calculated mass: 6857.19 Da (most abundant isotopologue) (**Figure S9d**).

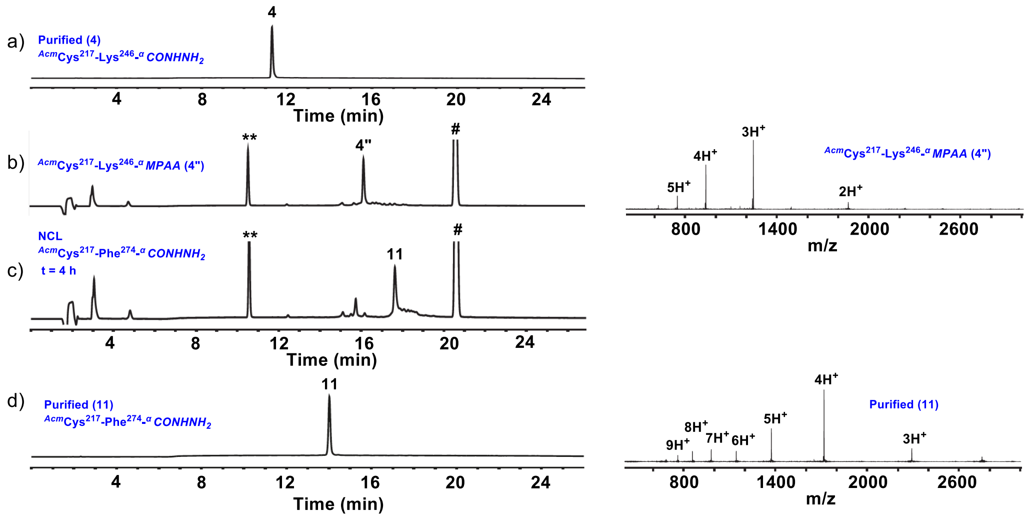

Figure S9. Analytical RP-HPLC profile (λ= 214 nm) together with ESI-MS data (right) of the ligation reaction. Chromatogram of (a) ^{Acm}Cys^{217}-Lys^{246}-$^{\alpha}$*CONHNH$_2$* (**4**). Linear gradient 10%-54% of B over 22 min including 4 min equilibration using Agilent Zorbax SB-C3, 5 µm, 4.6 x 150 mm LC column with 0.9 mL/min flow rate was used for the chromatographic separation (b) ^{Acm}Cys^{217}-Lys^{246}-$^{\alpha}$*MPAA* after $NaNO_2$ mediated oxidation and MPAA exchange. Peak **4"** is the MPAA exchanged product of peptide **4** (c) the reaction mixture at 4 hours after the addition of peptide Cys^{247}-Phe^{274}-$^{\alpha}$*CONHNH$_2$* (**5**). Ligation was essentially completed within 4 hr to give ligated product **11**. Linear gradient 01%-45% of B over 22 min including 3 min equilibration using Agilent Zorbax SB-C3, 5 µm, 4.6 x 150 mm LC column with 0.9 mL/min flow rate was used for the chromatographic separation (d) purified ligated product ^{Acm}Cys^{217}-Phe^{274}-$^{\alpha}$*CONHNH$_2$* (**11**), eluted at 14.0 min with linear gradient 10%-54% of B over 22 min including 4 min equilibration using Agilent Zorbax SB-C3, 5 µm, 4.6 x 150 mm LC column with 0.9 mL/min flow rate. Purification was carried out using Agilent zorbax SB-C3 5 µm, 9.4 x 250 mm, LC column using a linear gradient of 15%-35% of buffer B over 60 min with a flow rate of 5 mL/min at 40 °C. The '**' indicates MPAA.

3.4. Native chemical ligation of ^{Acm}Cys^{217}-Phe^{274}- $^{\alpha}$*CONHNH$_2$* (11) and Cys^{275}-Cys^{302}- $^{\alpha}$*COOH* (6)

Peptide ^{Acm}Cys^{217}-Phe^{274}- $^{\alpha}$*CONHNH$_2$* (**11,** 40.6 mg, 5.9 µmol) was dissolved in 3.5 mL of aqueous phosphate buffer (0.2 M) containing 6 M Gu.HCl at pH 3.0 and kept at -16.5 °C. After 15 min, 0.35 mL of aqueous $NaNO_2$ (0.2 M) solution was added to the solution of peptide **11** and gently agitated for 15 min at -16.5 °C. Then, 3.5 mL of 0.2 M MPAA in pH 5.9 buffer (200 mM PB, 6 M Gu.HCl) was added to the oxidized solution of peptide **11** and the temperature was raised to RT. Afterward, 23.9 mg (7.1 µmol) of the C-terminal peptide segment Cys^{275}-Cys^{302}- $^{\alpha}$*COOH* (**6**) was added to the reaction mixture, and the pH was adjusted to 6.9. The progress of the ligation was monitored by analytical HPLC and ESI-MS as shown in **Figure S10a-c**. Ligation was essentially completed in 5 h to yield the ligated product **12**. After completion of ligation, 0.1 M TCEP was added as solid and agitated at pH 7.0 for 60 min. Purification of the reaction mixture gave 25.8 mg (2.53 µmol, 43% yield) of the desired ligated polypeptide, ^{Acm}Cys^{217}-Cys^{302}-$^{\alpha}$*COOH* (**12**). Observed mass (**ESI-MS**): 10194.20 ± 0.07 Da (average of the eight most abundant charge states), calculated mass: 10194.29 Da (average isotope composition) (**Figure S10d**).

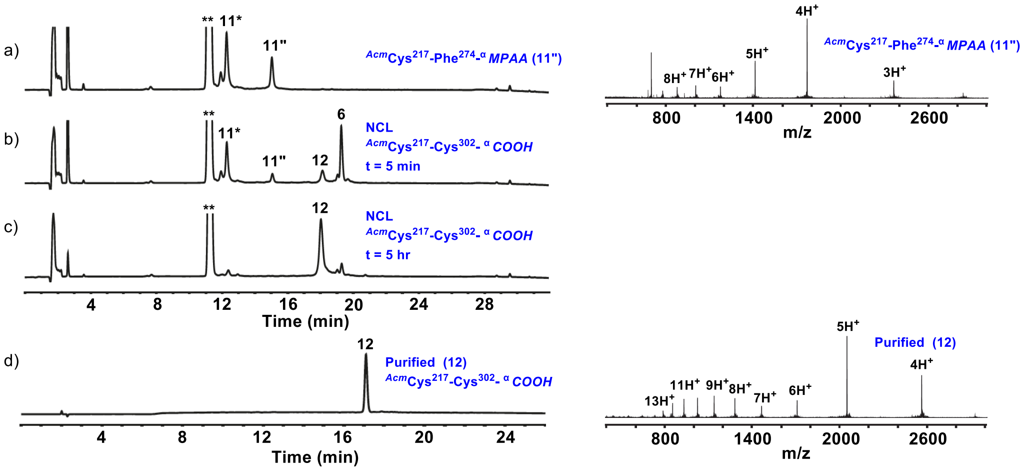

Figure S10. Analytical RP-HPLC profile (λ= 214 nm) together with ESI-MS data (right) of the ligation reaction. Chromatogram of (a) ^{Acm}Cys^{217}-Phe^{274}-${}^{\alpha}$*MPAA* (**11"**) after $NaNO_2$ mediated oxidation and MPAA exchange. A significant quantity of thiolactone (**11***) formation was observed (b) the reaction at 5 min after the addition of peptide Cys^{275}-Cys^{302}-${}^{\alpha}$*COOH* (**6**). (c) Ligation was essentially completed within 5 hours to yield ligated product **15**. Linear gradient 20%-40% of B over 22 min including 5 min equilibration using Agilent Zorbax SB-C3, 5 µm, 4.6 x 150 mm LC column with 0.9 mL/min flow rate was used for the chromatographic separation. (d) Purified ligated product ^{Acm}Cys^{217}-Cys^{302}-${}^{\alpha}$*COOH* (**12**), retention time= 17.0 min, Linear gradient 10%-54% of B over 22 min including 4 min equilibration using Agilent Zorbax SB-C3, 5 µm, 4.6 x 150 mm LC column with 0.9 mL/min flow rate). Purification was carried out on an Agilent zorbax SB-C3 5 µm, 9.4 x 250 mm, LC column using a linear gradient of 20%-40% of buffer B over 60 min with a flow rate of 5 mL/min at 40 °C. The '**' indicates MPAA.

3.5. Acm-removal of ^{Acm}Cys^{217}-Cys^{302}-${}^{\alpha}$*COOH* (12)

For the Acm removal, we followed the procedure described above in *Section-3.1*. In brief, 25.8 mg of *Pf*AMA1-polypeptide ^{Acm}Cys^{217}-Cys^{302}-${}^{\alpha}$*COOH* (**12**, 2.5 µmol, 0.5 mM) was dissolved in 5 mL of 1:1 AcOH/H_2O solution. To this solution was added AgOAc (20 mg, 0.12 mmol, 25 mM) and the reaction mixture was agitated at 30 °C under nitrogen atmosphere. After 5 h, the reaction was quenched by adding 12.5 mL of 1 M DTT in 6 M Gu.HCl. The observed white precipitate was removed via centrifugation. HPLC purification of the supernatant furnished 20.1 mg (1.98 µmol, 79% yield) of the desired polypeptide, Cys^{217}-Cys^{302}-${}^{\alpha}$*COOH* (**13**). Observed mass **(ESI-MS)**: 10123.12 ± 0.07 Da (average of the eight most abundant charge states), calculated mass: 10123.21 Da (average isotope composition) (**Figure S11**).

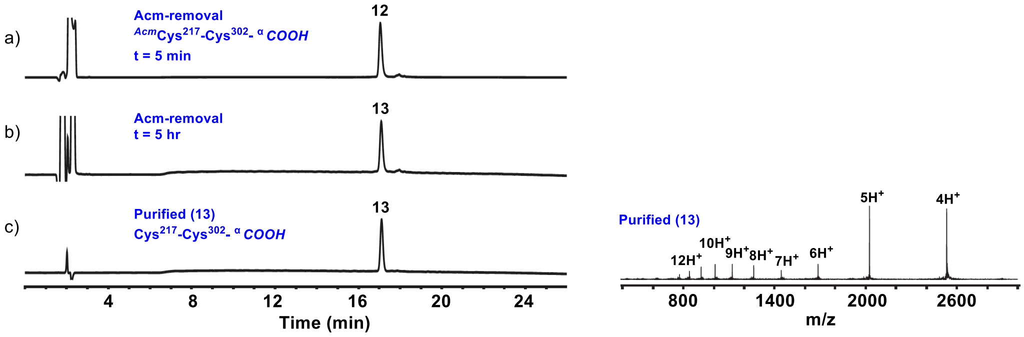

Figure S11. RP-HPLC profile (λ= 214 nm) together with ESI-MS data (right) of the Acm removal of peptide **12.** Chromatogram of (a) polypeptide ^{Acm}Cys^{217}-Cys^{302}-${}^{\alpha}$*COOH* (**12**) at 5 min after the starting of Acm removal reaction. (b) the reaction after 4 hr of Acm group removal reaction. Note that both the peptides are eluted at the same retention time in the gradient used for the separation in analytical HPLC (c) purified product *Pf*AMA1-polypeptide Cys^{217}-Cys^{302}-${}^{\alpha}$*COOH* (**13**). Linear gradient 10%-54% of B over 22 min including 4 min equilibration time using an Agilent Zorbax SB-C3, 5 µm, 4.6 x 150 mm LC column with 0.9 mL/min flow rate was used for the chromatographic separation. Purification was carried out on an Agilent zorbax SB-C3 5 µm, 9.4 x 250 mm, LC column using a linear gradient of 15%-50% of buffer B over 35 min with a flow rate of 5 mL/min at 40 °C.

3.6. Native chemical ligation of His^{123}-Leu^{216}-Dbz-Arg$_5$-$^{\alpha}$*CONH$_2$* (10) and Cys^{217}-Cys^{302}-$^{\alpha}$*COOH* (13)

Peptide His^{123}-Leu^{216}-Dbz-Arg$_5$-$^{\alpha}$*CONH$_2$* (**10,** 5.0 mg, 0.34 µmol) was dissolved in 0.5 mL of aqueous phosphate buffer (0.2 M) containing 6 M Gu.HCl at pH 3.0 and kept at -16.5 °C. After 15 min, 0.05 mL aqueous $NaNO_2$ (0.5 M) was added to the solution of peptide **10** and gently agitated at -16.5 °C for 15 min. Then, 0.5 mL of 0.2 M MPAA in a pH 6.0 buffer (200 mM phosphate buffer, 6 M Gu.HCl) was added to the oxidized solution of peptide **10** and the temperature was raised to room temperature. Afterward, 5.15 mg (0.5 µmol) of the second peptide segment Cys^{217}-Cys^{302}-$^{\alpha}$*COOH* **(13)** was added to the reaction mixture as solid powder and the pH was adjusted to 6.8. The ligation was monitored by analytical HPLC and ESI-MS as shown in **Figure S12a-c**. After the completion of ligation (monitored by HPLC), 0.1 M TCEP was added to the ligation buffer and agitated at pH 7.0 for 60 min. Purification of the reaction mixture gave 4.4 mg (0.22 µmol, 63% yield) of the pure ligated polypeptide, His^{123}-Cys^{302}-$^{\alpha}$*COOH* (**14)**. Observed mass **(ESI-MS)**: 20471.23 ± 0.13 Da (average of the eight most abundant charge states), calculated mass: 20471.06 Da (average isotope composition) (**Figure S12**).

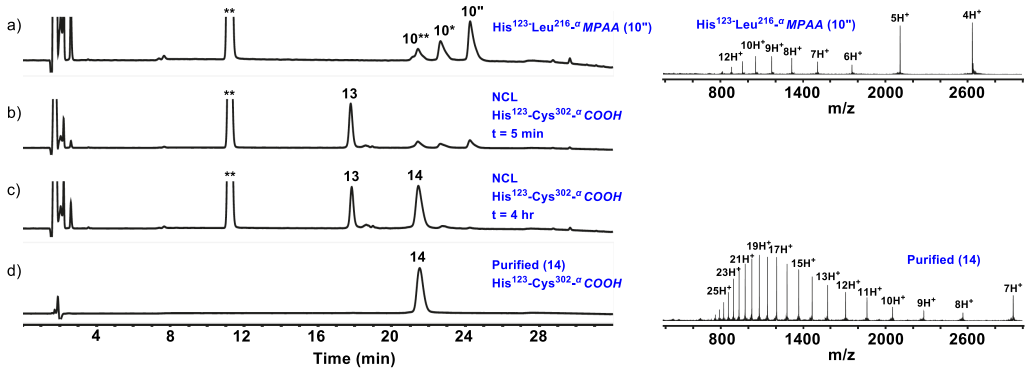

Figure S12. RP-HPLC profile (λ= 214 nm) together with ESI-MS data (right) of the ligation reaction. Chromatogram of (a) His^{123}-Leu^{216}-Dbz-Arg$_5$-$^{\alpha}$*CONH$_2$* (**10"**) after $NaNO_2$ mediated oxidation and MPAA exchange. A significant quantity of thiolactones (**10* and 10****) formation was observed (b) the reaction at 5 min after the addition of peptide Cys^{217}-Cys^{302}- $^{\alpha}$*COOH* (**13**). Ligation was essentially completed within 4 hours to yield ligated product **14** (d) purified ligated product His^{123}-Cys^{302}-$^{\alpha}$*COOH* (**14**), retention time= 21.0 min, Linear gradient 20%-40% of B over 20 min followed by 40%-60% over 5 min including 5 min equilibration using Agilent Zorbax SB-C3, 5 µm, 4.6 x 150 mm LC column with 0.9 mL/min flow rate. Purification was carried out on an Agilent zorbax SB-C3 5 µm, 9.4 x 250 mm, LC column using a linear gradient of 25%-45% of buffer B over 60 min with a flow rate of 5 mL/min at 40 °C. The '**' indicates MPAA.

3.7. Folding of linear *Pf*AMA1-DI polypeptide His^{123}-Cys^{302}-$^{\alpha}$*COOH* (14)

After the successful linear *Pf*AMA1-DI polypeptide (His^{123}–Cys^{302}–αCOOH, **14**) synthesis, efforts to fold the polypeptide into its functional structure with three intra-disulfide bonds under various refolding conditions were unsuccessful, yielding predominantly misfolded species (**Figure S13b-d)**. Below is a summary of the three representative unsuccessful folding attempts: **1)** A redox folding reaction was carried out over 72 hours in the presence of 2 mM cysteine and 1 mM cystine at pH 8.2 (20 mM PB, 100 mM NaCl, 0.5 M Gu.HCl, 4 °C to RT), which was started by spontaneously diluting a 6 mg/mL solution of the linear *Pf*AMA1-DI polypeptide (dissolved in 6 M Gu.HCl, 2 mM cysteine, 1 mM cystine, 20 mM PB, 100 mM NaCl, pH 8.2, 4 °C) twelve-fold. The resulting redox folding mixture contained multiple misfolded species eluting between 18.0–19.0 min on RP-HPLC (**Figure S13b)**. Deconvoluted mass spectrometry of eluted peak identified distinct misfolded species with peaks at 20467.54 Da (two intra-disulfide bonds), 20587.42 Da (one intra- and one inter-disulfide involving a cysteine molecule), 20707.00 Da (one intra- and two inter-disulfides with two cysteines), 20827.57 Da (one intra- and three inter-disulfides with three cysteines), and 20946.96 Da (one intra- and four inter-disulfides with four cysteines). 2) Similarly, redox folding in a solution containing 1 mM reduced glutathione (GSH) and 0.1 mM oxidized glutathione (GSSG) under otherwise identical conditions yielded multiple misfolded species eluting in the same 18.0–19.0 min retention window (**Figure S13c)**. Mass spectrometry confirmed peaks at 20467.99 Da (two intra-disulfide bonds), 20773.85 Da (two intra-disulfides and one inter-disulfide with a glutathione molecule), and 21079.61 Da (two intra- and two inter-disulfides with two glutathione molecules). 3) Air oxidation (**Figure S13d)** performed over 72 hours (0.5 mg/mL polypeptide **14**, 20 mM Tris, 100 mM NaCl, pH 7.8, 4 °C to RT), similarly failed to achieve correct folding. The major misfolded product displayed a mass peak at 20466.95 Da, indicative of two intra-disulfide bonds, along with a minor shouldering peak at 20482.94 Da (not marked in mass

spectrum) corresponding to an oxidized variant with one additional oxygen atom. These results underscore the challenges of correctly folding the synthesized linear *Pf*AMA1-DI polypeptide under standard oxidative and redox folding conditions, as all attempts (several attempts using various other conditions not shown) led to aberrant disulfide bond formation and misfolding.

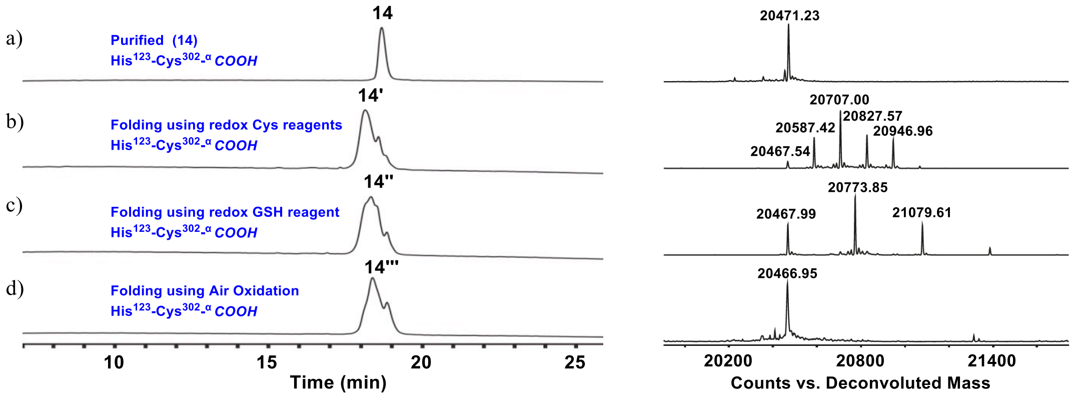

Figure S13. Analytical RP-HPLC chromatograms (λ = 214 nm) and corresponding deconvoluted ESI-MS spectra (right) depicting folding outcomes of the linear polypeptide His^{123}–Cys^{302}–$^{\alpha}$*COOH* **(14)** under various conditions. Chromatographic separation was performed using an Agilent ZORBAX SB-C3 column (5 µm, 4.6 × 150 mm) with a linear gradient of 10–54% solvent B over 22 minutes (including a 4-minute equilibration) at a flow rate of 0.9 mL/min. (a) Chromatogram of purified unfolded polypeptide **14**, eluting at 19.0 minutes, with a single deconvoluted mass peak at 20471.23 Da, corresponding to the expected unfolded polypeptide. (b) Chromatogram of the folding reaction after 72 hours under redox conditions (2 mM cysteine, 1 mM cystine, 0.5 mg/mL polypeptide **14**, 0.5 M Gu.HCl, 20 mM PB, 100 mM NaCl, pH 8.2, 4 °C to RT). Multiple misfolded products were observed, eluting between 18.0 and 19.0 minutes, with deconvoluted mass peaks at 20467.54 Da (two intra-disulfide bonds), 20587.42 Da (one intra- and one inter-disulfide with a cysteine molecule), 20707.00 Da (one intra- and two inter-disulfides with two cysteine molecules), 20827.57 Da (one intra- and three inter-disulfides with three cysteine molecules), and 20946.96 Da (one intra- and four inter-disulfides with four cysteine molecules). (c) Chromatogram of the folding reaction after 72 hours under redox conditions (1 mM GSH, 0.1 mM GSSG, 0.5 mg/mL polypeptide **14**, 0.5 M Gu.HCl, 20 mM PB, 100 mM NaCl, pH 8.2, 4 °C to RT). Misfolded products eluted between 18.0 and 19.0 minutes, with deconvoluted mass peaks at 20467.99 Da (two intra-disulfide bonds), 20773.85 Da (two intra-disulfides and one inter-disulfide with one glutathione molecule), and 21079.61 Da (two intra- and two inter-disulfides with two glutathione molecules). (d) Chromatogram of the folding reaction after 72 hours using air oxidation (0.5 mg/mL polypeptide **14**, 20 mM Tris, 100 mM NaCl, pH 7.8, 4 °C to RT). The major product, eluting between 18.0 and 19.0 minutes, exhibited a deconvoluted mass peak at 20466.95 Da (two intra-disulfide bonds) with a minor shouldering peak at 20482.94 Da (not marked), corresponding to two intra-disulfide bonds and one additional oxygen atom (oxidation).

4. Syntheses of cyclic *Pf*AMA1-DI segments

4.1. Synthesis of Seg-1 *Fmoc*-Cys-Gly-Ser-Gly-His^{123}–Lys^{148}-$^{\alpha}$*COSR* (15")

The corresponding hydrazide peptide *Fmoc*-Cys-Gly-Ser-Gly-His-Gly-Ser-Gly-Ile-Arg-Val-Asp-Leu-Gly-Glu-Asp-Ala-Glu-Val-Ala-Gly-Thr-Gln-Tyr-Arg-Leu-Pro-Ser-Gly-Lys-$^{\alpha}$*CONHNH$_2$* (**15**) was first synthesized using NH_2NH-2-Cl-(Trt)-resin (substitution = 0.4 mmol/g) by stepwise Fmoc chemistry SPPS (0.2 mmol scale) in an automated peptide synthesizer (*see* **Section 1.3** for the peptide synthesis protocol). After global deprotection using TFA cocktail, the peptide hydrazide was precipitated using diethyl ether. The mass of the crude peptide **15** was confirmed by LC-MS (**Figure S14a)**; Observed mass (ESI-MS): 3252.56 Da (deconvoluted most abundant isotopologue); calculated mass: 3252.54 Da (most abundant isotopologue). Crude peptide **15** (~170 mg, 0.052 mmol) was then dissolved in 7 mL of aqueous phosphate buffer (0.2 M) containing 6 M Gu.HCl at pH 3.0 and incubated at -16.5 °C (using Julabo). After 15 min, 700 µL of aqueous $NaNO_2$ (0.5 M) was added to the solution of peptide **15** and gently agitated for 15 min at -16.5 °C. Afterward, 7 mL of 0.2 M MESNa containing 0.2 M aqueous phosphate buffer and 6 M Gu.HCl at pH 6.4 was mixed into the oxidized solution of peptide **15** and the temperature was raised to room temperature. The pH was adjusted to 6.5 and the MESNa exchange was complete within 20 min as monitored by LCMS (**Figure S14b**). Finally, 344 mg of TCEP (100 mM) was added as solid powder and the pH of the resulting reaction mixture was adjusted to 3.5 and agitated for 30 min to reduce the oxidized cysteines in the peptide before purification. Purification using preparative HPLC afforded 54.5 mg (16.2 µmol, 31.1% yield) of the desired MESNa exchanged peptide *Fmoc*-Cys-Gly-Ser-Gly-His^{123}–Lys^{148}-$^{\alpha}$*COSR* **15"** (**Figure S14c**). Observed mass (ESI-MS): 3363.49 Da (deconvoluted most abundant isotopologue); calculated mass: 3363.48 Da (most abundant isotopologue).

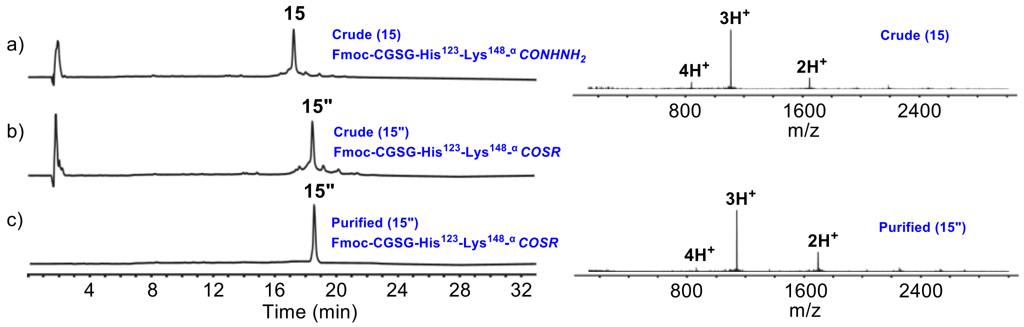

Figure S14. Analytical HPLC profile (λ= 214 nm) together with ESI-MS data (right). Chromatogram of (a) crude peptide *Fmoc*-Cys-Gly-Ser-Gly-His^{123}–Lys^{148}-$^{\alpha}$*CONHNH$_2$* (**15**) (b) *Fmoc*-Cys-Gly-Ser-Gly-His^{123}–Lys^{148}-$^{\alpha}$*COSR* (**15"**) after $NaNO_2$ mediated oxidation and MPAA exchange (c) purified peptide *Fmoc*-Cys-Gly-Ser-Gly-His^{123}–Lys^{148}-$^{\alpha}$*COSR* (**15"**). Linear gradient 10%-64% of B over 27 min including 4 min equilibration using Agilent Zorbax SB-C3 5 µm 4.6 x 150 mm LC column with 0.9 mL/min flow rate was used for the chromatographic separation. Purification was performed using a linear gradient 20%-50% of buffer B in buffer A over 60 min with a flow rate of 5 mL/min at 40 °C (buffer A = 0.1% TFA in water; buffer B = 0.08% TFA in acetonitrile) using an Agilent zorbax SB-C3 5 µm, 9.4 x 250 mm, LC column.

4.2. Synthesis of Seg-2 ^{Acm}Cys^{149}–Phe^{181}-Dbz-Arg$_4$-$^{\alpha}$*CONH$_2$* (16)

The stepwise synthesis of peptide ^{Acm}Cys-Pro-Val-Phe-Gly-Lys-Gly-Ile-Ile-Ile-Glu-Asn-Ser-Asn-Thr-Thr-Phe-Leu-Thr-Pro-Val-Ala-Thr-Gly-Asn-Gln-Tyr-Leu-Lys-Asp-Gly-Gly-Phe-Dbz-Arg-Arg-Arg-Arg-$^{\alpha}$*CONH$_2$* (**16**) was carried out on Fmoc-[o-Boc]Dbz-Arg$_5$-Rink-Amide aminomethyl resin in a 0.3 mmol scale by machine-assisted SPPS at elevated temperature (*see* **Section 1.3** for the peptide synthesis protocol). After global deprotection using the TFA cocktail, the crude peptide **16** was precipitated using diethyl ether. Purification of the peptide by preparative HPLC furnished 204 mg (47.1 µmol, 15.7%) of the pure peptide segment ^{Acm}Cys^{149}–Phe^{181}-Dbz-Arg$_4$-$^{\alpha}$*CONH$_2$* (**16**). Observed mass (**ESI-MS**): 4331.38 Da (deconvoluted most abundant isotopologue); calculated mass: 4331.27 Da (most abundant isotopologue) (**Figure S15**).

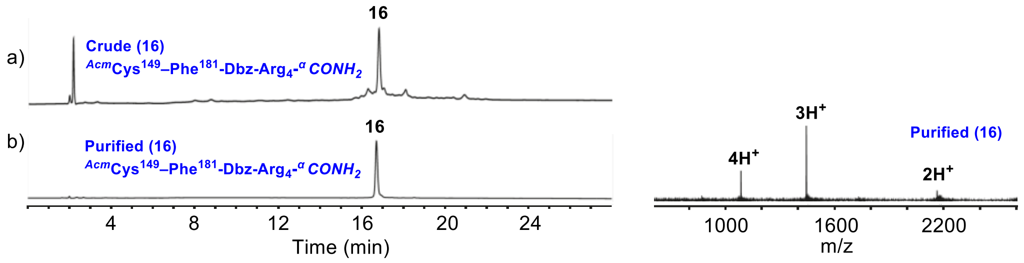

Figure S15. Analytical HPLC profile (λ= 214 nm) together with ESI-MS data (right) of (a) crude peptide ^{Acm}Cys^{149}-Phe^{181}-Dbz-Arg$_4$- $^{\alpha}$*CONH$_2$* (**16**) (b) purified peptide ^{Acm}Cys^{149}-Phe^{181}-Dbz-Arg$_4$- $^{\alpha}$*CONH$_2$* (**16**). Linear gradient 10%-54% of B over 22 min including 4 min equilibration using Agilent Zorbax SB-C3 5 µm 4.6 x 150 mm LC column with 0.9 mL/min flow rate was used for the chromatographic separation. Purification was performed using a linear gradient 15%-50% of buffer B in buffer A over 70 min with a flow rate of 5 mL/min at 40 °C (buffer A = 0.1% TFA in water; buffer B = 0.08% TFA in acetonitrile) using a C3, 9.4 x 250 mm column (Agilent ZORBAX-SB C3, 80 Å, 5 µm).

4.3. Synthesis of Seg-3 A182C/ Cys^{182}–Leu^{216}-Dbz-Arg$_4$-$^{\alpha}$*CONH$_2$* (17)

The peptide Cys-Phe-Pro-Pro-Thr-Glu-Pro-Leu-Met-Ser-Pro-Met-Thr-Leu-Asp-Glu-Met-Arg-His-Phe-Tyr-Lys-Asp-Asn-Lys-Tyr-Val-Lys-Asn-Leu-Asp-Glu-Leu-Thr-Leu-Dbz-Arg-Arg-Arg-Arg-$^{\alpha}$*CONH$_2$* (**17**) was synthesized using Fmoc-[o-Boc]Dbz-Arg$_5$-Rink-Amide aminomethyl resin (scale = 0.3 mmol; substitution = 0.5 mmol/g) by stepwise Fmoc chemistry SPPS in an automated peptide synthesizer at 50 °C (*see* **Section 1.3** for the peptide synthesis protocol). After global deprotection using the TFA cocktail, the crude peptide **17** was precipitated using diethyl ether. Purification of the peptide by preparative HPLC furnished 184 mg (37 µmol, 12.3%) of the pure peptide segment Cys^{182}-Leu^{216}-Dbz-Arg$_4$-$^{\alpha}$*CONH$_2$* (**17**). Observed mass (**ESI-MS**): 4975.62 Da (deconvoluted most abundant isotopologue); calculated mass: 4975.49 Da (most abundant isotopologue) (**Figure S16**).

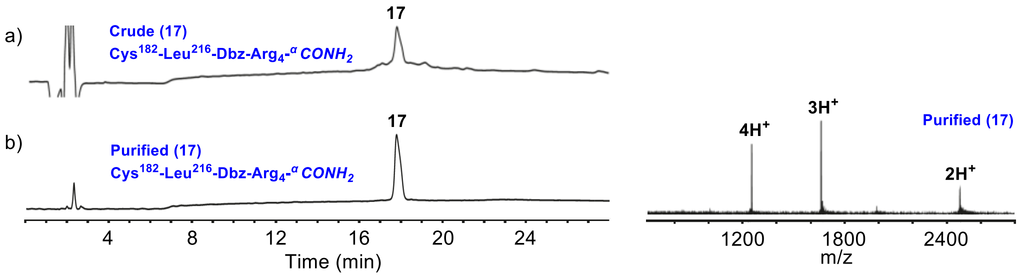

Figure S16. Analytical HPLC profile (λ= 214 nm) together with ESI-MS data (right) of (a) crude peptide Cys^{182}-Leu^{216}-Dbz-Arg$_4$-$^{\alpha}$*CONH$_2$* (17) (b) purified peptide Cys^{182}-Leu^{216}-Dbz-Arg$_4$-$^{\alpha}$*CONH$_2$* (**17**). Linear gradient 10%-54% of B over 22 min including 4 min equilibration using Agilent Zorbax SB-C3 5 µm 4.6 x 150 mm LC column with 0.9 mL/min flow rate was used for the chromatographic separation. Purification was performed using a linear gradient 20%-50% of buffer B in buffer A over 60 min with a flow rate of 5 mL/min at 40 °C (buffer A = 0.1% TFA in water; buffer B = 0.08% TFA in acetonitrile) using a C3, 9.4 x 250 mm column (Agilent ZORBAX-SB C3, 80 Å, 5 µm).

4.4. Synthesis of Seg-4 *Fmoc*-Cys^{217}–Lys^{246}-$^{\alpha}$*COSR* (18")

The corresponding hydrazide peptide *Fmoc*-Cys-Ser-Arg-His-Ala-Gly-Asn-Met-Ile-Pro-Asp-Asn-Asp-Lys-Asn-Ser-Asn-Tyr-Lys-Tyr-Pro-Ala-Val-Tyr-Asp-Asp-Lys-Asp-Lys-Lys-$^{\alpha}$*CONHNH$_2$* (**18**) was first synthesized using NH_2NH-2-Cl-(Trt)-resin (substitution = 0.5 mmol/g) by stepwise Fmoc chemistry SPPS (0.2 mmol scale) in an automated peptide synthesizer (*see* **Section 1.3** for the peptide synthesis protocol). After global deprotection using TFA cocktail, the peptide hydrazide was precipitated using diethyl ether. The mass of the crude peptide **18** was confirmed by LC-MS (**Figure S17a)**; Observed mass (ESI-MS): 3723.73 Da (deconvoluted most abundant isotopologue); calculated mass: 3723.71 Da (most abundant isotopologue). Crude peptide **18 (**185 mg, ~0.05 mmol) was then dissolved in 8 mL of aqueous phosphate buffer (0.2 M) containing 6 M Gu.HCl at pH 3.0 and incubated at -16.5 °C (using Julabo). After 15 min, 0.8 mL of aqueous $NaNO_2$ (0.5 M) was added to the solution of peptide **18** and gently agitated for 15 min at -16.5 °C. Afterward, 8 mL of 0.2 M MESNa containing 0.2 M aqueous phosphate buffer and 6 M Gu.HCl at pH 6.4 was mixed into the oxidized solution of peptide **18** and the temperature was raised to room temperature. The pH was adjusted to 6.5 and the MESNa exchange was complete within 20 min as monitored by LCMS (**Figure S17b)**. Finally, 487 mg of TCEP (~100 mM) was added as solid powder and the pH of the resulting reaction mixture was adjusted to 3.5 and agitated for 30 min to reduce the oxidized cysteines in the peptide before purification. Purification using preparative HPLC afforded 51 mg (13.3 µmol, 26.6% yield) of the desired MESNa exchanged peptide *Fmoc*-Cys^{217}–Lys^{246}-$^{\alpha}$*COSR* **18"** (**Figure S17c**). Observed mass (ESI-MS): 3833.66 Da (deconvoluted most abundant isotopologue); calculated mass: 3833.64 Da (most abundant isotopologue).

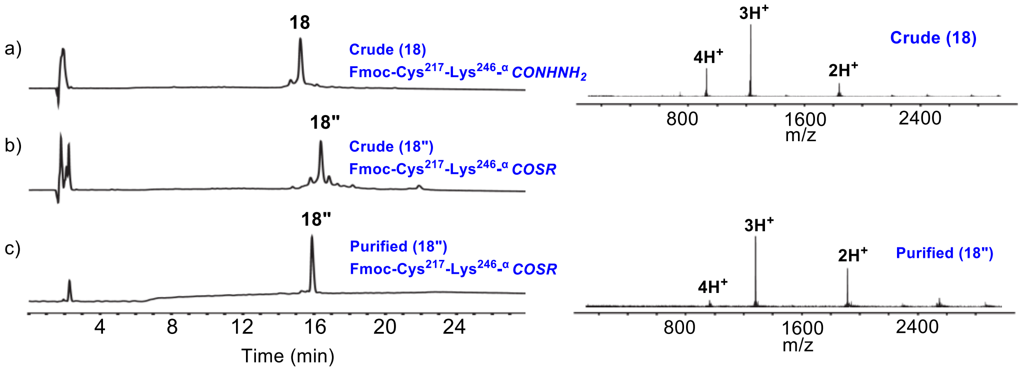

Figure S17. Analytical HPLC profile (λ= 214 nm) together with ESI-MS data (right). Chromatogram of (a) crude peptide *Fmoc*-Cys^{217}–Lys^{246}-$^{\alpha}$*CONHNH$_2$* (**18**) (b) *Fmoc*-Cys^{217}–Lys^{246}-$^{\alpha}$*COSR* (**18"**) after $NaNO_2$ mediated oxidation and MPAA exchange (c) purified peptide *Fmoc*-Cys^{217}–Lys^{246}-$^{\alpha}$*COSR* (**18"**). Linear gradient 10%-64% of B over 27 min including 4 min equilibration using Agilent Zorbax SB-C3 5 µm 4.6 x 150 mm LC column with 0.9 mL/min flow rate was used for the chromatographic separation. Purification was performed using a linear gradient 15%-45% of buffer B in buffer A over 60 min with a flow rate of 5 mL/min at 40 °C (buffer A = 0.1% TFA in water; buffer B = 0.08% TFA in acetonitrile) using an Agilent zorbax SB-C3 5 µm, 9.4 x 250 mm, LC column.

4.5. Synthesis of Seg-5 *Fmoc*-Cys^{247}–Phe^{274}-$^{\alpha}$*COSR* (19")

The corresponding hydrazide peptide *Fmoc*-Cys-His-Ile-Leu-Tyr-Ile-Ala-Ala-Gln-Glu-Asn-Asn-Gly-Pro-Arg-Tyr-Cys-Asn-Lys-Asp-Glu-Ser-Lys-Arg-Asn-Ser-Met-Phe-$^{\alpha}$*CONHNH$_2$* (**19**) was first synthesized using NH_2NH-2-Cl-(Trt)-resin (substitution = 0.4 mmol/g) by stepwise Fmoc chemistry SPPS (0.2 mmol scale) in an automated peptide synthesizer (see **Section 1.3** for the peptide synthesis protocol). After global deprotection using TFA cocktail, the peptide hydrazide was precipitated using diethyl ether. The mass of the crude peptide **19** was confirmed by LC-MS (**Figure S18a)**; Observed mass (ESI-MS): 3538.65 Da (deconvoluted most abundant isotopologue, monoisotopic); calculated mass: 3538.62 Da (monoisotopic). Crude peptide **19** (~355 mg, ~0.1 mmol) was then dissolved in 10 mL of aqueous phosphate buffer (0.2 M) containing 6 M Gu.HCl at pH 3.0 and incubated at -16.5 °C (using Julabo). After 15 min, 1 mL of aqueous $NaNO_2$ (0.5 M) was added to the solution of peptide **19** and gently agitated for 15 min at -16.5 °C. Afterward, 10 mL of 0.2 M MESNa containing 0.2 M aqueous phosphate buffer and 6 M Gu.HCl at pH 6.3 was mixed into the oxidized solution of peptide **19** and the temperature was raised to room temperature. The pH was adjusted to 6.5 and the MESNa exchange was complete within 20 min as monitored by LCMS (**Figure S18-c)**. Finally, 600 mg of TCEP (~100 mM) was added as solid powder and the pH of the resulting reaction mixture was adjusted to 3.5 and agitated for 30 min to reduce the oxidized cysteines in the peptide before purification. (Note: Incubation at pH higher than 4.5 produced thiolactone (-32 Da from the peptide hydrazide mass); hence, the reaction mixture should be agitated at pH <4 for a longer time in case of incomplete disulfide reduction). Purification using preparative HPLC afforded 70 mg (19.2 µmol, 19.2% yield) of the desired MESNa exchanged peptide *Fmoc*-Cys^{247}–Phe^{274}-$^{\alpha}$*COSR* **19"** (**Figure S18c**). Observed mass (ESI-MS): 3648.59 Da (deconvoluted most abundant isotopologue); calculated mass: 3648.56 Da (most abundant isotopologue).

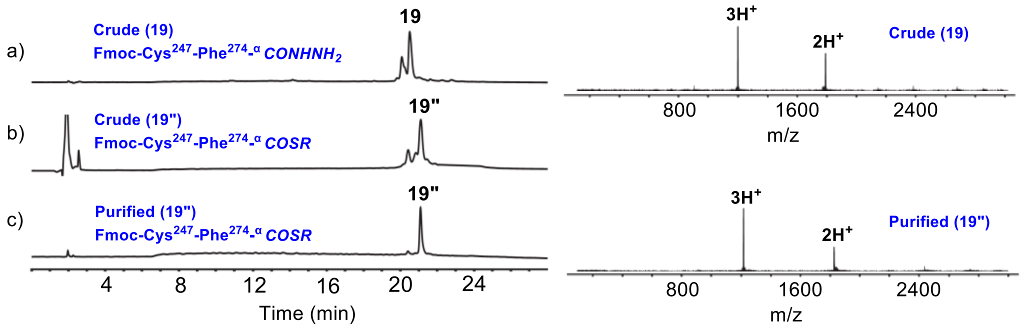

Figure S18. Analytical HPLC profile (λ= 214 nm) together with ESI-MS data (right). Chromatogram of (a) crude peptide *Fmoc*-Cys^{247}–Phe^{274}-$^{\alpha}$*CONHNH$_2$* (**19**) (b) *Fmoc*-Cys^{247}–Phe^{274}-$^{\alpha}$*COSR* (**19"**) after $NaNO_2$ mediated oxidation and MPAA exchange (c) purified peptide *Fmoc*-Cys^{247}–Phe^{274}-$^{\alpha}$*COSR* (**19"**). Linear gradient 10%-64% of B over 27 min including 4 min equilibration using Agilent Zorbax SB-C3 5 µm 4.6 x 150 mm LC column with 0.9 mL/min flow rate was used for the chromatographic separation. Purification was performed using a linear gradient 25%-55% of buffer B in buffer A over 60 min with a flow rate of 5 mL/min at 40 °C (buffer A = 0.1% TFA in water; buffer B = 0.08% TFA in acetonitrile) using an Agilent zorbax SB-C3 5 µm, 9.4 x 250 mm, LC column.

4.6. Synthesis of Seg-6 V301A/ Cys^{275}–Ala^{301}-$^{\alpha}$*CONHNH$_2$* (20)

The peptide Cys-Phe-Arg-Pro-Ala-Lys-Asp-Ile-Ser-Phe-Gln-Asn-Tyr-Thr-Tyr-Leu-Ser-Lys-Asn-Val-Val-Asp-Asn-Trp-Glu-Lys-Ala-$^{\alpha}$*CONHNH$_2$* (**20**) was synthesized using NH_2NH-2-Cl-(Trt)-resin (scale = 0.2 mmol; substitution = 0.6 mmol/g) by stepwise Fmoc chemistry SPPS in an automated peptide synthesizer at 50°C (*see* **Section 1.3** for the peptide synthesis protocol). After global deprotection using the TFA cocktail, the crude peptide (200 mg, 0.061 mmol) **20** was precipitated using diethyl ether. Purification of the peptide by preparative HPLC furnished 58.5 mg (18 µmol, 29%) of the pure peptide segment Cys^{275}–Ala^{301}-$^{\alpha}$*CONHNH$_2$* (**20**). Observed mass (**ESI-MS**): 3250.63 Da (deconvoluted most abundant isotopologue); calculated mass: 3250.60 Da (most abundant isotopologue) (**Figure S19b**).

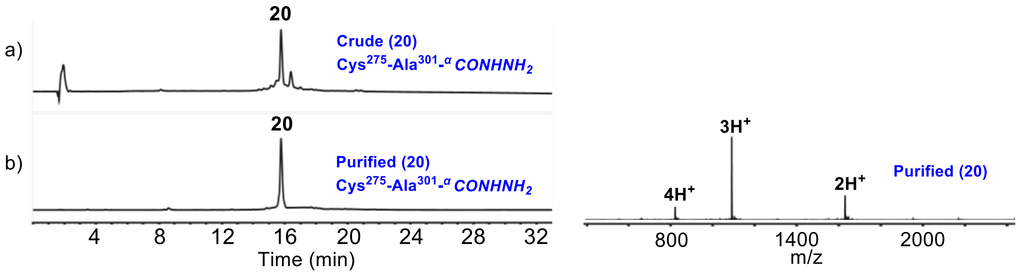

Figure S19. Analytical HPLC profile (λ= 214 nm) together with ESI-MS data (right) of Cys^{275}–Ala^{301}-$^{\alpha}$*CONHNH₂* (**20**). Chromatogram of (a) Crude peptide **20** (b) purified peptide **20**. Linear gradient 10%-64% of B over 22 min including 4 min equilibration using Agilent Zorbax SB-C3 5 µm 4.6 x 150 mm LC column with 0.9 mL/min flow rate was used for the chromatographic separation. Purification was performed using a linear gradient 15%-40% of buffer B in buffer A over 50 min with a flow rate of 5 mL/min at 40 °C (buffer A = 0.1% TFA in water; buffer B = 0.08% TFA in acetonitrile) using an Agilent zorbax SB-C3 5 µm, 9.4 x 250 mm, LC column.

5. Total chemical synthesis of cyclic *Pf*AMA1-DI

5.1. Native chemical ligation of $^{Acm}Cys^{149}$-Phe^{181}-Dbz-Arg_4-$^{\alpha}CONH_2$ (16) and Cys^{182}-Leu^{216}- Dbz-Arg_4-$^{\alpha}CONH_2$ (17)

Peptide $^{Acm}Cys^{149}$-Phe^{181}-Dbz-Arg_4-$^{\alpha}CONH_2$ (**16,** 50 mg, 11.54 µmol) was dissolved in 5 mL of aqueous phosphate buffer (0.2 M) containing 6 M Gu.HCl at pH 3.0 and kept at -16.5 °C. After 15 min, 0.5 mL aqueous $NaNO_2$ (0.2 M) was added to the solution of peptide **16** and gently agitated at -16.5 °C for 15 min. Then, 5 mL of 0.2 M MPAA in a pH 6.5 buffer (200 mM phosphate buffer, 6 M Gu.HCl) was added to the oxidized solution of peptide **16** and the temperature was raised to room temperature. Afterward, 47.8 mg (9.61 µmol) of peptide segment Cys^{182}-Leu^{216}- Dbz-Arg_4-$^{\alpha}CONH_2$ (**17**) was added to the reaction mixture as solid powder and the pH was adjusted to 6.87. The ligation was monitored by analytical HPLC and ESI-MS as shown in **Figure S20a-c**. After the completion of ligation (monitored by HPLC), 0.1 M TCEP was added to the ligation buffer and agitated at pH 7.0 for 60 min. Purification of the reaction mixture using preparative reverse-phase HPLC gave 42.6 mg (5.0 µmol, 52.0% yield) of the pure ligated polypeptide, $^{Acm}Cys^{149}$-Leu^{216}-Dbz-Arg_4-$^{\alpha}CONH_2$ (**22**). Observed mass (**ESI-MS**): 8531.38 Da (deconvoluted most abundant isotopologue), calculated mass: 8531.29 Da (most abundant isotopologue) (**Figure S20d**).

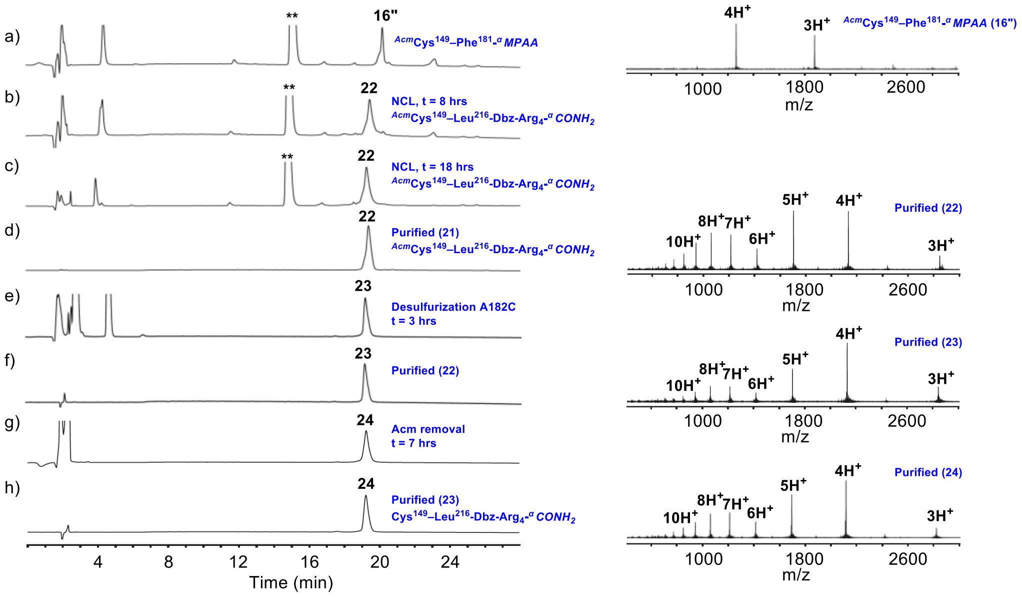

Figure S20. Analytical RP-HPLC profile (λ= 214 nm) together with ESI-MS data (right) of the ligation, desulfurization and Acm-removal reactions. Chromatogram of (a) $^{Acm}Cys^{149}$-Phe^{181}-MPAA (**16"**) after $NaNO_2$ mediated oxidation and MPAA exchange. Peak **16"** is the MPAA exchanged product of peptide **16**. (b) the reaction mixture at t = 8 h after the addition of peptide Cys^{182}-Leu^{216}- Dbz-Arg_4-$^{\alpha}CONH_2$ (**17**) (c) the ligation was essentially completed within 18 h to give ligated product **22**. (d) purified peptide **22**. (e) desulfurized peptide **23** which completed within 3 hours (f) purified desulfurized peptide **23** (g) the reaction mixture after 7 h of the addition of AgOAc to remove the Acm-group to give peptide **24** (h) purified peptide **24**. Linear gradient 10%-54% of B over 22 min including 4 min equilibration using Agilent Zorbax SB-C3, 5 µm, 4.6 x 150 mm LC column with 0.9 mL/min flow rate was used for the

chromatographic separation. Purification was carried out using Agilent zorbax SB-C3 5 µm, 9.4 x 250 mm, LC column using a linear gradient of 25%-55% of buffer B over 30 min for peptides **22**, **23**, and **24** with a flow rate of 5 mL/min at 40 °C. '**' indicates MPAA.

For desulfurization, 75 mM MESNa, 100 mM VA-044 and 150 mM TCEP were dissolved in aqueous phosphate buffer (200 mM) containing 6 M Gu.HCl and the pH was adjusted to 7. The dissolved oxygen from the buffer was removed by N_2 gas bubbling. The peptide ^{Acm}Cys^{149}-Leu^{216}- Dbz-Arg$_4$-$^{\alpha}$*CONH$_2$* (**22**) (0.5 mM, 40.0 mg) was then dissolved in the above buffer and incubated at 42 °C. The progress of the reaction was monitored by ESI-MS analysis, as there was no retention time shift observed between the starting peptide and the product molecule in the gradient used for the LC-MS (**Figure S20d-e**). HPLC purification of the reaction mixture gave 36.78 mg (3.93 µmol, 83.97% yield) of the desired peptide ^{Acm}Cys^{149}-Leu^{216}-Dbz-Arg$_4$-$^{\alpha}$*CONH$_2$* (**23**). Observed mass (**ESI-MS**): 8499.33 Da (average isotope); calculated mass: 8499.31 Da (**Figure S20d-f**).

Peptide **23** (4.11 µmol, 1.0 equiv, 35 mg) was dissolved in a 50% aq. acetic acid (8.0 ml) containing AgOAc (34.30 mg, 50 equiv, 205 µmol), and the mixture was stirred at 30°C for 7 h. Then 1M DTT (20 mL) in 6M Gu.HCl was added to the mixture, and the formed precipitate was separated by centrifugation. The precipitate was repeatedly washed with 6 M Gu·HCl solution, and the combined supernatant was filtered and purified by preparative HPLC at 25°C with a gradient of 25–55% CH_3CN (with 0.1% TFA) in 30 min to collect the desired fractions and immediately lyophilized, affording the desired protein **24** as a white amorphous powder 21.46 mg (2.88 µmol, 70.0%). The purity and exact mass of the peptide Cys^{149}-Leu^{216}-Dbz-Arg$_4$-$^{\alpha}$*CONH$_2$* (**24**) was confirmed using analytical HPLC and ESI-MS, respectively. Observed mass (**ESI-MS**): 8428.29 Da (deconvoluted most abundant isotopologue), calculated mass: 8428.28 Da (most abundant isotopologue) (**Figure S20f-h**).

5.2. Native chemical ligation of *Fmoc*-Cys-Gly-Ser-Gly-His^{123}-Lys^{181}- $^{\alpha}$*COSR* (15") and Cys^{149}-Leu^{216}-Dbz-Arg$_4$-$^{\alpha}$*CONH$_2$* (24)

Peptide *Fmoc*-Cys-Gly-Ser-Gly-His^{123}-Lys^{181}-$^{\alpha}$*COSR* (**15",** 14.78 mg, 4.4 µmol) and Cys^{149}-Leu^{216}-Dbz-Arg$_4$-$^{\alpha}$*CONH$_2$* (**24,** 28.5 mg, 3.38 µmol) was dissolved in 2.0 mL of aqueous phosphate buffer (0.2 M) containing 6 M Gu.HCl along with 50 mM MPAA and 50 mM TCEP at pH 6.9 and kept at RT. The ligation was monitored by analytical HPLC and ESI-MS as shown in **Figure S21a-b**. The ligation was completed in 14 hours. Purification of the reaction mixture gave 19.95 mg (1.71 µmol, 50.4% yield) of the pure ligated polypeptide, Fmoc-Cys-Gly-Ser-Gly-His^{123}-Leu^{216}-Dbz-Arg$_4$-$^{\alpha}$*CONH$_2$* (**25**). Observed mass (**ESI-MS**): 11650.02 ± 0.06 Da (average of the eight most abundant charge states), calculated mass: 11649.91 Da (average isotope composition) (**Figure S21c**).

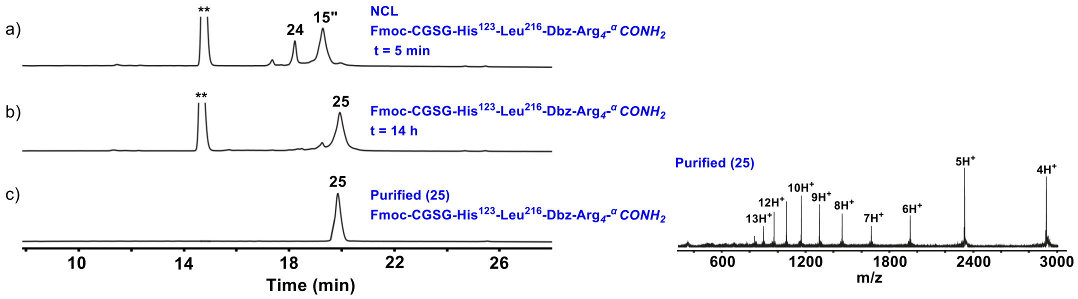

Figure S21. Analytical RP-HPLC profile (λ= 214 nm) together with ESI-MS data (right) of the ligation reaction. Chromatogram of (a) the reaction at 5 min after the addition of peptide *Fmoc*-Cys-Gly-Ser-Gly-His^{123}-Lys^{181}-$^{\alpha}$*COSR* (**15"**) and Cys^{149}-Leu^{216}-Dbz-Arg$_4$-$^{\alpha}$*CONH$_2$* (**24**) in the ligation buffer (b) the reaction mixture after ligation was completed within 14 hours to give ligated product **25** (c) purified ligated product Fmoc-Cys-Gly-Ser-Gly-His^{123}-Leu^{216}-Dbz- Arg$_4$-$^{\alpha}$*CONH$_2$* (**25**). Linear gradient 10%-64% of B over 27 min including 4 min equilibration using Agilent Zorbax SB-C3, 5 µm, 4.6 x 150 mm LC column with 0.9 mL/min flow rate was used for the chromatographic separation. Purification was carried out using Agilent zorbax SB-C3 5 µm, 9.4 x 250 mm, LC column using a linear gradient of 20%-55% of buffer B over 70 min with a flow rate of 5 mL/min at 40 °C. The '**' indicates MPAA.

5.3. One-pot Native chemical ligation of *Fmoc*-Cys^{217}-Lys^{246}-$^{\alpha}$*COSR* (18"), *Fmoc*-Cys^{247}-Phe^{274}- $^{\alpha}$*COSR* (19") and Cys^{275}-Ala^{301}-$^{\alpha}$*CONHNH*$_2$ (20)

For the first ligation, the peptide segment Cys^{275}-Ala^{301}-$^{\alpha}$*CONHNH*$_2$ (**20**, 40 mg, 12.3 µmol) was dissolved in 2.4 mL degassed ligation buffer (200 mM phosphate buffer, 6 M Gu.HCl and 50 mM TCEP) containing 50 mM MPAA, and the buffer pH was adjusted to 6.9. Next, the peptide segment *Fmoc*-Cys^{247}-Phe^{274}- $^{\alpha}$*COSR* (**19"**, 49.36 mg, 13.53 µmol) was added to the reaction mixture as a solid powder. The pH of the reaction mixture was then readjusted to 6.91 and was allowed to react at room temperature to furnish the ligation product *Fmoc*-Cys^{247}-Ala^{301}-COOH (**26**) within 24 h (**Figure S22a-b**). To remove the Fmoc group of the ligated product **26** after the first ligation, ~180 µL concentrated HCl was added followed by ~750 µL piperidine to the reaction mixture to prevent a sudden abrupt change in pH and to fix the final concentration of piperidine to 20% (v/v). The final pH of the reaction mixture was immediately adjusted to 11.0 by using concentrated HCl (12 M) and aqueous NaOH (6 M). The reaction mixture was vortexed vigorously at room temperature. The complete Fmoc deprotection was observed within 7 min (including ~2 min time required for pH adjustment) to afford Cys^{247}-Ala^{302}-$^{\alpha}$*CONHNH*$_2$ (**27**) (**Figure S22b-c**). The pH of the reaction mixture was then rapidly brought down to ~9 by adding concentrated HCl (12 M) followed by the addition of TCEP as a solid powder (final concentration 50 mM). The pH of the reaction mixture was then adjusted to 6.90. For the second ligation, peptide segment *Fmoc*-Cys^{217}-Lys^{246}-$^{\alpha}$*COSR* (**18"**, 56.58 mg, 14.76 µmol) was then added to the reaction mixture and the pH was adjusted back to 6.87. Within 12 h, the peptide **27** was completely converted to the desired ligation product *Fmoc*-Cys^{217}-Ala^{301}-$^{\alpha}$*CONHNH*$_2$ (**28**) (**Figure S22d-e**). To deprotect the Fmoc group from peptide **28**, additional piperidine (to adjust the total concentration to 20% (v/v)) and 6 M NaOH (to increase the pH to 11.0) were added to the reaction mixture. The reaction mixture was then vortexed for a total of 7 min (including ~2 min time required for adjusting pH) to afford the final polypeptide **29** (**Figure S22e-f**). As before, the pH of the reaction buffer was rapidly reduced to ~9.0 by adding concentrated HCl followed by the addition of TCEP (final concentration 50 mM) as solid powder, and the pH was readjusted to 7.0 to achieve complete disulfide reduction. After the two ligations, and two Fmoc deprotection steps, HPLC purification was performed to obtain the pure full-length polypeptide Cys^{217}-Ala^{301}-$^{\alpha}$*CONHNH*$_2$ (**29**, 44.43 mg, 4.44 µmol) with an overall 36% yield. The purity and identity of the *Pf*AMA1 polypeptide **29** were confirmed by LC-MS (**Figure S22g**). Observed mass (**ESI-MS**): 10123.28 ± 0.06 Da (average of the eight most abundant charge states); calculated mass: 10123.21 Da (average isotope composition).

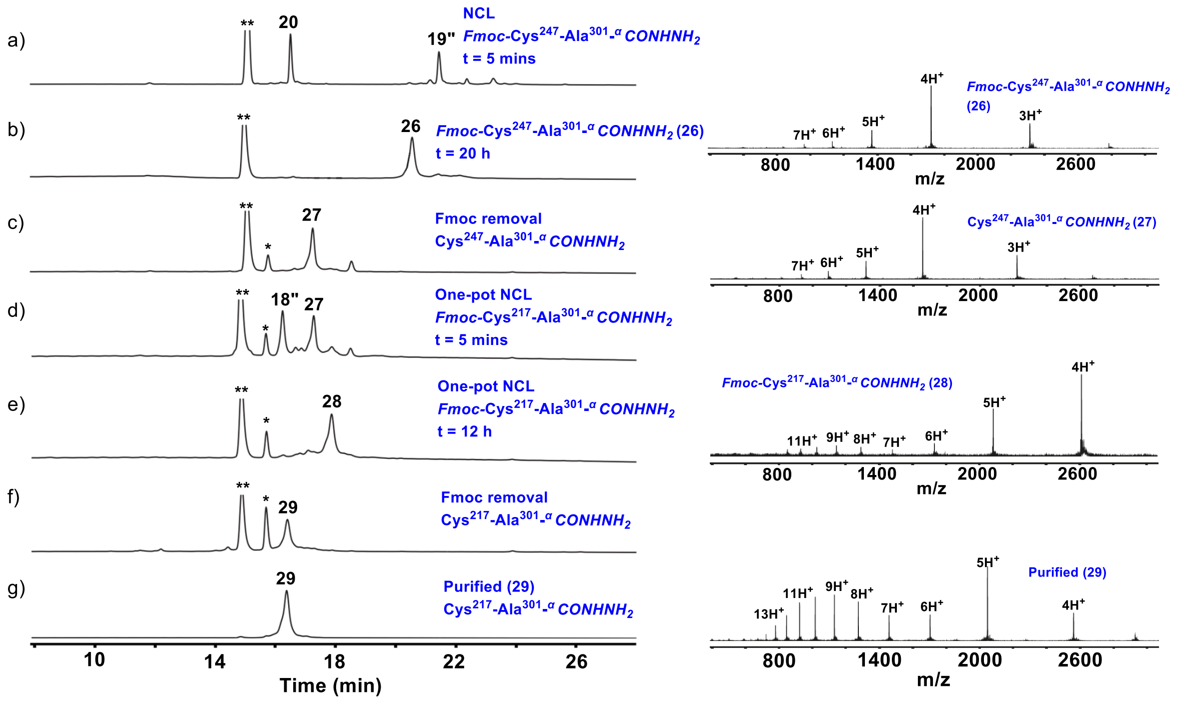

Figure S22. Analytical HPLC monitoring and ESI-MS data (right) of the one-pot ligation reaction: a) 3 min after the addition of the peptide Cys^{275}–Ala^{301}-$^{\alpha}$COOH (**10**) and *Fmoc*-Cys^{247}–Phe^{274}-$^{\alpha}$*COSR* (**19"**) in the standard ligation buffer (200 mM PB, 6 M Gu.HCl, 50 mm TCEP) containing 50 mM MPAA; b) The first ligation, within 20 h, resulted in *Fmoc*-Cys^{247}–Ala^{301}-$^{\alpha}$*COOH* (**26**) as the ligated product; c) Fmoc removal from the ligated peptide **26** to give peptide Cys^{247}–Ala^{301}- $^{\alpha}$*COOH* (**27**); d) 5 min after the addition of peptide *Fmoc*-Cys^{217}–Lys^{246}-$^{\alpha}$*COSR* (**18"**) in the reaction mixture; e) The second ligation was essentially complete within 12 h and furnished polypeptide *Fmoc*-Cys^{217}– Ala^{301}-$^{\alpha}$*COOH* (**28**); f) Fmoc removal from the ligated product **28** to give

target peptide Cys[217]–Ala[301]-$^{\alpha}$*COOH* (**29**). * and ** indicate dibenzofulvene-TECP adduct and MPAA, respectively. Observed mass 10123.28 ± 0.06 Da (average of the eight most abundant charge states) and calculated mass 10 123.21 Da (average isotope composition).

5.4. Native chemical ligation of *Fmoc*-Cys-Gly-Ser-Gly-His[123]-Leu[216]-Dbz-Arg$_4$-$^{\alpha}$*CONH$_2$* (25) and Cys[217]-Ala[301]-$^{\alpha}$*CONHNH$_2$*(29)

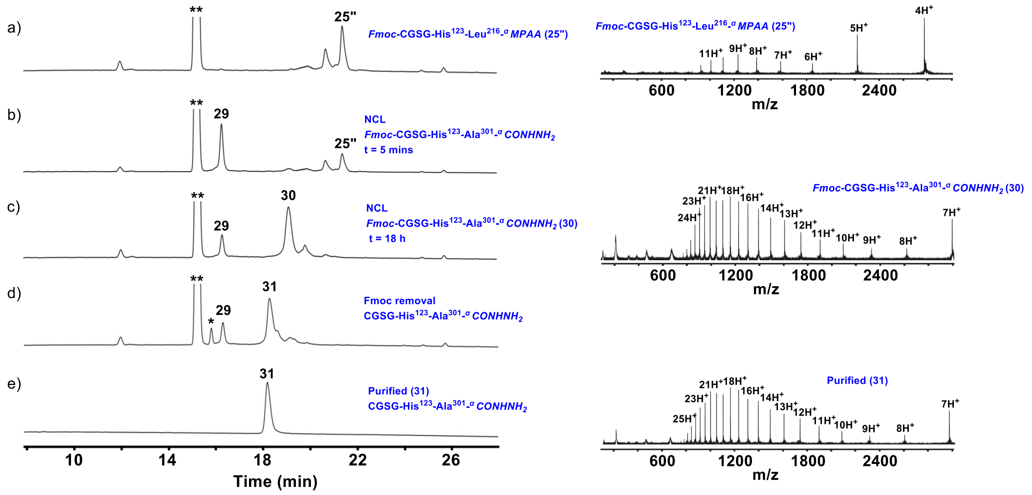

Figure S23. Analytical RP-HPLC profile (λ= 214 nm) together with ESI-MS data (right) of the ligation reaction. Chromatogram of (a) peptide *Fmoc*-Cys-Gly-Ser-Gly-His[123]-Leu[216]-Dbz-Arg$_4$ - $^{\alpha}$*MPAA* (**25"**) after $NaNO_2$ mediated oxidation and MPAA exchange (b) the reaction at 5 min after the addition of the peptide Cys[217]-Ala[301]-$^{\alpha}$*CONHNH$_2$* (**29**) (c) the ligation, within 18 h, resulted in *Fmoc*-Cys-Gly-Ser-Gly-His[123]-Ala[301]-$^{\alpha}$*CONHNH$_2$* (**30**) as the ligated product (d) Fmoc removal from the ligated peptide **30** to give peptide Cys-Gly-Ser-Gly-His[123]-Ala[301]-$^{\alpha}$*CONHNH$_2$* (**31**) (e) purified peptide **31.** Linear gradient 10%-64% of B over 27 min including 4 min equilibration time using an Agilent Zorbax SB-C3, 5 µm, 4.6 x 150 mm LC column with 0.9 mL/min flow rate was used for the chromatographic separation. Purification was carried out using Agilent zorbax SB-C3 5 µm, 9.4 x 250 mm, LC column using a linear gradient of 15%-50% of buffer B over 70 min with a flow rate of 5 mL/min at 40 °C. The '**' indicates MPAA.

Peptide *Fmoc*-Cys-Gly-Ser-Gly-His[123]-Leu[216]-Dbz-Arg$_4$-$^{\alpha}$*CONH$_2$* (**25,** 20 mg, 1.71 µmol) was dissolved in 2 mL of aqueous phosphate buffer (0.2 M) containing 6 M Gu.HCl at pH 3.0 and kept at -16.5 °C. After 15 min, 0.2 mL aqueous $NaNO_2$ (0.5 M) was added to the solution of peptide **25** and gently agitated at -16.5 °C for 15 min. Then, 2 mL of 0.2 M MPAA in a pH 6.5 buffer (200 mM phosphate buffer, 6 M Gu.HCl) was added to the oxidized solution of peptide **25** and the temperature was raised to room temperature. Afterward, 17.11 mg (1.71 µmol) of the second peptide segment Cys[217]-Ala[301]-$^{\alpha}$*CONHNH$_2$* (**29**) was added to the reaction mixture as solid powder and the pH was adjusted to 6.89. The ligation was monitored by analytical HPLC and ESI-MS as shown in **Figure S23a-c**. To deprotect the Fmoc group from peptide **30**, piperidine (to adjust the total concentration to 20% (v/v)) and 6 M NaOH (to increase the pH to 11.0) were added to the reaction mixture. The reaction mixture was then vortexed for a total of 7 min (including ~2 min time required for adjusting pH) to afford the final polypeptide **31**. The pH of the reaction buffer was rapidly reduced to ~9.0 by adding concentrated HCl followed by the addition of 50 mM TCEP as solid powder, and the pH was readjusted to 7.0 to achieve complete disulfide reduction. Purification of the reaction mixture gave 15.9 mg (0.77 µmol, 45% yield) of the pure ligated polypeptide, Cys-Gly-Ser-Gly-His[123]-Ala[301]-$^{\alpha}$*CONHNH$_2$* (**31**). Observed mass **(ESI-MS)**: 20658.20 ± 0.29 Da (average of the eight most abundant charge states), calculated mass: 20658.21 Da (average isotope composition) (**Figure S23e**).

5.5. Cyclization and folding of Cys-Gly-Ser-Gly-His[123]-Ala[301]-$^{\alpha}$*CONHNH$_2$* (31)

Peptide Cys-Gly-Ser-Gly-His[123]-Ala[301]-$^{\alpha}$*CONHNH$_2$* (**31,** 15.3 mg, 0.74 µmol) was dissolved in 15 mL of aqueous phosphate buffer (0.2 M) containing 6 M Gu.HCl at pH 3.0 and kept at -16.5 °C. After 15 min, 1.5 mL aqueous $NaNO_2$ (0.2 M) was added to the solution of peptide **31** and gently agitated at -16.5 °C for 15 min. Then, 7.5 mL of 0.15 M MPAA in a pH 6.2 buffer (200 mM phosphate buffer, 6 M Gu.HCl) was added to the oxidized solution of peptide **31** and the temperature was raised to room temperature. The pH of the reaction buffer was adjusted to 6.52 and kept at RT until the completion of the cyclization. The cyclization was monitored by analytical HPLC and ESI-MS as shown in **Figure S24a-b**. Purification of the reaction mixture gave 7 mg (0.34 µmol, 46% yield) of the pure polypeptide, cyclic(Cys-Gly-

Ser-Gly-His^{123}-Ala^{301}) (**32**). Observed mass (**ESI-MS**): 20626.26 ± 0.13 Da (average of the eight most abundant charge states), calculated mass: 20626.17 Da (average isotope composition) (**Figure S24c**).

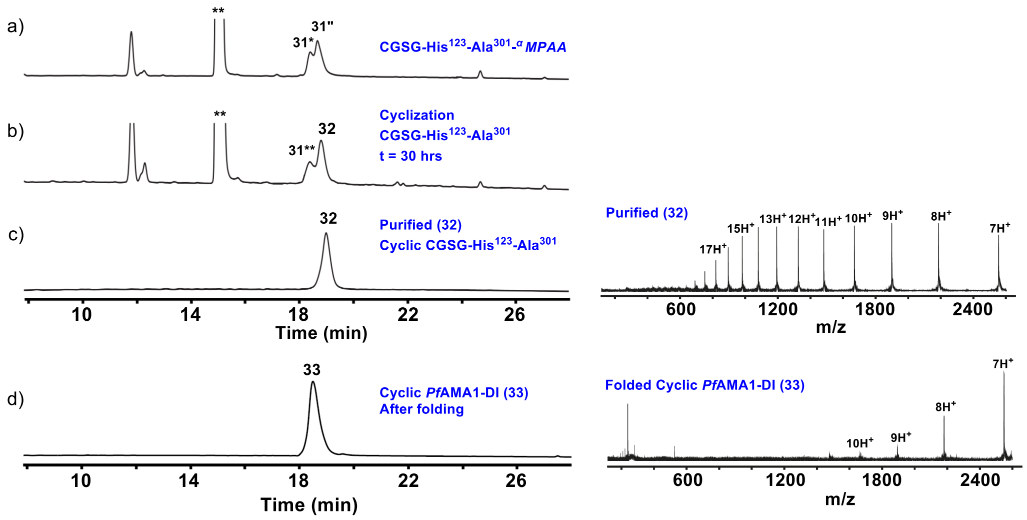

Figure S24. RP-HPLC profile (λ= 214 nm) together with ESI-MS data (right) of the cyclization of peptide **31**. Chromatogram of (a) peptide Cys-Gly-Ser-Gly-His^{123}-Ala^{301}-$^{\alpha}$*MPAA* (**31"**) after $NaNO_2$ mediated oxidation and MPAA exchange. **31*** indicates thiolactone formation from **31** (b) the reaction after 30 h resulting in the formation of cyclized peptide **32. 31**** indicates the thioester hydrolyzed version of peptide **31** (c) purified cyclized peptide **32** (d) folded cyclic *Pf*AMA1-DI (**33**). Linear gradient 10%-64% of B over 27 min including 4 min equilibration time using an Agilent Zorbax SB-C3, 5 µm, 4.6 x 150 mm LC column with 0.9 mL/min flow rate was used for the chromatographic separation. Purification of peptide **32** was carried out using Agilent zorbax SB-C3 5 µm, 9.4 x 250 mm, LC column using a linear gradient of 15%-50% of buffer B over 70 min with a flow rate of 5 mL/min at 40 °C. The '**' indicates MPAA.

Folding: The same folding conditions described in **SI Section 3.7 (Figure S13b–d)** for linear *Pf*AMA1-DI synthesis were investigated for this cyclic analogue. Among those, folding using reduced/oxidized glutathione (1 mM GSH / 0.1 mM GSSG, **Figure S13c**) successfully formed the desired three disulfide bonds. To reduce aggregation and improve the isolated yield of the folded cyclic *Pf*AMA1-DI, the GSH/GSSG folding conditions were further optimized as follows: The purified peptide cyclic(Cys-Gly-Ser-Gly-His^{123}-Ala^{301}) (**32,** 4 mg, 0.193 µmol**)** was dissolved in 0.66 mL buffer (6 M Gu.HCl, 20 mM PB, 1 mM GSH, 0.1 mM GSSG, 100 mM NaCl, pH 8.2) and kept at 4°C for 30 minutes. To this solution, 1.83 mL buffer (20 mM PB, 1 mM GSH, 0.1 mM GSSG, 100 mM NaCl, pH 8.2) was added four times in a 2-hour time interval to reduce the concentration of Gu.HCl from 6 M to 0.5 M and the final volume of the mixture was 8 mL. (*Note*: Buffers were purged with N_2 gas via bubbling prior to the start of the folding procedure). The reaction was monitored for 48 hours, and completion of the folding was confirmed by analytical HPLC and ESI-MS as shown in **Figure S24d** which shows a -6 Da mass difference confirming the formation of 3 disulfide bonds. Observed mass (**ESI-MS**): 20620.19 ± 0.17 Da (average of the four most abundant charge states), calculated mass: 20620.13 Da (average isotope composition) (**Figure S24d**). Precipitates were removed from the reaction mixture by centrifugation (6000 rpm, 5 minutes, 4°C x 3). Remaining 0.5 M Gu.HCl and the redox reagents were removed from the reaction via dialysis (10 KDa membrane) (dialysis buffer: 20 mM phosphate, 100 mM NaCl, pH 7.8, 4°C x 2). The concentration of the folded cyclic(Cys-Gly-Ser-Gly-His^{123}-Ala^{301}) (**33**) was determined by IMPLEN Spectrophotometer (NanoPhotometer® NP80) at 280 nm using the extinction coefficients derived from the protein sequence. The overall folding of the cyclic(Cys-Gly-Ser-Gly-His^{123}-Ala^{301}) peptide gave 0.5 mg (0.024 µmol, 12.5% yield) folded cyclic *Pf*AMA1-DI protein (**33, Figure S24d**).

6. Cyclic*Pf*AMA1-DI with a multipurpose AffiTag segments synthesis

6.1. Synthesis of Seg-6 V301A/ Thz^{275}–Cys^{301}-$^{\alpha}$*CONHNH$_2$* (34)

The peptide Thz-Phe-Arg-Pro-Ala-Lys-Asp-Ile-Ser-Phe-Gln-Asn-Tyr-Thr-Tyr-Leu-Ser-Lys-Asn-Val-Val-Asp-Asn-Trp-Glu-Lys-Cys-$^{\alpha}$*CONHNH$_2$* (**34**) was synthesized using NH_2NH-2-Cl-(Trt)-resin (scale = 0.1 mmol; substitution = 0.5 mmol/g) by stepwise Fmoc chemistry

SPPS in an automated peptide synthesizer at 50 °C (*see* **Section 1.3** for the peptide synthesis protocol). After global deprotection using the TFA cocktail, the crude peptide **34** was precipitated using diethyl ether. Purification of the peptide by preparative HPLC furnished 85 mg (25.8 µmol, 25.8%) of the pure peptide segment Thz^{275}–Cys^{301}-$^{\alpha}$*CONHNH$_2$* (**34**). Observed mass (**ESI-MS**): 3294.60 Da (deconvoluted most abundant isotopologue); calculated mass: 3294.57 Da (most abundant isotopologue) (**Figure S25**).

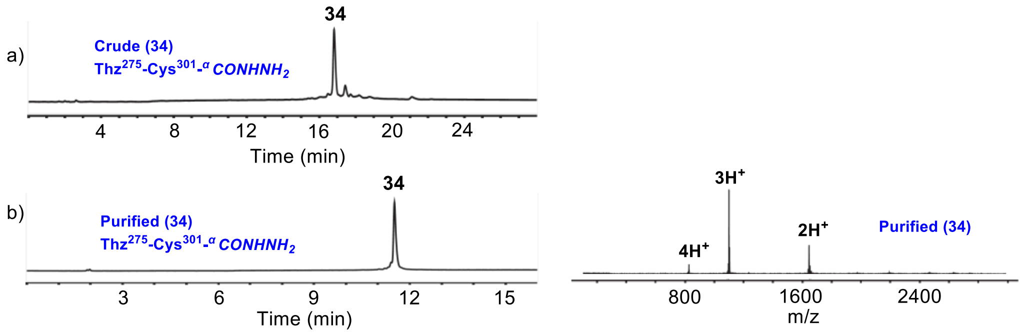

Figure S25. Analytical HPLC profile (λ= 214 nm) together with ESI-MS data (right) of Thz^{275}–Cys^{301}-$^{\alpha}$*CONHNH$_2$* (**34**). Chromatogram of (a) crude peptide Thz^{275}–Cys^{301}-$^{\alpha}$*CONHNH$_2$* (**34**). Linear gradient 10%-64% of B over 27 min including 4 min equilibration using Agilent Zorbax SB-C3 5 µm 4.6 x 150 mm LC column with 0.9 mL/min flow rate was used for the chromatographic separation (b) purified peptide Thz^{275}–Cys^{301}-$^{\alpha}$*CONHNH$_2$* (**34**). Linear gradient 15%-45% of B over 15 min including 4 min equilibration using Agilent Zorbax SB-C3 5 µm 4.6 x 150 mm LC column with 0.9 mL/min flow rate was used for the chromatographic separation. Purification was performed using a linear gradient 15%-45% of buffer B in buffer A over 60 min with a flow rate of 5 mL/min at 40 °C (buffer A = 0.1% TFA in water; buffer B = 0.08% TFA in acetonitrile) using a C3, 9.4 x 250 mm column (Agilent ZORBAX-SB C3, 80 Å, 5 µm).

6.2. Synthesis of multipurpose Affitag (35)

The desired peptide Gly-Ser-Gly-Gly-Ser-Gly-Gly-Ser-Gly-Arg-Arg-Arg-Arg-Gly-Ser-Gly-His-His-His-His-His-His-Gly-Lys-$^{\varepsilon}$Biotin-$^{\alpha}$*CONH$_2$* (**35**) was obtained by machine-assisted SPPS at elevated temperature (*see* **Section 1.3** for the peptide synthesis protocol). Stepwise synthesis of the peptide was carried out on Fmoc deprotected Rink-Amide aminomethyl resin (substitution = 0.6 mmol/g) in a 0.2 mmol scale. On resin, Bromoacetic acid (5 eq) with DIC (4.95 eq) dissolved in DMF was coupled at RT for 15 minutes. After global deprotection using the TFA cocktail, the crude peptide **35** was precipitated using diethyl ether. Purification of the crude peptide (200 mg, 71.4 µmol) by preparative HPLC furnished 75 mg (26.8 µmol, 37.5%) of the pure peptide segment (Gly-Ser-Gly)$_3$-Arg$_4$-Gly-Ser-Gly-His$_6$-Gly-Lys-$^{\varepsilon}$Biotin-$^{\alpha}$*CONH$_2$* (**35**). Observed mass (**ESI-MS**): 2801.22 Da (deconvoluted most abundant isotopologue); calculated mass: 2801.20 Da (most abundant isotopologue) (**Figure S26**).

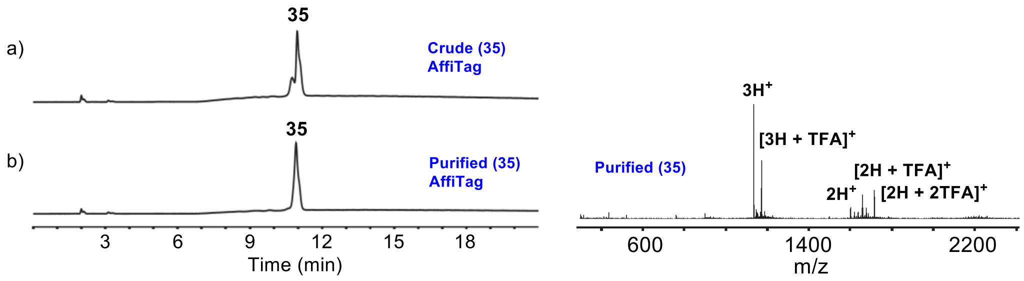

Figure S26. Analytical HPLC profile (λ= 214 nm) together with ESI-MS data (right) of peptide **35**. Chromatogram of (a) crude peptide **35** (b) purified peptide (Gly-Ser-Gly)$_3$-Arg$_4$-Gly-Ser-Gly-His$_6$-Gly-Lys-$^{\varepsilon}$Biotin-$^{\alpha}$*CONH$_2$* (**35**). Linear gradient 05%-45% of B over 20 min including 4 min equilibration using Agilent Zorbax SB-C3 5 µm 4.6 x 150 mm LC column with 0.9 mL/min flow rate was used for the chromatographic separation. Purification was performed using a linear gradient 01%-31% of buffer B in buffer A over 60 min with a flow rate of 5 mL/min at 40 °C (buffer A = 0.1% TFA in water; buffer B = 0.08% TFA in acetonitrile) using a C18, 10 x 250 mm column (Phenomenex proteo, 90 Å, 4 µm).

7. Total chemical synthesis of cyclic*Pf*AMA1-DI with a multipurpose AffiTag

7.1. Alkylation of Seg-6 V301A/ Thz^{275}–Cys^{301}-$^{\alpha}$*CONHNH$_2$* (34) with the multipurpose AffiTag (35)

Peptide Thz^{275}-Cys^{301}-$^{\alpha}$*CONHNH$_2$* (**34,** 71 mg, 21.5 µmol) was dissolved in 5.0 mL of aqueous phosphate buffer (0.2 M) containing 6 M Gu.HCl at pH 8.4 and kept at RT. To this solution, peptide AffiTag (**35,** 73 mg, 25.8 µmol) was added as solid and gently agitated for 1 hr. The reaction was monitored by analytical HPLC and ESI-MS as shown in **Figure S27a-b**. Observed mass of peptide **36 (ESI-MS)**: 6015.89 Da (deconvoluted most abundant isotopologue), calculated mass: 6015.85 Da (most abundant isotopologue) (**Figure S27b**). To deprotect Thz group in the same reaction mixture, 0.1 M CH_3ONH_2·HCl was added to the ligation buffer and pH was adjusted to 4.5. The reaction was agitated for 2 h at RT. Purification of the reaction mixture gave 87.0 mg (14.5 µmol, 67.4% yield) of the pure ligated polypeptide, Cys^{275}-Cys^{301}(AffiTag)-$^{\alpha}$*CONHNH$_2$* (**37**). Observed mass **(ESI-MS)**: 6003.88 Da (deconvoluted most abundant isotopologue), calculated mass: 6003.85 Da (most abundant isotopologue) (**Figure S27d**).

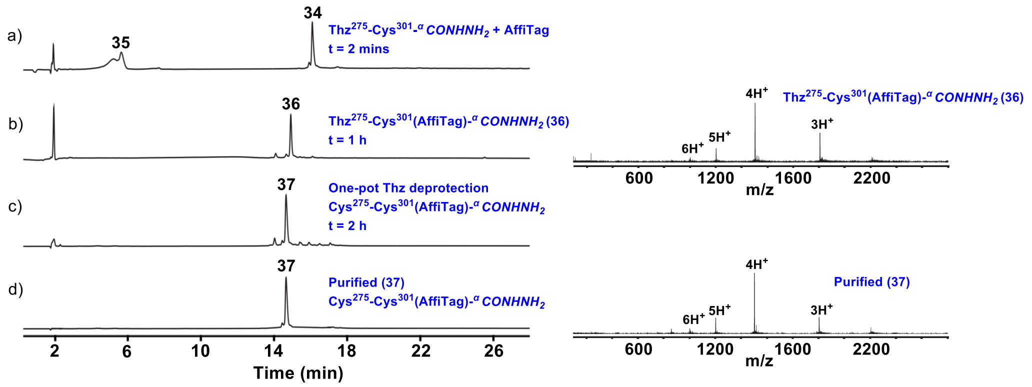

Figure S27. Analytical HPLC profile (λ= 214 nm) together with ESI-MS data (right). Chromatogram of (a) the reaction mixture at 2 minutes after the addition of peptide **34** and **35**. (b) the reaction at 1 h after the completion of conjugation of AffiTag (**35**) on **34** (c) one-pot Thz deprotection of peptide **36** after 1 h to yield peptide **37** (d) purified peptide **37**. Linear gradient 10%-64% of B over 27 min including 4 min equilibration using Agilent Zorbax SB-C3 5 µm 4.6 x 150 mm LC column with 0.9 mL/min flow rate was used for the chromatographic separation. Purification was performed using a linear gradient 10%-40% of buffer B in buffer A over 30 min with a flow rate of 5 mL/min at 40 °C (buffer A = 0.1% TFA in water; buffer B = 0.08% TFA in acetonitrile) using a C3, 9.4 x 250 mm column (Agilent ZORBAX-SB C3, 80 Å, 5 µm).

7.2. One-pot native chemical ligation of *Fmoc*-Cys^{217}-Lys^{246}-$^{\alpha}$*COSR* (18"), *Fmoc*-Cys^{247}-Phe^{274}- $^{\alpha}$*COSR* (19"), and Cys^{275}-Cys^{301}(AffiTag)-$^{\alpha}$*CONHNH$_2$* (37)

For the first ligation, the peptide segment Cys^{275}-Cys^{301}(AffiTag)-$^{\alpha}$*CONHNH$_2$* (**37**, 62.5 mg, 10.4 µmol) was dissolved in 10.0 mL degassed ligation buffer (200 mM phosphate buffer, 6 M Gu.HCl and 50 mM TCEP) containing 50 mM MPAA, and the buffer pH was adjusted to 6.8. Next, the peptide segment *Fmoc*-Cys^{247}-Phe^{274}- $^{\alpha}$*COSR* (**19"**, 41.98 mg, 12.2 µmol) was added to the reaction mixture as a solid powder. The pH of the reaction mixture was then readjusted to 6.82 and was allowed to react at room temperature to furnish the ligation product *Fmoc*-Cys^{247}-Cys^{301}(AffiTag)-$^{\alpha}$*CONHNH$_2$* (**38**) within 12 h (**Figure S28a-b**). To remove the Fmoc group of the ligated product **38** after the first ligation, ~800 µL concentrated HCl was added followed by ~2.3 mL piperidine to the reaction mixture to prevent a sudden abrupt change in pH and to fix the final concentration of piperidine to 20% (v/v). The final pH of the reaction mixture was immediately adjusted to 11.0 by using concentrated HCl (12 M) and aqueous NaOH (6 M). The reaction mixture was vortexed vigorously at room temperature. The complete Fmoc deprotection was observed within 7 min (including ~2 min time required for pH adjustment) to afford Cys^{247}-Cys^{301}(AffiTag)-$^{\alpha}$*CONHNH$_2$* (**39**) (**Figure S28c**). The pH of the reaction mixture was then rapidly brought down to ~9 by adding concentrated HCl (12 M) followed by the addition of TCEP as a solid powder (final concentration 50 mM). The pH of the reaction mixture was then adjusted to 6.88. For the second ligation, peptide segment *Fmoc*-Cys^{217}-Lys^{246}-$^{\alpha}$*COSR* (**18"**, 47.05 mg, 12.2 µmol) was then added to the reaction mixture and the pH was adjusted back to 6.89. Within 32 h, the peptide **39** was completely converted to the desired ligation product *Fmoc*-Cys^{217}- Cys^{301}(AffiTag)-$^{\alpha}$*CONHNH$_2$* (**40**) (**Figure S28c-d**). To deprotect the Fmoc group from peptide **40**, additional piperidine (to adjust the total concentration to 20% (v/v)) and 6 M NaOH (to increase the pH to 11.0) were added to the reaction mixture.

The reaction mixture was then vortexed for a total of 7 min (including ~2 min time required for adjusting pH) to afford the final polypeptide **41** (**Figure S28e**). As before, the pH of the reaction buffer was rapidly reduced to ~9.0 by adding concentrated HCl followed by the addition of TCEP (final concentration 50 mM) as solid powder, and the pH was readjusted to 7.0 to achieve complete disulfide reduction. After the two ligations, and two Fmoc deprotection steps, HPLC purification was performed to obtain the pure full-length polypeptide Cys^{217}-Cys^{301}(AffiTag)-$^{\alpha}$*CONHNH$_2$* (**41**, 10.9 mg, 1.08 µmol) with an overall 32% yield. The purity and identity of the *Pf*AMA1 polypeptide **41** were confirmed by LC-MS (**Figure S28f**). Observed mass (**ESI-MS**): 10759.46 ± 0.12 Da (average of the five most abundant charge states); calculated mass: 12758.97 Da (average isotope composition).

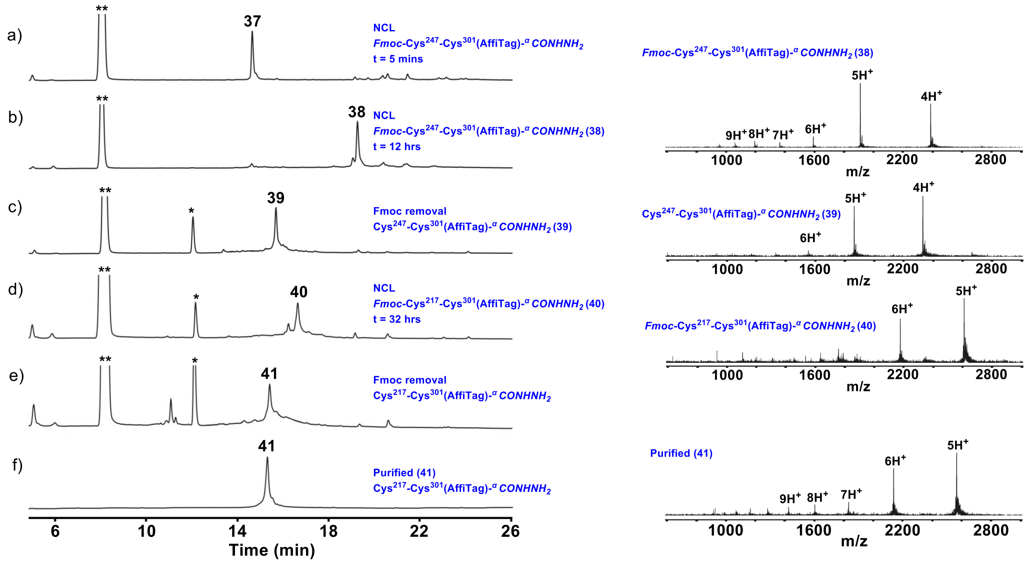

Figure S28. Analytical HPLC monitoring of the one-pot ligation reaction: a) 5 min after the addition of the peptide Cys^{275}-Cys^{301}(AffiTag)-$^{\alpha}$*CONHNH$_2$* (**37**) and *Fmoc*-Cys^{247}-Phe^{274}-$^{\alpha}$*COSR* (**19''**) in the standard ligation buffer (200 mM PB, 6 M Gu.HCl, 50 mm TCEP) containing 50 mM MPAA. **19''** was not visible in the chromatogram (multiple thiolactones were identified); b) The first ligation, within 12 h, resulted in *Fmoc*-Cys^{247}-Cys^{301}(AffiTag)-$^{\alpha}$*CONHNH$_2$* (**38**) as the ligated product; c) Fmoc removal from the ligated peptide **38** to give peptide Cys^{247}-Cys^{301}(AffiTag)-$^{\alpha}$*CONHNH$_2$* (**39**); d) The second ligation was essentially complete within 32 h after the addition of peptide Fmoc-Cys^{217}–Lys^{246}-$^{\alpha}$COSR (**18''**) in the reaction mixture and furnished polypeptide *Fmoc*-Cys^{217}- Cys^{301}(AffiTag)-$^{\alpha}$*CONHNH$_2$* (**40**) e) Fmoc removal from the ligated product **40** to give target peptide Cys^{217}- Cys^{301}(AffiTag)-$^{\alpha}$*CONHNH$_2$* (**41**) (f) purified peptide **41**. * and ** indicate dibenzofulvene-TECP adduct and MPAA, respectively. Linear gradient 10%-64% of B over 27 min including 4 min equilibration using Agilent Zorbax SB-C3 5 µm 4.6 x 150 mm LC column with 0.9 mL/min flow rate was used for the chromatographic separation. Purification was performed using a linear gradient 15%-45% of buffer B in buffer A over 90 min with a flow rate of 5 mL/min at 40 °C (buffer A = 0.1% TFA in water; buffer B = 0.08% TFA in acetonitrile) using a C3, 9.4 x 250 mm column (Agilent ZORBAX-SB C3, 80 Å, 5 µm).

7.3. Native chemical ligation of *Fmoc*-Cys-Gly-Ser-Gly-His^{123}-Leu^{216}-Dbz-Arg$_4$-$^{\alpha}$*CONH$_2$* (25) and Cys^{217}-Cys^{301}(AffiTag)-$^{\alpha}$*CONHNH$_2$* (41)

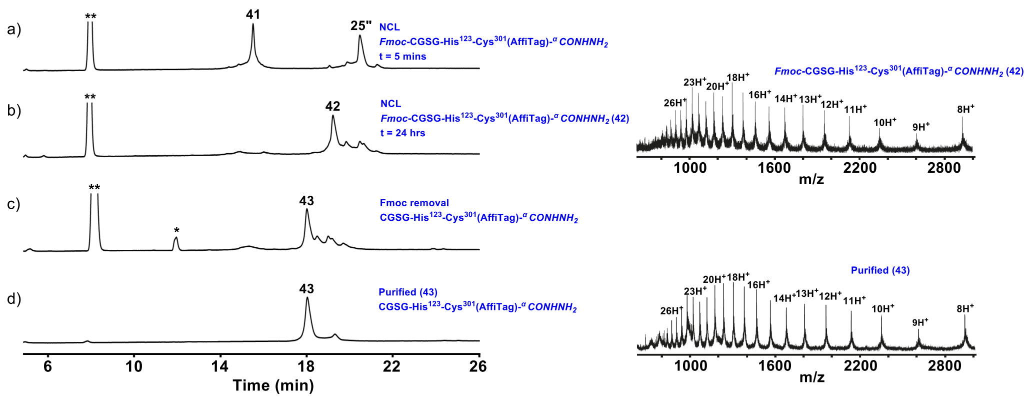

Figure S29. Analytical RP-HPLC profile (λ= 214 nm) together with ESI-MS data (right) of the ligation reaction. Chromatogram of (a) the reaction at 5 min after the addition of the peptide Cys^{217}-Ala^{301}-$^{\alpha}$*CONHNH$_2$* (**41**) to *Fmoc*-Cys-Gly-Ser-Gly-His^{123}-Leu^{216}-Dbz-Arg$_4$ - $^{\alpha}$*MPAA* (**25''**) peptide (c) the ligation, within 24 h, resulted in *Fmoc*- Cys-Gly-Ser-Gly-His^{123}-Cys^{301}(AffiTag)-$^{\alpha}$*CONHNH$_2$* (**42**) as the ligated product (c) Fmoc removal from the ligated

peptide **42** to give peptide Cys-Gly-Ser-Gly-His^{123}-Cys^{301}(AffiTag)-$^{\alpha}$*CONHNH$_2$* (**43**) (d) purified peptide **43.** Linear gradient 10%-64% of B over 27 min including 4 min equilibration time using an Agilent Zorbax SB-C3, 5 µm, 4.6 x 150 mm LC column with 0.9 mL/min flow rate was used for the chromatographic separation. Purification was carried out using Agilent zorbax SB-C3 5 µm, 9.4 x 250 mm, LC column using a linear gradient of 15%-50% of buffer B over 70 min with a flow rate of 5 mL/min at 40 °C. The '**' indicates MPAA.

Peptide *Fmoc*-Cys-Gly-Ser-Gly-His^{123}-Leu^{216}-Dbz-Arg$_4$-$^{\alpha}$*CONH$_2$* (**25,** 20 mg, 1.71 µmol) was dissolved in 4 mL of aqueous phosphate buffer (0.2 M) containing 6 M Gu.HCl at pH 3.0 and kept at -16.5 °C. After 15 min, 0.4 mL aqueous $NaNO_2$ (0.5 M) was added to the solution of peptide **25** and gently agitated at -16.5 °C for 15 min. Then, 4 mL of 0.2 M MPAA in a pH 6.5 buffer (200 mM phosphate buffer, 6 M Gu.HCl) was added to the oxidized solution of peptide **25** and the temperature was raised to room temperature. Afterward, 21.8 mg (1.71 µmol) of the second peptide segment Cys^{217}-Cys^{301}(AffiTag)-$^{\alpha}$*CONHNH$_2$* **(41)** was added to the reaction mixture as solid powder and the pH was adjusted to 6.85. The ligation was monitored by analytical HPLC and ESI-MS as shown in **Figure S29a-b**. To deprotect the Fmoc group from peptide **42**, piperidine (to adjust the total concentration to 20% (v/v)) and 6 M NaOH (to increase the pH to 11.0) were added to the reaction mixture. The reaction mixture was then vortexed for a total of 7 min (including ~2 min time required for adjusting pH) to afford the final polypeptide **43**. The pH of the reaction buffer was rapidly reduced to ~9.0 by adding concentrated HCl followed by the addition of TCEP (final concentration 50 mM) as solid powder, and the pH was readjusted to 7.0 to achieve complete disulfide reduction. Purification of the reaction mixture gave 18.7 mg (0.80 µmol, 47% yield) of the pure ligated polypeptide, Cys-Gly-Ser-Gly-His^{123}-Cys^{301}(AffiTag)-$^{\alpha}$*CONHNH$_2$* (**43**). Observed mass **(ESI-MS)**: 23412.47 ± 0.29 Da (average of the eight most abundant charge states), calculated mass: 23412.22 Da (average isotope composition) (**Figure S29d**).

7.4. Cyclization and folding of Seg-1+2+3+4+5+6(AffiTag)-$^{\alpha}$*CONHNH$_2$* (43)

Peptide Cys-Gly-Ser-Gly-His^{123}-Cys^{301}(AffiTag)-$^{\alpha}$*CONHNH$_2$* (**43,** 15.0 mg, 0.64 µmol) was dissolved in 15 mL of aqueous phosphate buffer (0.2 M) containing 6 M Gu.HCl at pH 3.0 and kept at -16.5 °C. After 15 min, 1.5 mL aqueous $NaNO_2$ (0.050 M) was added to the solution of peptide **43** and gently agitated at -16.5 °C for 15 min. Then, 7.5 mL of 0.15 M MPAA in a pH 6.4 buffer (200 mM phosphate buffer, 6 M Gu.HCl) was added to the oxidized solution of peptide **43** and the temperature was raised to room temperature. The pH of the reaction buffer was adjusted to 6.5 and kept at RT until the completion of the cyclization. The cyclization was monitored by analytical HPLC and ESI-MS as shown in **Figure S30a-b**. Purification of the reaction mixture gave 7.25 mg (0.31 µmol, 49.5 % yield) of the pure polypeptide, cyclic(Cys-Gly-Ser-Gly-His^{123}-Cys^{301}(AffiTag)) (**44**). Observed mass **(ESI-MS)**: 23380.33 ± 0.25 Da (average of the eight most abundant charge states), calculated mass: 23380.18 Da (average isotope composition) (**Figure S30c**).

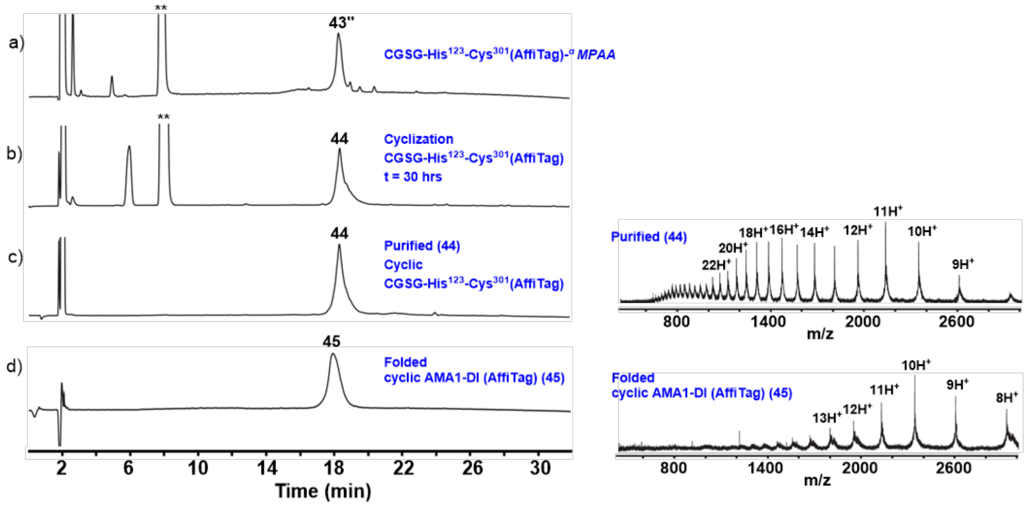

Figure S30. RP-HPLC profile (λ= 214 nm) together with ESI-MS data (right) of the cyclization of peptide **31**. Chromatogram of (a) peptide Cys-Gly-Ser-Gly-His^{123}-Cys^{301}(AffiTag)-$^{\alpha}$*MPAA* (**43"**) after $NaNO_2$ mediated oxidation and MPAA exchange. (b) the reaction after 30 h resulting in the formation of cyclized peptide **44.** (c) purified cyclized peptide **44** (d) folded cyclic *Pf*AMA1-DI (**45**). Linear gradient 10%-64% of B over 27 min including 4 min equilibration time using an Agilent Zorbax SB-C3, 5 µm, 4.6 x 150 mm LC column with 0.9 mL/min flow rate was used for the chromatographic separation.

Purification of peptide **44** was carried out using Agilent zorbax SB-C3 5 µm, 9.4 x 250 mm, LC column using a linear gradient of 15%-50% of buffer B over 70 min with a flow rate of 5 mL/min at 40 °C. The '**' indicates MPAA.

Folding: Purified peptide cyclic(Cys-Gly-Ser-Gly-His^{123}-Cys^{301}(AffiTag)) (**44,** 5 mg, 0.213 µmol**)** was dissolved in 0.83 mL buffer (6 M Gu.HCl, 20 mM phosphate, 1 mM GSH, 0.1 mM GSSG, 100 mM NaCl, pH 8.2) and kept at 4°C for 30 minutes. To this solution, 2.29 mL buffer (20 mM phosphate, 1 mM GSH, 0.1 mM GSSG, 100 mM NaCl, pH 8.2) was added four times in a 2-hour time interval to reduce the concentration of Gu.HCl from 6 M to 0.5 M and the final volume of the mixture was 8 mL. (*Note*: Buffers were purged with N_2 gas via bubbling prior to the start of the folding procedure). The reaction was monitored for 48 hours, and completion of the folding was confirmed by analytical HPLC and ESI-MS as shown in **Figure S30d** which shows a -6 Da mass difference from the reduced polypeptide **44**. Observed mass **(ESI-MS)**: 23374.31 ± 0.19 Da (average of the five most abundant charge states), calculated mass: 23374.14 Da (average isotope composition) (**Figure S30d**). Precipitates were removed from the reaction mixture by centrifugation (6000 rpm, 5 minutes, 4°C x 3). Remaining 0.5 M Gu.HCl and the redox reagents were removed from the reaction via dialysis (10 KDa membrane) (dialysis buffer: 10 mM phosphate, 100 mM NaCl, pH 7.8, 4°C x 2). The concentration of the folded cyclic(Cys-Gly-Ser-Gly-His^{123}- Cys^{301}(AffiTag)) (**45**) was determined by IMPLEN Spectrophotometer (NanoPhotometer® NP80) at 280 nm using the extinction coefficients derived from the protein sequence. The overall folding of the cyclic(Cys-Gly-Ser-Gly-His^{123}- Cys^{301}(AffiTag)) peptide gave 1.0 mg (0.043 µmol, 20.2% yield) folded cyclic *Pf*AMA1-DI (AffiTag) protein (**45, Figure S30d**).

8. Methionine oxide reduction to methionine (*Pf*AMA1 Seg-5 *Fmoc*-Cys^{247}–Phe^{274}-$^{\alpha}$*CONHNH$_2$* (19))

Methionine oxide was reduced to methionine using the reported[3] protocol with some modification. The crude peptide *Fmoc*-Cys-His-Ile-Leu-Tyr-Ile-Ala-Ala-Gln-Glu-Asn-Asn-Gly-Pro-Arg-Tyr-Cys-Asn-Lys-Asp-Glu-Ser-Lys-Arg-Asn-Ser-Met-Phe-$^{\alpha}$*CONHNH$_2$* (**19**) (106.5 mg, 30.1 µmol) containing oxidized-Met peptide by-product (**21**) was dissolved in TFA (5 mL) and cooled to 0 °C. Afterwards, 2,2'-(Ethylenedioxy)diethanethiol (DODT) (195 µL, 1.2 mmol, 40.0 eq.) and trimethylsilyl bromide (TMBS) (79.5 µL, 0.602 mmol, 20.0 eq.) were added. The solution was equilibrated under gentle movement at 0 °C for 15 min. The reaction was monitored by analytical HPLC and ESI-MS as shown in **Figure S31a-b**. After the complete reduction of methionine oxide, TFA was removed via N_2 bubbling and cold ether was added to precipitate the desired peptide *Fmoc*-Cys^{247}–Phe^{274}-$^{\alpha}$*CONHNH$_2$* (**19**). Precipitates were washed with cold ether (x3) and lyophilized.

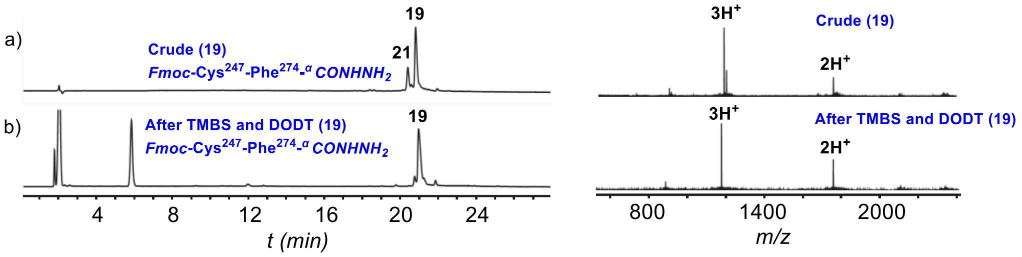

Figure S31. RP-HPLC profile (λ= 214 nm) together with ESI-MS data (right) for the reversal of peptide **21** to peptide **19**. Chromatogram of (a) crude polypeptide *Fmoc*-Cys^{247}–Phe^{274}-$^{\alpha}$*CONHNH$_2$* (**19**) with methionine-oxide by-product **21** (b) the reaction at 10 min after the addition of TMBS and DODT in TFA at 0°C containing crude peptide mixture of **19** and **21** resulting in only peptide **19** as product. Linear gradient 10%-54% of B over 22 min including 4 min equilibration time using an Agilent Zorbax SB-C3, 5 µm, 4.6 x 150 mm LC column with 0.9 mL/min flow rate was used for the chromatographic separation.

9. Evaluation of binding activity by surface plasmon resonance (SPR)

9.1. Solid phase synthesis of *Pf*RON2$_{2021-2059}$ peptide

The folded *Pf*RON2$_{2021-2059}$ was synthesized using the protocol previously reported by our group.[9a] In brief, the peptide sequence Asp-Ile-Thr-Gln-Gln-Ala-Lys-Asp-Ile-Gly-Ala-Gly-Pro-Val-Ala-Ser-Cys-Phe-Thr-Thr-Arg-Met-Ser-Pro-Pro-Gln-Gln-Ile-Cys-Leu-Asn-Ser-Val-Val-Asn-Thr-Ala-Leu-Ser-$^{\alpha}$*CONH$_2$* was synthesized on Rink-Amide aminomethyl-resin (substitution= 0.30 mmol/g) by stepwise Fmoc chemistry SPPS in 0.05 mmol scale using an automated peptide synthesizer (*see* **Section 1.3** for the peptide synthesis protocol). After global

deprotection using a TFA cocktail, the crude peptide *Pf*RON2$_{2021\text{-}2059}$ was precipitated using diethyl ether and lyophilized. The observed mass (ESI-MS) was 4063.02 Da (deconvoluted most abundant isotopologue); calculated mass: 4063.01 Da (most abundant isotopologue). Crude peptide (50 mg, 12.3 µmol) was dissolved in 50 mL of 6 M Gu.HCl, 50 mM Tris buffer at pH 8.0 and left under air oxidation for disulfide formation. The reaction was monitored by analytical HPLC and ESI-MS as published elsewhere[5] and after complete folding, was purified to afford 32.9 mg (8.1 µmol, 65.8%) of the folded *Pf*RON2$_{2021\text{-}2059}$. The observed mass (ESI-MS) was 4061.02 Da (deconvoluted most abundant isotopologue); calculated mass: 4060.99 Da (most abundant isotopologue).

9.2. SPR binding of AffiTagged cyclic *Pf*AMA1-DI and *Pf*RON2$_{2021\text{-}2059}$

SPR measurements were conducted using a BI-4500AP SPR Instrument. In the case of cyclic *Pf*AMA1-DI (**33**), dextran-sensor chip was used for binding studies. For surface immobilization on the dextran-sensor chip, EDC and NHS were used to activate the carboxyl group of the dextran and cyclic *Pf*AMA1-DI was used as a ligand. 0.4 M of EDC in milliQ and 0.1 M of NHS in milliQ were prepared separately and were mixed in 1:1 ratio. To activate the surface of the chip, this solution was run for 7 min at 11 µL/min flow rate followed by the injection of 4 µM chemically synthesized cyclic *Pf*AMA1-DI ligand (10 mM phosphate, 100 mM NaCl) at the same flow rate for 4 min. This was followed by the addition of 1 M ethanolamine to block the remaining activated sites (if any) on the surface of the chip. For the binding study, different concentrations of analyte *Pf*RON2$_{2021\text{-}2059}$ (200 nM, 400 nM, 800 nM, 1.6 µM, 3.2 µM) were used (10 mM phosphate buffer, 100 mM NaCl, pH 7.8) at 30 µL/min flow rate. Our attempt to determine proper binding data was unsuccessful (data not shown), likely due to disruptions in the protein functional fold caused by the surface immobilization. To avoid this, pre-functionalized streptavidin sensor chips were taken allowing us to utilize C-terminal Biotin tagged *Pf*RON2$_{2021\text{-}2059}$ (10 µM; its chemical synthesis was reported[16d] elsewhere) as a ligand to be immobilized on the surface of the sensor chip. In this case, chemically synthesized cyclic *Pf*AMA1-DI (1.0 µM) was used as an analyte which resulted in large non-specific interaction (data not shown) of cyclic *Pf*AMA1-DI with either pre-functionalized streptavidin or with the surface of the chip resulting in unsuccessful determination of the binding affinity.

For gentler immobilization, the chemically synthesized AffiTagged cyclic *Pf*AMA1-DI protein (**45,** 5 µM, pH 7.80) containing His$_6$ was immobilized on a Nickel-NTA chip, with one flow cell serving as the reference channel. Binding assays between cyclic *Pf*AMA1-DI(AffiTag) (**45**) and *Pf*RON2$_{2021\text{-}2059}$ protein were performed at a temperature of 25 °C using buffer (10 mM Phosphate buffer saline, 100 mM NaCl, pH 7.8) supplemented with 0.005% Tween-20 and 40 µM EDTA as the running buffer. Various concentrations of the *Pf*RON2$_{2021\text{-}2059}$ protein (in 10 mM phosphate buffer saline, 100 mM NaCl, pH 7.4) were injected at a constant flow rate of 30 µL/min to generate the sensorgrams. Peptide dissociation was initiated by halting sample injections and flowing the running buffer at the same rate. The final sensorgrams were obtained by subtracting the sensorgram of the control flow cell from the ligand flow cell sensorgrams to eliminate non-specific binding signals. The interactions between cyclic *Pf*AMA1-DI and *Pf*RON2$_{2021\text{-}2059}$ were analyzed using the BI-data analysis software.

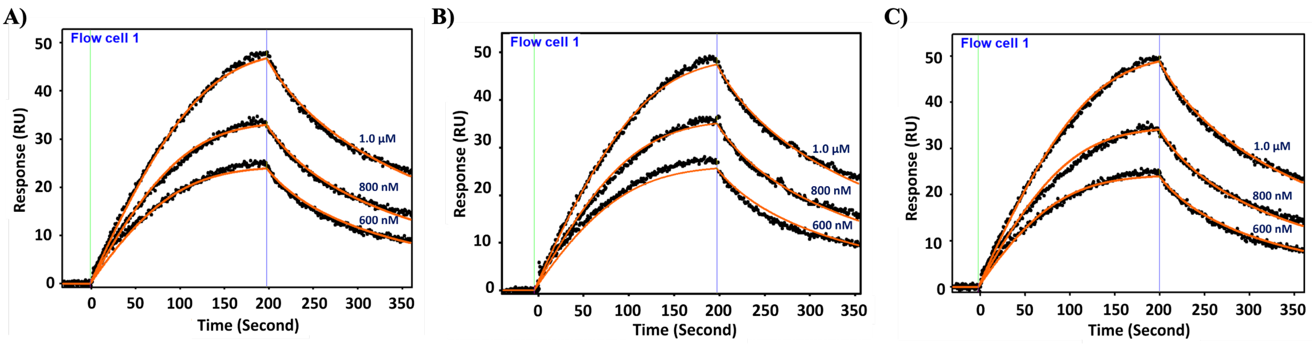

Sr. No.	K_a ($M^{-1}s^{-1}$)	K_d (s^{-1})	K_D (nM)	Average K_D (nM)
A)	1.43 x 10^5	6.95 x 10^{-2}	486.10	500.24 ± 14
B)	1.26 x 10^5	6.34 x 10^{-2}	501.16	
C)	1.55 x 10^5	7.95 x 10^{-2}	513.48	

Figure S32. SPR sensorgrams illustrate the binding of the analyte, chemically synthesized *Pf*RON2$_{2021-2059}$, with the ligand chemically synthesized cyclic *Pf*AMA1-DI(AffiTag) (**45**). One of the three repeats of the binding events are shown in the figure (A), (B) and (C). The kinetic fitting analyses (orange curve) were performed using non-linear regression fits to a 1:1 Langmuir adsorption isotherm applied to the experimental data points. The average K_D value obtained from the three repeats was 500.24 ± 14 nM (Ka = (1.42 ± 0.14) × 10^5 $M^{-1}s^{-1}$; Kd = (7.08 ± 0.81) × 10^{-2} s^{-1}).

The binding data were analyzed using a 1:1 Langmuir adsorption binding isotherm to evaluate the kinetic parameters. Triplicate experiments were conducted (**Figure S32**), and the mean values and standard deviation of the association rate constant (Ka), dissociation rate constant (Kd), and overall dissociation constant (K_D) were determined. The analysis yielded a Ka of (1.42 ± 0.14) × 10^5 $M^{-1}s^{-1}$, a Kd of (7.08 ± 0.81) × 10^{-2} s^{-1}, and a K_D of 500.24 ± 14 nM, indicating a binding affinity consistent with expectations for the interaction between chemically synthesized cyclic*Pf*AMA1-DI and *Pf*RON2$_{2021-2059}$.

10. Web-based prediction of cyclic*Pf*AMA1-DI/ *Pf*RON2$_{2021-2059}$ complex

To elucidate the structural conformation of cyclic*Pf*AMA1-D1 and its interaction with *Pf*RON2$_{2021-2059}$, we employed ColabFold-AlphaFold2, a deep learning-based modeling tool implemented within the Google Colaboratory platform. Utilizing a cyclic peptide complex offset protocol described by Kosugi et al.,[26] this method applies cyclic positional encoding to the cyclic peptide while preserving default encoding for the protein region. The approach has demonstrated exceptional accuracy for cyclic peptide structures, achieving high confidence scores (pLDDT > 85%) and root mean squared deviation (RMSD) values below 1.5 Å, surpassing established docking tools such as AutoDock CrankPep (ADCP).[27] In this study, we adapted this protocol to predict cyclic protein structures. The parameter we used for ColabFold-cycpep-dock based on ColabFold v1.5.2-patch with AlphaFold2 using MMseqs2 with alphafold2_multimer_v3 with no template information is used for prediction.

For our analysis, the cyclic offset was exclusively applied to the cyclic *Pf*AMA1-D1 region, while default encoding was retained for *Pf*RON2$_{2021-2059}$. 36 de novo cyclic protein-peptide complex models generated with varying circularly permuted sites (**Figure S33**) to achieve high confidence models. The parameters and configurations for all the protein-cyclic peptide complex prediction were kept same while running the notebook. The input sequences of the protein-cyclic peptide complex as well as the obtained predicted local distance difference test (pLDDT), predicted template modeling score (pTM) and interface pTM (ipTM) values for best predicted model i.e. rank 1 model are listed **SI Table 1** below. In the case of complex model, ColabFold ranks the predicted protein model by taking the output values from pLDDT, pTM and ipTM by using the formula: (0.2xpTM + 0.8xiPTM). Among 36 de novo structural predictions, five models achieved exceptionally high confidence, with pLDDT scores exceeding 90%, the highest being 91.8%. Representative structures of the top five predicted cyclic*Pf*AMA1-D1/*Pf*RON2$_{2021-2059}$ complexes are depicted in **Figure S34**.

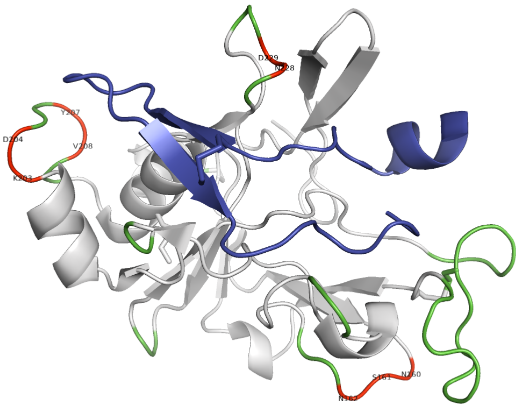

Figure S33. Crystal structure of *Pf*AMA1-DI (grey) in complex with *Pf*RON2$_{2021-2059}$ (blue), derived from PDB: 3ZWZ[7]. Residues highlighted in green represent solvent-exposed, unstructured regions of *Pf*AMA1-DI, selected as the cut sites for structural predictions using ColabFold-AlphaFold2. Red-highlighted residues, annotated with corresponding labels, indicate cut sites that resulted in predicted structures with pLDDT scores exceeding 90.

The highest-ranked model revealed that *Pf*RON2$_{2021-2059}$ occupies the hydrophobic groove of cyclic*Pf*AMA1-D1 in a configuration analogous to its binding mode with full-length *Pf*AMA1 (DI+DII). However, the N-terminal α-helix of *Pf*RON2$_{2021-2059}$ exhibited a slight

outward tilt from the DII-loop region, presumably due to the absence of domain II in the cyclic *Pf*AMA1-D1 construct. Previous molecular dynamics[28] simulations have suggested that the DII loop undergoes a dynamic "breathing motion," transiently interacting with *Pf*RON2ed residues (Ile2022, Gln2024, Gln2025, Lys2027, Asp2028), contacts typically undetectable in static structures such as X-ray crystallography or AlphaFold2. Binding affinity measurements using SPR for a truncated *Pf*RON2$_{2027-2055}$ complex with *Pf*AMA1 (DI+DII) show an affinity of ~520 nM[6a], closely matching the affinity of 500 nM observed in our SPR analysis with the cyclic *Pf*AMA1-D1/*Pf*RON2$_{2021-2059}$ complex.

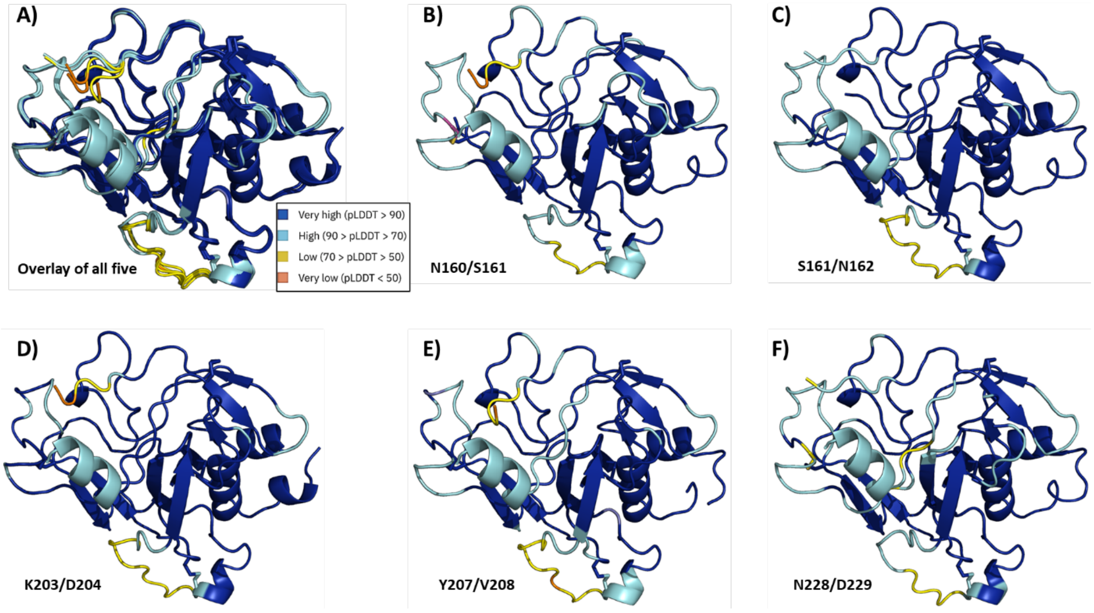

Figure S34. Predicted cyclic structures of *Pf*AMA1-DI generated using ColabFold-AlphaFold2, with all models exhibiting pLDDT scores exceeding 90, indicating high confidence. Structures are colored according to their pLDDT scores, as shown in the inset color scale. (A) Overlay of the five predicted cyclic structures. (B) Predicted structure with a cut site between Asn160 and Ser161 (pLDDT = 90.6, ipTM = 0.868). (C) Predicted structure with a cut site between Ser161 and Asn162 (pLDDT = 91.8, ipTM = 0.892). (D) Predicted structure with a cut site between Lys203 and Lys204 (pLDDT = 91.8, ipTM = 0.881). (E) Predicted structure with a cut site between Tyr207 and Val208 (pLDDT = 90.1, ipTM = 0.856). (F) Predicted structure with a cut site between Asn228 and Asp229 (pLDDT = 90.6, ipTM = 0.873).

Table S1. Predicted structural models of cyclic *Pf*AMA1-D1/*Pf*RON2ed complexes generated using ColabFold-cycpep-dock-AlphaFold2-MMseqs2 with varying circular permutation sites. The table provides details on the circularly permuted residue sites, input sequences, and model accuracy metrics, including predicted local distance difference test (pLDDT) scores, predicted template modeling (pTM) scores, and interface pTM (ipTM) scores.

**Sr. No**	**Cut site**	**Input Sequences for Structure Prediction Using ColabFold-cycpep-dock (AlphaFold2-MMseqs2)**	**Model confidence**		
			**pLDDT**	**pTM**	**ipTM**
1*	N.A./Linear *Pf*AMA1-DI	HGSGIRVDLGEDAEVAGTQYRLPSGKCPVFGKGIIIENSNTTFLTPVATGNQYLKDGGFAFPPT<br>EPLMSPMTLDEMRHFYKDNKYVKNLDELTLCSRHAGNMIPDNDKNSNYKYPAVYDDKDKKC<br>HILYIAAQENNGPRYCNKDESKRNSMFCFRPAKDISFQNYTYLSKNVVDNWEKVC	92.4	0.884	
2*	N.A./Linear *Pf*AMA1-DI/*Pf*RON2	HGSGIRVDLGEDAEVAGTQYRLPSGKCPVFGKGIIIENSNTTFLTPVATGNQYLKDGGFAFPPT<br>EPLMSPMTLDEMRHFYKDNKYVKNLDELTLCSRHAGNMIPDNDKNSNYKYPAVYDDKDKKC<br>HILYIAAQENNGPRYCNKDESKRNSMFCFRPAKDISFQNYTYLSKNVVDNWEKVC:DITQQAK<br>DIGAGPVASCFTTRMSPPQQICLNSVVNTALS	89.1	0.891	0.81
3*	*N.A./Pf*AMA1 (DI+DII)-*Pf*RON2	GSAMGNYMGNPWTEYMAKYDIEEVHGSGIRVDLGEDAEVAGTQYRLPSGKCPVFGKGIIIEN<br>SNTTFLTPVATGNQYLKDGGFAFPPTEPLMSPMTLDEMRHFYKDNKYVKNLDELTLCSRHAG<br>NMIPDNDKNSNYKYPAVYDDKDKKCHILYIAAQENNGPRYCNKDESKRNSMFCFRPAKDISF<br>QNYTYLSKNVVDNWEKVCPRKNLQNAKFGLWVDGNCEDIPHVNEFPAIDLFECNKLVFELSA<br>SDQPKQYEQHLTDYEKIKEGFKNKNASMIKSAFLPTGAFKADRYKSHGKGYNWGNYNTETQK<br>CEIFNVKPTCLINNSSYIATTALSHPIEVEAAALVPR:DITQQAKDIGAGPVASCFTTRMSPPQQI<br>CLNSVVNTALS	87.4	0.867	0.914
4	*Pf*AMA1-DI-Cyclic-C302/A301	DITQQAKDIGAGPVASCFTTRMSPPQQICLNSVVNTALS:CGSGHGSGIRVDLGEDAEVAGTQ<br>YRLPSGKCPVFGKGIIIENSNTTFLTPVATGNQYLKDGGFAFPPTEPLMSPMTLDEMRHFYKDN<br>KYVKNLDELTLCSRHAGNMIPDNDKNSNYKYPAVYDDKDKKCHILYIAAQENNGPRYCNKDE<br>SKRNSMFCFRPAKDISFQNYTYLSKNVVDNWEKA	87.2	0.88	0.823
5	*Pf*AMA1-DI-Cyclic-G119/C302	DITQQAKDIGAGPVASCFTTRMSPPQQICLNSVVNTALS:GSGHGSGIRVDLGEDAEVAGTQY<br>RLPSGKCPVFGKGIIIENSNTTFLTPVATGNQYLKDGGFAFPPTEPLMSPMTLDEMRHFYKDNK	87.3	0.882	0.833

		YVKNLDELTLCSRHAGNMIPDNDKNSNYKYPAVYDDKDKKCHILYIAAQENNGPRYCNKDES<br>KRNSMFCFRPAKDISFQNYTYLSKNVVDNWEKAC			
6	*Pf*AMA1-DI-<br>Cyclic-<br>H123/G122	DITQQAKDIGAGPVASCFTTRMSPPQQICLNSVVNTALS:HGSGIRVDLGEDAEVAGTQYRLPS<br>GKCPVFGKGIIIENSNTTFLTPVATGNQYLKDGGFAFPPTEPLMSPMTLDEMRHFYKDNKYVK<br>NLDELTLCSRHAGNMIPDNDKNSNYKYPAVYDDKDKKCHILYIAAQENNGPRYCNKDESKRN<br>SMFCFRPAKDISFQNYTYLSKNVVDNWEKACGSG	87.7	0.884	0.833
7	*Pf*AMA1-DI-<br>Cyclic-<br>N160/S161	DITQQAKDIGAGPVASCFTTRMSPPQQICLNSVVNTALS:SNTTFLTPVATGNQYLKDGGFAFP<br>PTEPLMSPMTLDEMRHFYKDNKYVKNLDELTLCSRHAGNMIPDNDKNSNYKYPAVYDDKDK<br>KCHILYIAAQENNGPRYCNKDESKRNSMFCFRPAKDISFQNYTYLSKNVVDNWEKACGSGH<br>GSGIRVDLGEDAEVAGTQYRLPSGKCPVFGKGIIIEN	90.6	0.904	0.868
8	*Pf*AMA1-DI-<br>Cyclic-<br>S161/N162	DITQQAKDIGAGPVASCFTTRMSPPQQICLNSVVNTALS:NTTFLTPVATGNQYLKDGGFAFPP<br>TEPLMSPMTLDEMRHFYKDNKYVKNLDELTLCSRHAGNMIPDNDKNSNYKYPAVYDDKDKK<br>CHILYIAAQENNGPRYCNKDESKRNSMFCFRPAKDISFQNYTYLSKNVVDNWEKACGSGHG<br>SGIRVDLGEDAEVAGTQYRLPSGKCPVFGKGIIIENS	91.8	0.91	0.892
9	*Pf*AMA1-DI-<br>Cyclic-<br>N162/T163	DITQQAKDIGAGPVASCFTTRMSPPQQICLNSVVNTALS:TTFLTPVATGNQYLKDGGFAFPPT<br>EPLMSPMTLDEMRHFYKDNKYVKNLDELTLCSRHAGNMIPDNDKNSNYKYPAVYDDKDKKC<br>HILYIAAQENNGPRYCNKDESKRNSMFCFRPAKDISFQNYTYLSKNVVDNWEKACGSGHGS<br>GIRVDLGEDAEVAGTQYRLPSGKCPVFGKGIIIENSN	87.5	0.884	0.841
10	*Pf*AMA1-DI-<br>Cyclic-<br>T163/T164	DITQQAKDIGAGPVASCFTTRMSPPQQICLNSVVNTALS:TFLTPVATGNQYLKDGGFAFPPTE<br>PLMSPMTLDEMRHFYKDNKYVKNLDELTLCSRHAGNMIPDNDKNSNYKYPAVYDDKDKKCH<br>ILYIAAQENNGPRYCNKDESKRNSMFCFRPAKDISFQNYTYLSKNVVDNWEKACGSGHGSGI<br>RVDLGEDAEVAGTQYRLPSGKCPVFGKGIIIENSNT	88.7	0.891	0.852
11	*Pf*AMA1-DI-<br>Cyclic-<br>T171/G172	DITQQAKDIGAGPVASCFTTRMSPPQQICLNSVVNTALS:GNQYLKDGGFAFPPTEPLMSPMT<br>LDEMRHFYKDNKYVKNLDELTLCSRHAGNMIPDNDKNSNYKYPAVYDDKDKKCHILYIAAQE<br>NNGPRYCNKDESKRNSMFCFRPAKDISFQNYTYLSKNVVDNWEKACGSGHGSGIRVDLGED<br>AEVAGTQYRLPSGKCPVFGKGIIIENSNTTFLTPVAT	89.8	0.902	0.871
12	*Pf*AMA1-DI-<br>Cyclic-<br>G172/N173	DITQQAKDIGAGPVASCFTTRMSPPQQICLNSVVNTALS:NQYLKDGGFAFPPTEPLMSPMTL<br>DEMRHFYKDNKYVKNLDELTLCSRHAGNMIPDNDKNSNYKYPAVYDDKDKKCHILYIAAQEN<br>NGPRYCNKDESKRNSMFCFRPAKDISFQNYTYLSKNVVDNWEKACGSGHGSGIRVDLGEDA<br>EVAGTQYRLPSGKCPVFGKGIIIENSNTTFLTPVATG	88.1	0.892	0.854
13	*Pf*AMA1-DI-<br>Cyclic-<br>P188/L189	DITQQAKDIGAGPVASCFTTRMSPPQQICLNSVVNTALS:LMSPMTLDEMRHFYKDNKYVKNL<br>DELTLCSRHAGNMIPDNDKNSNYKYPAVYDDKDKKCHILYIAAQENNGPRYCNKDESKRNS<br>MFCFRPAKDISFQNYTYLSKNVVDNWEKACGSGHGSGIRVDLGEDAEVAGTQYRLPSGKCP<br>VFGKGIIIENSNTTFLTPVATGNQYLKDGGFAFPPTEP	85.6	0.877	0.825
14	*Pf*AMA1-DI-<br>Cyclic-<br>K203/D204	DITQQAKDIGAGPVASCFTTRMSPPQQICLNSVVNTALS:DNKYVKNLDELTLCSRHAGNMIP<br>DNDKNSNYKYPAVYDDKDKKCHILYIAAQENNGPRYCNKDESKRNSMFCFRPAKDISFQNYT<br>YLSKNVVDNWEKACGSGHGSGIRVDLGEDAEVAGTQYRLPSGKCPVFGKGIIIENSNTTFLTP<br>VATGNQYLKDGGFAFPPTEPLMSPMTLDEMRHFYK	91.8	0.912	0.881
15	*Pf*AMA1-DI-<br>Cyclic-<br>D204/N205	DITQQAKDIGAGPVASCFTTRMSPPQQICLNSVVNTALS:NKYVKNLDELTLCSRHAGNMIPD<br>NDKNSNYKYPAVYDDKDKKCHILYIAAQENNGPRYCNKDESKRNSMFCFRPAKDISFQNYTY<br>LSKNVVDNWEKACGSGHGSGIRVDLGEDAEVAGTQYRLPSGKCPVFGKGIIIENSNTTFLTPV<br>ATGNQYLKDGGFAFPPTEPLMSPMTLDEMRHFYKD	89.2	0.897	0.855
16	*Pf*AMA1-DI-<br>Cyclic-<br>N205/K206	DITQQAKDIGAGPVASCFTTRMSPPQQICLNSVVNTALS:KYVKNLDELTLCSRHAGNMIPDN<br>DKNSNYKYPAVYDDKDKKCHILYIAAQENNGPRYCNKDESKRNSMFCFRPAKDISFQNYTYLS<br>KNVVDNWEKACGSGHGSGIRVDLGEDAEVAGTQYRLPSGKCPVFGKGIIIENSNTTFLTPVAT<br>GNQYLKDGGFAFPPTEPLMSPMTLDEMRHFYKDN	89.4	0.897	0.845
17	*Pf*AMA1-DI-<br>Cyclic-<br>K206/Y207	DITQQAKDIGAGPVASCFTTRMSPPQQICLNSVVNTALS:YVKNLDELTLCSRHAGNMIPDND<br>KNSNYKYPAVYDDKDKKCHILYIAAQENNGPRYCNKDESKRNSMFCFRPAKDISFQNYTYLSK<br>NVVDNWEKACGSGHGSGIRVDLGEDAEVAGTQYRLPSGKCPVFGKGIIIENSNTTFLTPVATG<br>NQYLKDGGFAFPPTEPLMSPMTLDEMRHFYKDNK	87.1	0.887	0.842
18	*Pf*AMA1-DI-<br>Cyclic-<br>Y207/V208	DITQQAKDIGAGPVASCFTTRMSPPQQICLNSVVNTALS:VKNLDELTLCSRHAGNMIPDNDK<br>NSNYKYPAVYDDKDKKCHILYIAAQENNGPRYCNKDESKRNSMFCFRPAKDISFQNYTYLSKN<br>VVDNWEKACGSGHGSGIRVDLGEDAEVAGTQYRLPSGKCPVFGKGIIIENSNTTFLTPVATGN<br>QYLKDGGFAFPPTEPLMSPMTLDEMRHFYKDNKY	90.1	0.897	0.856
19	*Pf*AMA1-DI-<br>Cyclic-<br>V208/K209	DITQQAKDIGAGPVASCFTTRMSPPQQICLNSVVNTALS:KNLDELTLCSRHAGNMIPDNDKN<br>SNYKYPAVYDDKDKKCHILYIAAQENNGPRYCNKDESKRNSMFCFRPAKDISFQNYTYLSKNV<br>VDNWEKACGSGHGSGIRVDLGEDAEVAGTQYRLPSGKCPVFGKGIIIENSNTTFLTPVATGNQ<br>YLKDGGFAFPPTEPLMSPMTLDEMRHFYKDNKYV	85.6	0.878	0.829
20	*Pf*AMA1-DI-<br>Cyclic-<br>K209/N2010	DITQQAKDIGAGPVASCFTTRMSPPQQICLNSVVNTALS:NLDELTLCSRHAGNMIPDNDKNS<br>NYKYPAVYDDKDKKCHILYIAAQENNGPRYCNKDESKRNSMFCFRPAKDISFQNYTYLSKNVV<br>DNWEKACGSGHGSGIRVDLGEDAEVAGTQYRLPSGKCPVFGKGIIIENSNTTFLTPVATGNQY<br>LKDGGFAFPPTEPLMSPMTLDEMRHFYKDNKYVK	87.3	0.886	0.83
21	*Pf*AMA1-DI-<br>Cyclic-<br>D227/N228	DITQQAKDIGAGPVASCFTTRMSPPQQICLNSVVNTALS:NDKNSNYKYPAVYDDKDKKCHILY<br>IAAQENNGPRYCNKDESKRNSMFCFRPAKDISFQNYTYLSKNVVDNWEKACGSGHGSGIRV<br>DLGEDAEVAGTQYRLPSGKCPVFGKGIIIENSNTTFLTPVATGNQYLKDGGFAFPPTEPLMSPM<br>TLDEMRHFYKDNKYVKNLDELTLCSRHAGNMIPD	89.2	0.896	0.859
22	*Pf*AMA1-DI-<br>Cyclic-<br>N228/D229	DITQQAKDIGAGPVASCFTTRMSPPQQICLNSVVNTALS:DKNSNYKYPAVYDDKDKKCHILYI<br>AAQENNGPRYCNKDESKRNSMFCFRPAKDISFQNYTYLSKNVVDNWEKACGSGHGSGIRV<br>DLGEDAEVAGTQYRLPSGKCPVFGKGIIIENSNTTFLTPVATGNQYLKDGGFAFPPTEPLMSPM<br>TLDEMRHFYKDNKYVKNLDELTLCSRHAGNMIPDN	90.6	0.905	0.873

23	*Pf*AMA1-DI-Cyclic-D229/K230	DITQQAKDIGAGPVASCFTTRMSPPQQICLNSVVNTALS:KNSNYKYPAVYDDKDKKCHILYIAAQENNGPRYCNKDESKRNSMFCFRPAKDISFQNYTYLSKNVVDNWEKACGSGHGSGIRVDLGEDAEVAGTQYRLPSGKCPVFGKGIIIENSNTTFLTPVATGNQYLKDGGFAFPPTEPLMSPMTLDEMRHFYKDNKYVKNLDELTLCSRHAGNMIPDND	88.2	0.888	0.849
24	*Pf*AMA1-DI-Cyclic-K230/N231	DITQQAKDIGAGPVASCFTTRMSPPQQICLNSVVNTALS:NSNYKYPAVYDDKDKKCHILYIAAQENNGPRYCNKDESKRNSMFCFRPAKDISFQNYTYLSKNVVDNWEKACGSGHGSGIRVDLGEDAEVAGTQYRLPSGKCPVFGKGIIIENSNTTFLTPVATGNQYLKDGGFAFPPTEPLMSPMTLDEMRHFYKDNKYVKNLDELTLCSRHAGNMIPDNDK	89.9	0.903	0.879
25	*Pf*AMA1-DI-Cyclic-K243/D244	DITQQAKDIGAGPVASCFTTRMSPPQQICLNSVVNTALS:DKKCHILYIAAQENNGPRYCNKDESKRNSMFCFRPAKDISFQNYTYLSKNVVDNWEKACGSGHGSGIRVDLGEDAEVAGTQYRLPSGKCPVFGKGIIIENSNTTFLTPVATGNQYLKDGGFAFPPTEPLMSPMTLDEMRHFYKDNKYVKNLDELTLCSRHAGNMIPDNDKNSNYKYPAVYDDK	88.1	0.884	0.845
26	*Pf*AMA1-DI-Cyclic-N258/G259	DITQQAKDIGAGPVASCFTTRMSPPQQICLNSVVNTALS:GPRYCNKDESKRNSMFCFRPAKDISFQNYTYLSKNVVDNWEKACGSGHGSGIRVDLGEDAEVAGTQYRLPSGKCPVFGKGIIIENSNTTFLTPVATGNQYLKDGGFAFPPTEPLMSPMTLDEMRHFYKDNKYVKNLDELTLCSRHAGNMIPDNDKNSNYKYPAVYDDKDKKCHILYIAAQENN	86.1	0.88	0.817
27	*Pf*AMA1-DI-Cyclic-G259/P260	DITQQAKDIGAGPVASCFTTRMSPPQQICLNSVVNTALS:PRYCNKDESKRNSMFCFRPAKDISFQNYTYLSKNVVDNWEKACGSGHGSGIRVDLGEDAEVAGTQYRLPSGKCPVFGKGIIIENSNTTFLTPVATGNQYLKDGGFAFPPTEPLMSPMTLDEMRHFYKDNKYVKNLDELTLCSRHAGNMIPDNDKNSNYKYPAVYDDKDKKCHILYIAAQENNG	85.0	0.871	0.809
28	*Pf*AMA1-DI-Cyclic-P260/R261	DITQQAKDIGAGPVASCFTTRMSPPQQICLNSVVNTALS:RYCNKDESKRNSMFCFRPAKDISFQNYTYLSKNVVDNWEKACGSGHGSGIRVDLGEDAEVAGTQYRLPSGKCPVFGKGIIIENSNTTFLTPVATGNQYLKDGGFAFPPTEPLMSPMTLDEMRHFYKDNKYVKNLDELTLCSRHAGNMIPDNDKNSNYKYPAVYDDKDKKCHILYIAAQENNGP	85.5	0.869	0.83
29	*Pf*AMA1-DI-Cyclic-R261/Y262	DITQQAKDIGAGPVASCFTTRMSPPQQICLNSVVNTALS:YCNKDESKRNSMFCFRPAKDISFQNYTYLSKNVVDNWEKACGSGHGSGIRVDLGEDAEVAGTQYRLPSGKCPVFGKGIIIENSNTTFLTPVATGNQYLKDGGFAFPPTEPLMSPMTLDEMRHFYKDNKYVKNLDELTLCSRHAGNMIPDNDKNSNYKYPAVYDDKDKKCHILYIAAQENNGPR	87.3	0.881	0.851
30	*Pf*AMA1-DI-Cyclic-Y262/C263	DITQQAKDIGAGPVASCFTTRMSPPQQICLNSVVNTALS:CNKDESKRNSMFCFRPAKDISFQNYTYLSKNVVDNWEKACGSGHGSGIRVDLGEDAEVAGTQYRLPSGKCPVFGKGIIIENSNTTFLTPVATGNQYLKDGGFAFPPTEPLMSPMTLDEMRHFYKDNKYVKNLDELTLCSRHAGNMIPDNDKNSNYKYPAVYDDKDKKCHILYIAAQENNGPRY	89.3	0.9	0.885
31	*Pf*AMA1-DI-Cyclic-C263/N264	DITQQAKDIGAGPVASCFTTRMSPPQQICLNSVVNTALS:NKDESKRNSMFCFRPAKDISFQNYTYLSKNVVDNWEKACGSGHGSGIRVDLGEDAEVAGTQYRLPSGKCPVFGKGIIIENSNTTFLTPVATGNQYLKDGGFAFPPTEPLMSPMTLDEMRHFYKDNKYVKNLDELTLCSRHAGNMIPDNDKNSNYKYPAVYDDKDKKCHILYIAAQENNGPRYC	82.4	0.847	0.871
32	*Pf*AMA1-DI-Cyclic-N264/K265	DITQQAKDIGAGPVASCFTTRMSPPQQICLNSVVNTALS:KDESKRNSMFCFRPAKDISFQNYTYLSKNVVDNWEKACGSGHGSGIRVDLGEDAEVAGTQYRLPSGKCPVFGKGIIIENSNTTFLTPVATGNQYLKDGGFAFPPTEPLMSPMTLDEMRHFYKDNKYVKNLDELTLCSRHAGNMIPDNDKNSNYKYPAVYDDKDKKCHILYIAAQENNGPRYCN	85.9	0.875	0.846
33	*Pf*AMA1-DI-Cyclic-K265/D266	DITQQAKDIGAGPVASCFTTRMSPPQQICLNSVVNTALS:DESKRNSMFCFRPAKDISFQNYTYLSKNVVDNWEKACGSGHGSGIRVDLGEDAEVAGTQYRLPSGKCPVFGKGIIIENSNTTFLTPVATGNQYLKDGGFAFPPTEPLMSPMTLDEMRHFYKDNKYVKNLDELTLCSRHAGNMIPDNDKNSNYKYPAVYDDKDKKCHILYIAAQENNGPRYCNK	86.8	0.884	0.826
34	*Pf*AMA1-DI-Cyclic-D266/E267	DITQQAKDIGAGPVASCFTTRMSPPQQICLNSVVNTALS:ESKRNSMFCFRPAKDISFQNYTYLSKNVVDNWEKACGSGHGSGIRVDLGEDAEVAGTQYRLPSGKCPVFGKGIIIENSNTTFLTPVATGNQYLKDGGFAFPPTEPLMSPMTLDEMRHFYKDNKYVKNLDELTLCSRHAGNMIPDNDKNSNYKYPAVYDDKDKKCHILYIAAQENNGPRYCNKD	86.1	0.88	0.814
35	*Pf*AMA1-DI-Cyclic-E267/S268	DITQQAKDIGAGPVASCFTTRMSPPQQICLNSVVNTALS:SKRNSMFCFRPAKDISFQNYTYLSKNVVDNWEKACGSGHGSGIRVDLGEDAEVAGTQYRLPSGKCPVFGKGIIIENSNTTFLTPVATGNQYLKDGGFAFPPTEPLMSPMTLDEMRHFYKDNKYVKNLDELTLCSRHAGNMIPDNDKNSNYKYPAVYDDKDKKCHILYIAAQENNGPRYCNKDE	87.1	0.885	0.822
36	*Pf*AMA1-DI-Cyclic-S268/K269	DITQQAKDIGAGPVASCFTTRMSPPQQICLNSVVNTALS:KRNSMFCFRPAKDISFQNYTYLSKNVVDNWEKACGSGHGSGIRVDLGEDAEVAGTQYRLPSGKCPVFGKGIIIENSNTTFLTPVATGNQYLKDGGFAFPPTEPLMSPMTLDEMRHFYKDNKYVKNLDELTLCSRHAGNMIPDNDKNSNYKYPAVYDDKDKKCHILYIAAQENNGPRYCNKDES	83.1	0.855	0.82
37	*Pf*AMA1-DI-Cyclic-K269/R270	DITQQAKDIGAGPVASCFTTRMSPPQQICLNSVVNTALS:RNSMFCFRPAKDISFQNYTYLSKNVVDNWEKACGSGHGSGIRVDLGEDAEVAGTQYRLPSGKCPVFGKGIIIENSNTTFLTPVATGNQYLKDGGFAFPPTEPLMSPMTLDEMRHFYKDNKYVKNLDELTLCSRHAGNMIPDNDKNSNYKYPAVYDDKDKKCHILYIAAQENNGPRYCNKDESK	83.9	0.859	0.833
38	*Pf*AMA1-DI-Cyclic-R270/N271	DITQQAKDIGAGPVASCFTTRMSPPQQICLNSVVNTALS:NSMFCFRPAKDISFQNYTYLSKNVVDNWEKACGSGHGSGIRVDLGEDAEVAGTQYRLPSGKCPVFGKGIIIENSNTTFLTPVATGNQYLKDGGFAFPPTEPLMSPMTLDEMRHFYKDNKYVKNLDELTLCSRHAGNMIPDNDKNSNYKYPAVYDDKDKKCHILYIAAQENNGPRYCNKDESKR	85.6	0.873	0.798
39	*Pf*AMA1-DI-Cyclic-N271/S272	DITQQAKDIGAGPVASCFTTRMSPPQQICLNSVVNTALS:SMFCFRPAKDISFQNYTYLSKNVVDNWEKACGSGHGSGIRVDLGEDAEVAGTQYRLPSGKCPVFGKGIIIENSNTTFLTPVATGNQYLKDGGFAFPPTEPLMSPMTLDEMRHFYKDNKYVKNLDELTLCSRHAGNMIPDNDKNSNYKYPAVYDDKDKKCHILYIAAQENNGPRYCNKDESKRN	85.6	0.872	0.799
N.A. = Cut site position is not applicable as the structure is linear, therefore, applied any cyclic offset.<br>* = As this is a linear structure, the model was predicted using ColabFold v1.5.5 (AlphaFold2-MMseqs2), without applying any cyclic offset.					
